# Application of real-time PCR for the identification of the endangered species *Galemys pyrenaicus* through faecal samples

**DOI:** 10.1007/s11033-023-09010-2

**Published:** 2024-01-05

**Authors:** Adriana Ripa, José A. Díaz-Caballero, María Jesús Palacios-González, Antonio Espinosa, Juan Luis García-Zapata, José Luis Fernández-Garcia

**Affiliations:** 1 Genetic and Animal Breeding, Faculty of Veterinary, Universidad de Extremadura, 10071 Cáceres, Spain; 2 Dirección General Sostenibilidad, Consejería Para La Transición Ecológica y Sostenibilidad, Junta de Extremadura, 06800 Merida, Spain; 3 Department of Mathematics, Universidad de Extremadura, 06006 Badajoz, Spain; 4 Área del Medio Natural, Sociedad de Gestión Pública de Extremadura (GPEX), Junta de Extremadura, Mérida, España

**Keywords:** *Galemys pyrenaicus*, Conservation, RT-PCR, Identification, Non-Invasive samples

## Abstract

**Background:**

Currently, many micromammals are important targets for study. The endangered *Galemys pyrenaicus* is an outstanding example. Globally, their populations have suffered a substantial decline in last 20 years. In the surveyed area, the capture of desman is legally forbidden due to the high conservation concerns. Reason by non-invasive sampling through faeces is proposed for its monitoring. Furthermore, the confusion between faeces from desman and Mediterranean water shrews must be considered. Thus, the aim of this study was focused on developing RT-PCR assays to determine the presence of *Galemys pyrenaicus* and *N. a. anomalus* from non-invasive samples.

**Methods and results:**

The study was conducted in the mountains of the System Central of Extremadura (Spain). A total of 186 samples were collected from 2018 to 2021 by experts where historically reported and/or our previous studies confirmed their presence. RT-PCR assays using hydrolysis probes were designed to detect genetic material from both desman and Mediterranean water shrews and its specificity was confirmed. The reliability of the method was further assessed by PCR sequencing of mitochondrial Cyb and d-loop, resulting fully compatible with the RT-PCR approach. Intraspecific phylogenetic relationship was reported to improve knowledge about mtDNA variability in the desman from the Central System.

**Conclusions:**

We demonstrated that RT-PCR gives a gold opportunity to further map the species using faeces which minimizes disturbance and reports both population status and individual presence. Cost-effective RT-PCR combined with field-collected faeces allows us to better investigate the full range of occurrence of the species.

**Supplementary Information:**

The online version contains supplementary material available at 10.1007/s11033-023-09010-2.

## Introduction

The Iberian desman (*Galemys pyrenaicus,* E. Geoffroy Saint-Hilaire 1811) is one of the endemic mammals in the Iberian Peninsula with exceptional value from the point of view of evolution and biodiversity, despite being the only representative of its genus (*Galemys*) worldwide. The International Union for Conservation of Nature (IUCN) lists the desman under the category of endangered (last accessed September-2023) [1], due to a marked reduction in its distribution area and degree of isolation over the last 20 years, especially with relict populations in the Central System mountains [2]. In an international context, European Community environmental legislation considered the desman as a wild species deserving strict protection (Annex II of the Bern Convention on the Conservation of European Wildlife and Natural Habitats) and Directive 92/43/CEE for flora and wildlife conservation included the Iberian desman in Annex II (species whose conservation needed special areas of conservation) and Annex IV (species requiring strict protection). In the local context (Spain), the species has been included in the List of Wild Species under Special Protection Regime (LESRPE) and the list of threatened Species (Royal Decree 139/2011, February), where the populations of the Central System are found as "in danger of extinction". This worries many researchers about the possibility of losing some of their local populations. [1, 3–6]. Because conservation efforts critically depend on a detailed knowledge of populations [7] the approved Recovery Plan for the Iberian desman in Extremadura territories (Spain) promoted monitoring and research activities based on non-invasive techniques and subsequent determination of the species through species-specific genetic testing.

The Iberian desman have strict biotic requirements to live in typical mountain habitats, as they only inhabit streams that flow with clean, cold and good oxygenated water and rich in benthic invertebrates, all these characteristics make them such a unique species [6, 8, 9].

Non-invasive samples have been useful in the study of genetic variability [10]. Especially, cytochrome b (Cyb) gene studies permitted regrouping the Iberian desman into two well defined big clades (A and B) and four phylogenetic lineages, with discrete distribution, but only one population was declared to be admixed at the contact zone [8]. This molecular signature has proven to be useful for unambiguously ascribing them to discrete geographic localities using genetic material from faeces [8, 11]

Live trapping is not advisable in species highly vulnerable to human handling [12] which could be a potential risk for the survival of individuals. Thus, authorities with competence on wildlife limited sampling to faeces collection (Order of 3 August 2018 approving the Recovery Plan for the Iberian desman (Galemys pyrenaicus) in Extremadura. In recent times, a generalization of non-invasive sampling within research methods has been witnessed, including monitoring the health status of the desman [13]. However, these non-invasive sample were difficult to distinguish macroscopically among semi-aquatic micromammals species sharing habitats [14]. Especially in Extremadura, it was expected that faeces from the Iberian desman would be confused with those belonging to Mediterranean water shrews (*Neomys anomalus anomalus*). This was the reason why a wrong diagnosis compromised any research if samples may be unproperly assigned to another species [13]. Previous studies [15] have suggested that Iberian desman can be easily detected by live sampling or macroscopic laboratory analysis of faeces through food remains and hairs.

Accordingly, the development of non-invasive methods and molecular diagnostics provides a golden opportunity to further map desman populations because such procedures minimize disturbances and efficiently monitor different wildlife species, as demonstrated in other wild or rare species [16–19]. Furthermore, the molecular procedures must have into account confusion sources when similar species share habitat [11, 17, 20, 21]. Regarding the Iberian desman search, it was justified to exclude the Mediterranean water shrew (*N. a. anomalus*, Cabrera 1907), a palearctic species widely distributed in the water environment of the Iberian Peninsula, including Andalusia and Extremadura (Spain) [22].

There are assays that describe methodologies for determining the species origin of the faeces [11 and references therein] in the context of desman conservation. The PCR–RFLP method designed to detect species-specific targets, although useful, needs successive analytical rounds to be conclusive [11]. For example, Leone et al. [23] described a PCR–RFLP with a two phase procedure that included nesting, before restriction enzyme digestion. However, ongoing molecular techniques based on real time PCR (RT-PCR) are being claimed for their best sensitivity, specificity, reproducibility and multiplexing facility. Elyasigorji [24] noted that elusive and poorly identified species by other methods can be investigated in depth by DNA or protein analysis using molecular techniques such as RT-PCR. After the COVID-19 pandemic, RT-PCR was assumed worldwide as the gold standard for the diagnosis of the virus and has effectively contributed to its control. Current applications of RT-PCR include gene expression analysis, mutation detection, pathogen detection and quantification, detection of genetically modified organisms, detection of allergens, monitoring of microbial degradation, species identification and determination of parasitic fitness [13, 25]. This powerful tool, with high precision technology, is efficient, simple, medium cost, rapid and results in high sensitivity and specificity, offering great scope for quantitative and qualitative detection of species [26]. However, this molecular method requires strict and fine controls to avoid unspecific amplification of the specific target because many of micromammal shares evolutionary neighbor genetic lineages [14, 27].

The aim of this study was focused on to developing molecular assays based on RT-PCR to determine the absence or presence of *Galemys pyrenaicus* from non-invasive samples, especially faeces, and at the same time to differentiate it from *N. a. anomalus* which shares an ecological niche with the desman in Extremadura). Furthermore, the developed assays are helping us in recovery plans for the species. The application of the RT-PCR technique provided the tools requested by the administrations for the projects and monitoring arrangements within the "Desman (*Galemys pyrenaicus*) Recovery Plan in the Central System (Iberian Peninsula) in Extremadura.

## Materials and methods

### Study area and sampling

The study was conducted in the autonomous community of Extremadura (Spain) in the western part of the System Central Mountains of the Iberian Peninsula. All samples were collected from 2018 to 2021.

The faeces that were macroscopically identified of supposedly desman (n = 186), and one tissue sample (intestine) from a male desman that was found dead (La Vera population) used as a PCR control [13], were exhaustively collected according to the shape, size, colour, and texture (acquired by feeding) by qualified personnel from the three geographical protected areas along the riverbeds in Ambroz valley, Jerte valley and La Vera (Tietar valley) as a part of the monitoring project according to the “Recovery Plan for the Desman” (*Galemys pyrenaicus*) in the Central System (Iberian Peninsula) in Extremadura (DOE 158 08/14/2018). However, both morphological and sensorial characteristics of faeces were not reported by the sender to emulate a blind trial. Even if the faeces are collected as fresh as possible because adverse weather conditions can damage the DNA [28, 29], RT-PCR was always carried out. All samples were geotagged, but by the decision of the regional government, the availability of the geolocation data was restricted to avoid public knowledge due to the worrying state of conservation of the species in the sampling area.

The samples were transported in sterile tubes containing ethanol (96% or higher) and stored at − 20 °C during collection in the field and at − 70 °C upon transfer to the laboratory.

### DNA extraction

DNA fecal samples were isolated using the QIAamp® Fast DNA Stool Mini Kit (QIAGEN GmbH, Hilden, Germany) following the manufacturer’s instructions and a UV treated room. However, tissues from the desman and one Mediterranean shrew also found dead were extracted using a fast salting out procedure [30]. A mixture of these DNAs was used as a positive control. Faecal and tissue DNA were extracted in separate dedicated laboratories. Furthermore, DNA extractions were assessed by quantification using a Qubit 4 Fluorometer (Thermo Fisher Scientific, Waltham, MA, USA) and stored at − 70 °C until use.

### *Galemys pyrenaicus* RT-PCR design, assay, and further sequencing

Hydrolysis probe RT-PCR assays were designed to detect genetic material of the desman and Mediterranean water shrew based on previous knowledge in Fernández-García and Vivas [11]. Initially, the assay was developed using Cyb and 12sDNA genes from NCBI data. Cyb and 12 sRNA gene sequence were further analysed “in silico” by BLAST adjusting the algorithm to 5000 maximum target sequences using one portion of the Cyb sequence to each species or 12 sDNA gene for *G. pyrenaicus* (identity percent and taxonomy records details are shown in Supplementary material, Table S1 a to c). External primers and hybrid internal oligonucleotides were designed in Primer3 plus software [31] for each species as shown in Table 1, which may work at high specificity in multiplex assays (as for CYBgal and CYBneo) or single (12sRNA) RT- RCR using degraded DNA. Then, primers and probes were re-analysed by BLAST search and TaqMan probes were ordered with a QSY quencher as recommended by the manufacturer for multiplexing (Thermo Fisher Scientific—US).

**Table 1:** Primers and hybrid hydrolysis probes

Species	Gene	External primer pair	Hybrid oligo	Size
*G. pyrenaicus*	CYBgal	GalF.5’ACATGAAATATCGGCGTCCTGT3’	GalI.5’GCCACCGCATTCATAGGGTACGT	137 bp
		GalR.5’CCGATGTAAGGGATGGCTGA3’		
*G. pyrenaicus*	12sRNA	12sGalF.5’TGGGAAGMAATGGGCTACA3’	12sGalI.5’AATTTAACGAAAACCTTCATGA	141 bp
		12sGalR.5’GGTGTGTRCGTRCTTCAT3’		
*N. a. anomalus*	CYBneo	NanomaF.5’TGAATTTTAGTAGCGGACCTTATT3’	NeoI.5’GAATTGGAGGCCAACCAGT	94 bp
		NanomaR.5’GGATGGAAGCTAGTTGTCCAATA3’		

All assays were set up and run using the standard curve mode in Step One™ software using a Step One Plus equipment (Applied Biosystems, Thermo Fisher Scientific-US). The RT-PCR reactions followed the protocol described in Ripa [13] using 5 μl of undiluted DNA extract. Finally, a presence/absence procedure for each sample was performed using Design and Analysis Software ver 2.2.1 (Thermo Fisher Scientific-US). A positive cut-off cycle threshold value was considered from Ct ≤ 38 in any case and the negative cut-of cycle Ct threshold value > 38, but double amplification (treated as contaminated) samples were eliminated from further analysis.

Furthermore, all positive samples (from the three populations) were further amplified and Sanger sequenced for at least one of the segments 1, 2 and 3 for the mitochondrial Cyb and d-loop genes, respectively, to assess clade following Igea [8] and Querejeta [2]. These data sets were aligned in MEGA 5.0 [32] and sequences with complete data were used to find similar sequences using BLAST at NCBI. Mainly, we collected sequences from the two studies cited above sharing maximum coverage for both the Cyb and the d-loop (1063 bp joining 724 bp from 14,629 to 15,352 Cyb and 339 bp from 15,491 to 15,829 d-loop, respectively, with respect to the reference sequence AY833419.1) were concatenated and considered for network construction [33]. A total of 30 sequences were downloaded from GeneBank (identical sequences collapsed to one) but 9 additional sequences from this study met this criterion. The concatenated sequences were used to map the distribution of genetic variants and their phylogenetic relationship with each other. This data set was run with the ‘star contraction algorithm’ to collapse haplotypes into one haplogroup and, after, with the “median-joining (MJ) network” procedure [33], implemented in NETWORK 4.1.1.1 (www.fluxus-technology.com) displaying the structure of the network.

## Results

The specificity of the method to detect the target was assayed using individual samples of different species from our laboratory (Table 2), but DNA of all micromammal species inhabiting near the sampling territory such as *T. occidentalis* and *N.* a*. anomalus*, were included. All non-target species for each assayed species were negative, except *T. occidentalis* with was amplified at Ct = 37.340 for the CYBgal assays with 10 ng total DNA. This was the reason why the 12sRNA assay was also used as alternative protocol for the validation of *G. pyerenaicus*. The 12sRNA assay was fully negative for *T. europaea*. No sequences from *G. pyrenaicus* and *N. a. anomalus* produced significant alignment for CYBneo and CYBgal targets, respectively (Supplementary material S1). Although at high Ct value, positive results for CYBgal assays in *T. occidentalis* may be explained (Supplementary material, Table S1 a) because the sequences of that species were the nearest with higher significant alignment identity (91.97 to 90.27%) with respect to the Cyb reference sequences of the desman (Supplementary material, Table S1 a). Sequences from *Talpa spp* and *Neomys a. anomalus* also produced significant alignment (< 93,63% similarity) for the 12 sDNA assay, but this test showed better specificity (Table 2). In the CYBneo test only the target species were positive (Table 2), although the assays were also run as multiplex RT-PCR from genetic material of faeces (Table 3). No significant alignments were observed for *Talpa* spp. or for *G. pyrenaicus* (Supplementary material, Table S1 b).

**Table 2:** Specificity test. RT-PCR with 42 cycles

DNA species	CYBgal	CYBneo	12sRNAgal
*Oryctolagus cuniculus (n* = *2)*	−	−	−
*Neovison vison (n* = *1)*	−	−	−
*Canis lupus familiaris (n* = *3)*	−	−	−
*Mus musculus (n* = *1)*	−	−	−
*Lynx pardinus (n* = *2)*	−	−	−
*Homo sapiens (n* = *1)*	−	−	−
*Bos taurus (n* = *2)*	−	−	−
*Felis spp (n* = *2)*	−	−	−
*Lutra lutra (n* = *2)*	−	−	−
*Ovis aries (n* = *2)*	−	−	−
*Talpa occidentalis (n* = *1)*	+ (37)	−	−
*Neomys anomalus (n* = *2)*	−	+	−
*Ciconia Ciconia (n* = *1)*	−	−	−
*Galemys pyrenaicus (n* = *7)*	+	−	+
Negative control	−	−	−

− = negative and +  = positive result for cut-off minor than Ct 38, respectively. (37) = Ct for *Talpa occidentalis*

**Table 3:** Non-invasive sampling effort within population locations and total. From the second to the last column: Number of samples, positive for G. pyrenaicus (desman), positive for N. anomalus (Mediterranean water shrews), contaminated (showing both targets) and empty (no amplification). Percentages in parentheses

	Sampling effort	Positive Galemys	Positive Neomys	contaminated	empty
La vera (%)	71	56(78.87)	5(7.04)	2(2.82)	8(11.27)
Jerte valley (%)	38	38(100)	0(0)	0(0)	0(0)
Ambroz valley (%)	77	70(90.91)	3(3.90)	1(1.30)	3(3.90)
Total (%)	186	164(88.17)	8(4.30)	3(1.61)	11(5.91)

Furthermore, the 186 faecal pellets collected from the three desman populations in Extremadura successfully yielded DNA ranging from 0.1 to 10 ng/mL. The presence of amplification using the Ct 38 cut-off value was undoubtedly assigned to the desman target in 88.17% of them. Table 3 shows the results from each population from Ambroz Valley, Jerte Valley and La Vera, as described in Ripa [13]. All positive samples for desman were further validated using 12sRNA assays with the same result. Several DNA extracts showed *N. a. anomalus* genetic materials and contaminations from both species (Table 3). Non positive samples could be mistakenly collected as faeces from the desman.

**Fig. 1:**
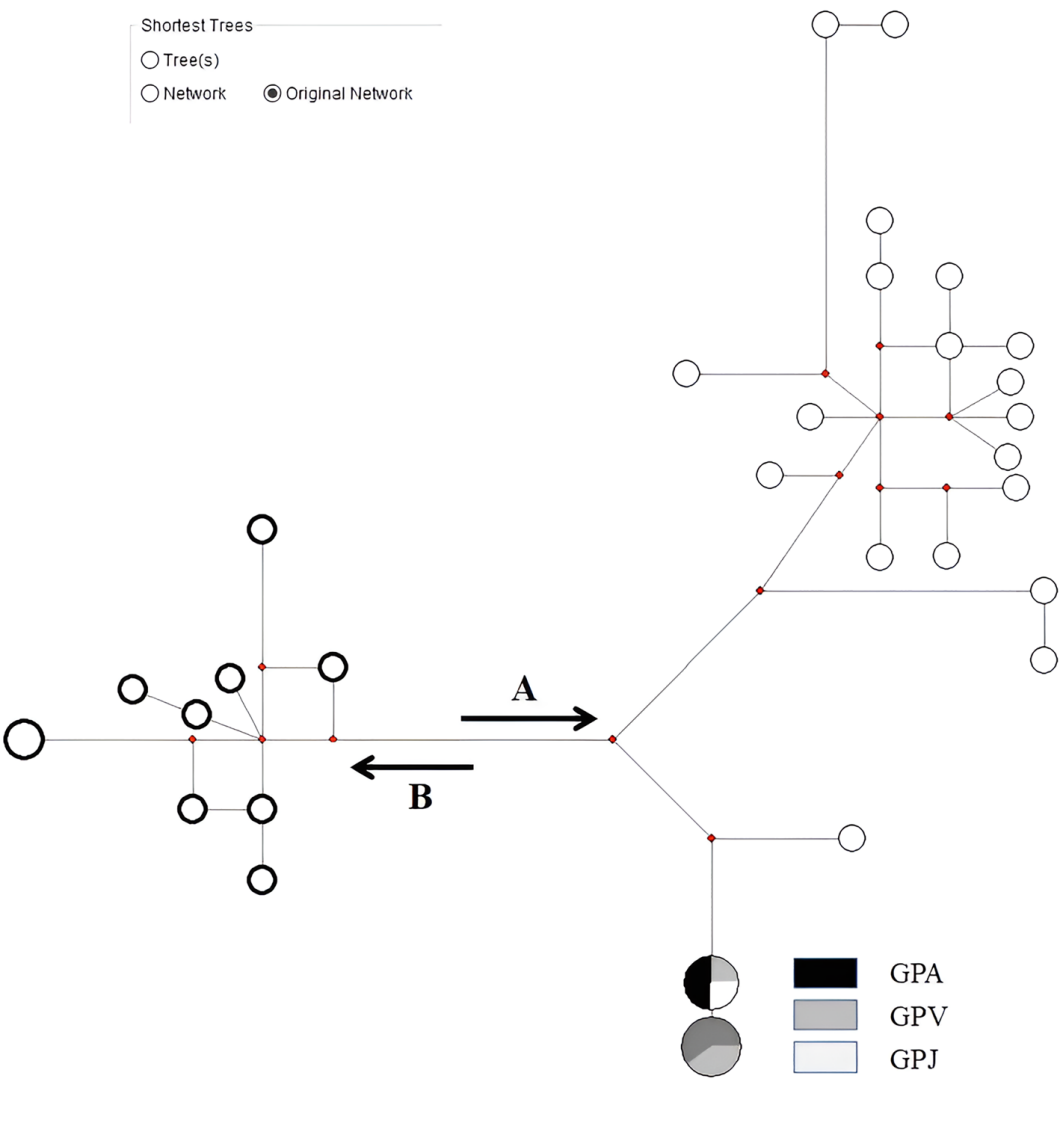
Original Network of the Shortest Median-Joining trees. Empty dots indicate sequences from Gene Bank except one (see text). The network draws two opposites branches for Clades A and B according to Igea [8] and Querejeta [2] (**A **and **B**). GPA, GPV and GPJ indicate sequences of the populations located in the Ambroz Valley, La Vera and Jerte Valley, respectively (populations from Tajo basin, Extremadura autonomous community, Spain). In this last case node size is proportional to sequence number

The reliability of the method was also assessed by PCR of Cyb and d-loop. Not all samples yielded positive products but, globally, the results were compatible with the RT-PCR approach. Furthermore, 34, 100, 70 and 38 positives PCR were sequenced using segments 1, 2 and 3 of the Cyb and d-loops, respectively. However sequencing success for all four mtDNA portions was obtained in nine samples. A unique haplotype was always found for all populations sampled with respect to Cyb. Partially or completely, the gene sequence of Cyb was fully concordant with Clade A2 described by Igea [8] for the mountains of the Sistema Central but systematically one sole haplotype. Instead, the d-loop showed two haplotypes that differed by only one nucleotide (complete reference sequence AY833419.1: 15,666; changed nucleotide A/G). Specifically, one of them was exclusive in samples from Ambroz (A), and the other was from samples collected in Jerte (G) and both in La Vera, opening the issue about genetic contact between the most distant away populations in the Extremadura territory (Fig. 1). However, there is no explanation for deciphering this connection yet. Although somewhat partial, all sequences sharing 100% molecular coverage for both the Cyb and the d-loop following descriptions in materials and methods were concatenated and considered for network construction [33]. A total of 30 sequences were downloaded from Gene bank (identical sequences collapsed to one) but 8 additional sequences from this study met this criterion. The concatenated sequences were used to map the distribution of genetic variants and their phylogenetic relationship with each other (Fig. 1). The haplotype located exclusively in Ambroz was interior with respect to the exclusive haplotype (tip) in Jerte. Furthermore, only sequences from the Central Systems Mountains regrouped to the interior one (Ac. N. JX290647; [8]).

## Discussion

Research studies focusing on the non-invasive genetics of meso- and micromammals are still limited but very often share molecular methods allowing the unequivocal identification of sympatric species in focus [18, 34]. In this line, this study substantiated the development of three RT-PCR approaches that facilitate high-throughput analysis of degraded DNA from non-invasive samples. To our knowledge, this is the first study to identify *Galemys pyrenaicus* by RT-PCR in faeces. In addition, the simultaneous detection of other micromammals has been included, especially those whose size, appearance, behaviour, environmental similarity, and trophic spectrum. In particular, the most direct competitor species of the most southwestern Iberian Peninsula desman populations such as *N. a. anomalous*.

### RT-PCR method for species-specific monitoring

As mentioned above, one of the requirements of the recovery plan of desman from the Sistema Central Mountain was to have effective molecular methods to replace the more subjective morphological identification of faeces, first used for monitoring small semi-aquatic micromammals. The high percentage of positive samples was satisfactory to verify, presence and although faeces were collected without taking into account ageing or exposure to adverse weather conditions [29], this does not seem to compromise the success of the assay in our case.

Zarzoso-Lacoste [35] highlighted RT-PCR as a critical component for the detection of samples according to the criteria used together with the selection of specific primers and probes, mainly with low amounts of target DNA. In support of technical decisions, as much prior “in silico” analysis as possible should be carried out to ensure specificity or to help develop alternative primers and/or hydrolysis probes to avoid some misidentification pitfalls, but experimental testing is always recommended (this study). In addition, RT-PCR with hydrolysis probes improved specificity and sensitivity because both parameters can be controlled and multiplexing can also be performed, allowing double-checking of the target DNA and rapid diagnosis [36]. In addition, its application has been very useful to screen large amounts of faeces composed even of mixed DNA [35], such as when different targets are found within the sample in latrines and, although it was not very common, to detect possible interspecies contamination (1,61% in this study).

Most non-invasive genetic studies have focused on large wild mammals [17, 21], but studies of small mammals can be extrapolated because the results appear to be very sensitive [18, 19].

The use of double-check desman-specific primers/probes based on the 12 sRNA and CYBgal/CYBneo assays may be considered a successful and robust method as well as a high throughput way to trace the presence/absence of this species in its potential habitats. The high percentage of positive samples for the target species and competing species indicated suitable yields [37].

A positive result with enough *T. europea* DNA occurred at the previous Ct to the cut-off value. However it should be aware that desman put its droppings in stony streams and moles preferred meadows, fields, plains, and gardens to avoid stony soils [38] which left little doubt in our study. If doubts remain, the alternative is to use the 12 sRNA assay when moles and desmans share habitat or as needed at research criteria.

### Advantages of RT-PCR over conventional methods

Conventional or nested PCR using the Cyb gene has been successfully applied to *G. pyrenaicus*, *Neomys fodiens* and *Cinclus cinclus* using faecal samples with a rate of 90% [39]. Thus, this confirms to the reliability of molecular methods based on mtDNA for this kind of task. However, the combination of the several restriction enzyme patterns (RFLP- PCR) was needed for a robust diagnostic as deduced from all known sequences of the Iberia desman described [11]. Even sequencing of the PCR products obtained was proposed because it allowed avoiding a misidentification of almost 20% of the collected faecal samples [39] which implies a labour-intensive and costly approach. Sequencing could be ruled out if the specificity and sensitivity of RT-PCR has been experimentally proven unless other objectives were focused such as phylogenetic studies. Furthermore, its high sensitivity can also prevent the waste of a large part of the extracted DNA. Requires additional genetic material, as well as identifying poor quality samples that are unlikely to produce results with other techniques.[40, 41]. In addition, to the specificity and sensitivity of the RT-PCR assay, Ruiz-González [34] claimed the high speed and automation possibility and the quality control to select optimal DNA samples.

### Haplotype findings

Regarding the star-contracted MJ network, the downloaded sequences and those obtained in this study split the haplotypes of the Iberian desman into two well defined branches named Clades A and B as previously described [2, 8]. As expected, the two haplotypes (nodes) from Extremadura showed a conspicuous separation within clade A, named subclade A2 in Igea [8] but lacking in Querejeta [2]. For the first time, the difference between these two haplotypes was due to a unique polymorphic site in the segment of the d-loop (see details in results). This was suggestive of low mitochondrial variability in the studied population, with an apparent association of haplotypes with geography. The contrast between interior and tip haplotypes (ancestral versus derived haplotypes, respectively) could be considered a hallmark of the latest distinguishable split in the Central System at Extremadura (Spain). In this sense, the youngest haplotype was found only in Jerte Valley and La Vera (Fig. 1). However, the interior haplotype was only exclusive in Ambroz Valley (south-westernmost population in the entire distribution of the species) which was identical to the one found on the northeast side of Central System (on the opposite side in Sierra de Gudarrama, Tajo Basin, Spain) (NCBI Ac. N. JX290647; [8]).

It has been suggested that incomplete lineage sorting or introgression following secondary contact may be causes of retention of ancestral polymorphisms [42]. However, it is difficult to choose either, as both generate similar patterns of shared genetic diversity, especially when variability is very low. In addition, less probable events, such as anthropogenic translocations, should not be forgotten. The incidence of derived haplotypes (e.g., tips) is essential when trying to accurately infer the dispersal history of species with overlapping distributions [2]. A pattern with older (interior) haplotypes typically found at the most distant sites of the Tajo basin, provides indications for site refugia and from where subsequently the tip haplotypes of the species expanded in Extremadura. Private haplotype distributions with interior and tip at Ambroz and Jerte Valleys, respectively, explain the current proneness to fragmentation of this specialist species [43]. Thus, a widespread status of at most a single founder female can occur under restricted dispersal because it favors the lack of admixtures of mitochondrial types even without a visible geographic barrier [2] causing mtDNA monomorphism by drift and lineage sorting [44]. Whereas Cyb was uninformative, the d-loop data seem to suggest that each population remained isolated so long as inevitably alternative haplotypes were fixed after mutation, drift, and lineage sorting for the neighboring Ambroz and Jerte valley, respectively. Thus, a connection loss in high mountains is supported as suggested by Querejeta [2]. However, La Vera population was a particular case that deserve in-depth study, which is ongoing because the assignment of species from the resampling trials is now easier.

### Applicability to population monitoring in threatened desman.

Recent studies have claimed for a greater research effort on endangered species such as the Iberian desman. In particular, those populations inhabiting high mountains of the Pyrenees, of Cantabrian Mountains, or the Central System, because connections thorough its basins and sub-basins have been suggested as rare or extremely difficult [2]. Focusing on this need, the use of mtDNA genes to track the desman throughout biological waste [10]. RT-PCR also helps address other relevant questions. Once the species is confirmed, it is also possible to study genetic variation in another class of genes, in addition to mtDNA, which is more suitable for studying dispersal, gene flow and inbreeding [2, 10] including the identification and distribution of females and males using only their feces. In Ripa [13] without a doubt the identification by RT-PCR through feces was a cornerstone in the objective of investigating the health status within the conservation strategies of this emblematic species.

## Conclusion

Our study provided several cost-effectively RT-PCR which has been combined with expert field-collected faeces to better investigate the whole range of the desman specie and other species that compete for resources in the mountains of the Central Systems. This non-invasive technique is a very valuable tool for the implementation of the recovery plan of the Iberian desman, and it can be very helpful in the implementation of monitoring of other wildlife species.

## Supplementary Information

Below is the link to the electronic supplementary material.

Supplementary file1 (DOC 2167 kb)

## Data Availability

The data presented in this study are available on request from the author.

## References

[1] Quaglietta L, Galemys Pyrenaicus (Versión Modificada de la Evaluación de 2021). La Lista Roja de Especies Amenazadas de la UICN 2022 2021. Available online: www.iucnredlist.org. Accessed 9 Dec 2021

[2] Querejeta M, Fernández-González A, Romero R, Castresana J (2017) Postglacial dispersal patterns and mitochondrial genetic structure of the Pyrenean desman (*Galemys pyrenaicus*) in the northwestern region of the Iberian Peninsula. Ecol Evol 7(12):4486–4495 28649358 10.1002/ece3.3034 PMC5478051

[3] Aymerich P, Casadesús F, Gosàlbez J (2002) Factors de distribució de Galemys pyrenaicus a Catalunya. Orsis 17:21–35

[4] Charbonnel A, Buisson L, Biffi M, d’Amico F, Besnard A, Aulagnier S et al (2015) Integrating hydrological features and genetically validated occurrence data in occupancy modelling of an endemic and endangered semi-aquatic mammal, *Galemys pyrenaicus*, in a Pyrenean catchment. Biol Conserv 184:182–192

[5] Charbonnel A, Laffaille P, Biffi M, Blanc F, Maire A, Nemoz M et al (2016) Can recent global changes explain the dramatic range contraction of an endangered semi-aquatic mammal species in the French Pyrenees? PLoS ONE 11(7):e0159941 27467269 10.1371/journal.pone.0159941 PMC4965056

[6] Nores C, Salvador Milla A (2017). Desmán ibérico–Galemys pyrenaicus (E. Geoffory, 1811).

[7] Frankham R (2003) Genetics and conservation biology. CR Biol 326:22–29 10.1016/s1631-0691(03)00023-4 14558445

[8] Igea J, Aymerich P, Fernandez-Gonzalez A, Gonzalez-Esteban J, Asuncion Gomez RA, Gosalbez J, Castresana J (2013) Phyleography and postglacial expansion of the endangered semi-aquatic mammal Galemys Pyrenaicus. BMC Evol Biol 13(115):1–19 23738626 10.1186/1471-2148-13-115 PMC3682870

[9] Aymerich P, Gosálbez J (2014) El desmán ibérico Galemys pyrenaicus (É. Geoffroy Saint-Hilaire, 1811) en los Pirineos meridionales. Munibe Monogr Nat Ser 3:37–77

[10] Escoda L, Castresana J (2021) The genome of the Pyrenean desman and the effects of bottlenecks and inbreeding on the genomic landscape of an endangered species. Evol Appl 14(7):1898–1913 34295371 10.1111/eva.13249 PMC8288019

[11] Fernández-García JL, Cedillo MDPV (2017) Faecal DNA template as non-invasive tools in order to distinguish the endangered Pyrenean desman (*Galemys pyrenaicus*, Eulipotyphla, Talpidae) from Mediterranean water shrews (*Neomys anomalus*, Soricomorpha, Soricidae). Hystrix 28(1):92

[12] Gillet F, Tiouchichine ML, Galan M, Blan F, Némoz M, Aulagnier S, Michaux JR (2015) A new method to identify the endangered Pyrenean desman (*Galemys pyrenaicus*) and to study its diet, using next generation sequencing from faeces. Mamm Biol 80:505–509

[13] Ripa A, Díaz-Caballero JA, Palacios-González MJ, Zalba J, Espinosa A, García-Zapata JL et al (2023) Non-invasive wildlife disease surveillance using real time PCR assays: the case of the endangered *Galemys pyrenaicus* populations from the central system mountains (Extremadura, Spain). Animals 13(7):1136 37048392 10.3390/ani13071136 PMC10093302

[14] Castresana J, Igea J, Aymerich P, Fernandez-Gonzalez, Gosalbez J. (2009). Filogeografía del Desmán Ibérico (Galemys pyrenaicus) y su distribución en la red de Parques Nacionales. *Proyectos de Investigación en Parques Nacionales* 143–154.

[15] González-Esteban J, Villate I, Castién E (2003) Sexual identification of *Galemys pyrenaicus*. Acta Theriol 48:571–573

[16] Stenglein JL, Waits LP, Ausband DE, Zager P, Mack CM (2010) Efficient, noninvasive genetic sampling for monitoring reintroduced wolves. J Wildl Manag 74(5):1050–1058

[17] Buglione M, Petrelli S, de Filippo G, Troiano C, Rivieccio E, Notomista T et al (2020) Contribution to the ecology of the Italian hare (*Lepus corsicanus*). Sci Rep 10(1):13071 32753640 10.1038/s41598-020-70013-1 PMC7403147

[18] Buglione M, Petrelli S, Notomista T, de Filippo G, Gregorio R, Fulgione D (2020) Who is who? High resolution melting analysis to discern between hare species using non-invasive sampling. Conserv Genet Resour 12:727–732

[19] Buglione M, Troisi SR, Petrelli S, van Vugt M, Notomista T, Troiano C et al (2020) The first report on the ecology and distribution of the wolf population in cilento, Vallo di Diano and Alburni National Park. Biol Bull 47:640–654. 10.1134/S1062359021010040

[20] Letten AD, Ke PJ, Fukami T (2017) Linking modern coexistence theory and contemporary niche theory. Ecol Monogr 87(2):161–177

[21] Buglione M, Petrelli S, Troiano C, Notomista T, Petrella A, De Riso L et al (2020) Spatial genetic structure in the Eurasian otter (*Lutra lutra*) meta-population from its core range in Italy. Contrib Zool 90(1):70–92

[22] Haberl W (2022) Comparative measurements of running speed in five species of European shrews (Soricidae, Eulipotyphla). J Wildl Biodiversity 6(1):102–104

[23] Leone A, Ripabelli G, Sammarco ML et al (2009) Detection of *Cryptosporidium* spp. from human faeces by PCR-RFLP, cloning and sequencing. Parasitol Res 104:583–587. 10.1007/s00436-008-1233-8) 18979120 10.1007/s00436-008-1233-8

[24] Elyasigorji Z, Izadpanah M, Hadi F, Zare M (2023) Mitochondrial genes as strong molecular markers for species identification. Nucleus 66(1):81–93

[25] Artika IM, Dewi YP, Nainggolan IM, Siregar JE, Antonjaya U (2022) Real-time polymerase chain reaction: current techniques, applications, and role in COVID-19 diagnosis. Genes 13(12):2387 36553654 10.3390/genes13122387 PMC9778061

[26] Yang L, Xu S, Pan A, Yin C, Zhang K, Wang Z et al (2005) Event specific qualitative and quantitative polymerase chain reaction detection of genetically modified MON863 maize based on the 5 ‘-transgene integration sequence. J Agric Food Chem 53(24):9312–9318 16302741 10.1021/jf051782o

[27] Lopez-de Sancha A, Roig R, Aymerich P, Vila-Gispert A, Guasch H (2022) Trophic competition in a guild of insectivorous semi-aquatic vertebrates in a Pyrenean headwater stream: diet specialisation in the endangered *Galemys pyrenaicus*. Mamm Biol 102(5–6):1673–1683

[28] Piggott MP (2004) Effect of sample age and season of collection on the reliability of microsatellite genotyping of faecal DNA. Wildl Res 31(5):485–493

[29] Santini A, Lucchini V, Fabbri E, Randi E (2007) Ageing and environmental factors affect PCR success in wolf (*Canis lupus*) excremental DNA samples. Mol Ecol Notes 7(6):955–961

[30] Miller SA, Dykes DD, Polesky HF (1988) A simple salting out procedure for extracting DNA from human nucleated cells. Nucleic Acids Res 16:1215 3344216 10.1093/nar/16.3.1215 PMC334765

[31] Untergasser A, Cutcutache I, Koressaar T, Ye J, Faircloth BC, RemmM RSG (2012) Primer3—new capabilities and interfaces. Nucleic Acids Res 40(15):e115–e115 22730293 10.1093/nar/gks596 PMC3424584

[32] Kumar S, Stecher G, Li M, Knyaz C (2018) Tamura K (2018) Molecular evolutionary genetics analysis across computing platforms. Mol Biol Evol 35(6):1547 29722887 10.1093/molbev/msy096 PMC5967553

[33] Bandelt HJ, Forster P, Röhl A (1999) Median-joining networks for inferring intraspecific phylogenies. Mol Biol Evol 16(1):37–48 10331250 10.1093/oxfordjournals.molbev.a026036

[34] Ruiz-González A, Madeira MJ, RandiE UF, Gómez-Moliner BJ (2013) Non-invasive genetic sampling of sympatric marten species (*Martes martes* and *Martes foina*): assessing species and individual identification success rates on faecal DNA genotyping. Eur J Wildl Res 59:371–386

[35] Zarzoso-Lacoste D, Corse E, Vidal E (2013) Improving PCR detection of prey in molecular diet studies: importance of group-specific primer set selection and extraction protocol performances. Mol Ecol Resour 13(1):117–127 23134438 10.1111/1755-0998.12029

[36] Latrofa MS, Weigl S, Dantas-Torres F, Annoscia G, Traversa D, Brianti E, Otranto D (2012) A multiplex PCR for the simultaneous detection of species of filarioids infesting dogs. Acta Trop 122(1):150–154 22248527 10.1016/j.actatropica.2012.01.006

[37] López-Calleja I, González I, Fajardo V, Martín I, Hernández PE, García T, Martín R (2007) Quantitative detection of goats’ milk in sheep’s milk by real-time PCR. Food Control 18(11):1466–1473

[38] Aulagnier S, Haffner P, Mitchell-Jones AJ, Moutou F, Zima J (2009) *Guía de los mamíferos de Europa, del norte de África y de Oriente Medio* p. 270. Bellaterra: Lynx.

[39] Gillet F, Cabria MT, Némoz M, Blanc F, Fournier-Chambrillon C, Sourp E et al (2015) PCR-RFLP identification of the endangered Pyrenean desman, Galemys pyrenaicus (Soricomorpha, Talpidae), based on faecal DNA. Mammalia 79(4):473–477

[40] Kierepka EM, Unger SD, Keiter DA, Beasley JC, Rhodes OE Jr, Cunningham FL, Piaggio AJ (2016) Identification of robust microsatellite markers for wild pig fecal DNA. J Wildl Manag 80:1120–1128

[41] O’Meara DB, Sheehy E, Turner PD, O’Mahony D, Harrington AP, Denman H et al (2014) Non-invasive multi-species monitoring: real-time PCR detection of small mammal and squirrel prey DNA in pine marten (Martes martes) scats. Acta Theriol 59:111–117

[42] Fernández-García JL, Carranza J, Martínez JG et al (2014) Mitochondrial D-loop phylogeny signals two native Iberian red deer (*Cervus elaphus*) Lineages genetically different to Western and Eastern European red deer and infers human-mediated translocations. Biodivers Conserv 23:537–554

[43] Templeton AR, Georgiadis NJ (1996) A landscape approach to conservation genetics: conserving evolutionary processes inthe African Bovidae In: Conservation Genetics: Case HistoriesFrom Nature(eds Avise JC, Hamrick JL) pp 398–430.

[44] Zachos FE, Cirovic D, Rottgardt I, Seiffert B, Oeking S, Eckert I, Hartl GB (2007) Geographically largescale genetic monomorphism in a highly successful introduced species: the case of the muskrat (*Ondatra zibethicus*) in Europe. Mamm Biol 72:123–126

