# Supplementary material for: Application of real-time PCR for the identification of the endangered species Galemys pyrenaicus through faecal samples
Source: Mol Biol Rep. 2024 Jan 5;51(1):76. doi: 10.1007/s11033-023-09010-2 (PMC10770224; doi:10.1007/s11033-023-09010-2)
Supplement: Supplementary file 1 — Supplementary file1 (DOC 2167 kb) [file 11033_2023_9010_MOESM1_ESM.doc]

Supplementary Material

| Table S1 a: In silico test for *G. pyrenaicus.*  *Number Hits:* Sequences producing Significant alignment (coverage (80-100) | | | |
| --- | --- | --- | --- |
| ***Taxonomy*** | **Number Hits** | **Number of Organisms** | **Percent identity** |
| [*Eukaryota*](https://www.ncbi.nlm.nih.gov/Taxonomy/Browser/wwwtax.cgi?id=2759) | [2825](https://blast.ncbi.nlm.nih.gov/Blast.cgi) | 214 |  |
| *.* [*Bilateria*](https://www.ncbi.nlm.nih.gov/Taxonomy/Browser/wwwtax.cgi?id=33213) | [2824](https://blast.ncbi.nlm.nih.gov/Blast.cgi) | 213 |  |
| *..* [*Euteleostomi*](https://www.ncbi.nlm.nih.gov/Taxonomy/Browser/wwwtax.cgi?id=117571) | [2572](https://blast.ncbi.nlm.nih.gov/Blast.cgi) | 121 |  |
| *...* [*Tetrapoda*](https://www.ncbi.nlm.nih.gov/Taxonomy/Browser/wwwtax.cgi?id=32523) | [1750](https://blast.ncbi.nlm.nih.gov/Blast.cgi) | 79 |  |
| *....* [*Amniota*](https://www.ncbi.nlm.nih.gov/Taxonomy/Browser/wwwtax.cgi?id=32524) | [1603](https://blast.ncbi.nlm.nih.gov/Blast.cgi) | 68 |  |
| *.....* [*Boreoeutheria*](https://www.ncbi.nlm.nih.gov/Taxonomy/Browser/wwwtax.cgi?id=1437010) | [1586](https://blast.ncbi.nlm.nih.gov/Blast.cgi) | 60 |  |
| *......* [*Laurasiatheria*](https://www.ncbi.nlm.nih.gov/Taxonomy/Browser/wwwtax.cgi?id=314145) | [1513](https://blast.ncbi.nlm.nih.gov/Blast.cgi) | 49 |  |
| *.......* [*Talpidae*](https://www.ncbi.nlm.nih.gov/Taxonomy/Browser/wwwtax.cgi?id=9373) | [1507](https://blast.ncbi.nlm.nih.gov/Blast.cgi) | 46 |  |
| *........* [*Galemys pyrenaicus*](https://www.ncbi.nlm.nih.gov/Taxonomy/Browser/wwwtax.cgi?id=202257) | [381](https://blast.ncbi.nlm.nih.gov/Blast.cgi) | 1 | [100](https://blast.ncbi.nlm.nih.gov/Blast.cgi) to 99.27 |
| *........* [*Talpa*](https://www.ncbi.nlm.nih.gov/Taxonomy/Browser/wwwtax.cgi?id=9374) | [680](https://blast.ncbi.nlm.nih.gov/Blast.cgi) | 14 | 90.27 to 91.97 |
| *......... Talpa europaea* | [246](https://blast.ncbi.nlm.nih.gov/Blast.cgi) | 1 |
| *......... Talpa stankovici* | [17](https://blast.ncbi.nlm.nih.gov/Blast.cgi) | 1 |
| *......... Talpa aquitania* | [127](https://blast.ncbi.nlm.nih.gov/Blast.cgi) | 1 |
| *......... Talpa levantis* | [46](https://blast.ncbi.nlm.nih.gov/Blast.cgi) | 1 |
| *......... Talpa caeca* | [9](https://blast.ncbi.nlm.nih.gov/Blast.cgi) | 1 |
| *......... unclassified Talpa* | [11](https://blast.ncbi.nlm.nih.gov/Blast.cgi) | 2 |
| *..........* [*Talpa sp. BA-2017*](https://www.ncbi.nlm.nih.gov/Taxonomy/Browser/wwwtax.cgi?id=1983367) | [8](https://blast.ncbi.nlm.nih.gov/Blast.cgi) | 1 |
| *..........* [*Talpa sp. AB-2015*](https://www.ncbi.nlm.nih.gov/Taxonomy/Browser/wwwtax.cgi?id=1607694) | [3](https://blast.ncbi.nlm.nih.gov/Blast.cgi) | 1 |
| *......... Talpa davidiana* | [6](https://blast.ncbi.nlm.nih.gov/Blast.cgi) | 1 |
| *......... Talpa caucasica* | [12](https://blast.ncbi.nlm.nih.gov/Blast.cgi) | 2 |
| *.......... Talpa caucasica ognevi* | [4](https://blast.ncbi.nlm.nih.gov/Blast.cgi) | 1 |
| *......... Talpa romana* | [120](https://blast.ncbi.nlm.nih.gov/Blast.cgi) | 1 |
| *......... Talpa occidentalis* | [53](https://blast.ncbi.nlm.nih.gov/Blast.cgi) | 1 |
| *......... Talpa martinorum* | [10](https://blast.ncbi.nlm.nih.gov/Blast.cgi) | 1 |
| *......... Talpa altaica* | [19](https://blast.ncbi.nlm.nih.gov/Blast.cgi) | 1 |
| *........* [*Neurotrichus*](https://www.ncbi.nlm.nih.gov/Taxonomy/Browser/wwwtax.cgi?id=182676) | [10](https://blast.ncbi.nlm.nih.gov/Blast.cgi) | 2 | < 91.97 |
| *.........* [*Neurotrichus gibbsii*](https://www.ncbi.nlm.nih.gov/Taxonomy/Browser/wwwtax.cgi?id=182677) | [9](https://blast.ncbi.nlm.nih.gov/Blast.cgi) | 2 |
| *..........* [*Neurotrichus gibbsii hyacinthinus*](https://www.ncbi.nlm.nih.gov/Taxonomy/Browser/wwwtax.cgi?id=2977538) | [1](https://blast.ncbi.nlm.nih.gov/Blast.cgi) | 1 |
| *........* [*Parascaptor leucura*](https://www.ncbi.nlm.nih.gov/Taxonomy/Browser/wwwtax.cgi?id=1300119) | [11](https://blast.ncbi.nlm.nih.gov/Blast.cgi) | 1 |
| *........* [*Mogera*](https://www.ncbi.nlm.nih.gov/Taxonomy/Browser/wwwtax.cgi?id=62294) | [308](https://blast.ncbi.nlm.nih.gov/Blast.cgi) | 9 |
| *.........* [*Mogera insularis*](https://www.ncbi.nlm.nih.gov/Taxonomy/Browser/wwwtax.cgi?id=114413) | [19](https://blast.ncbi.nlm.nih.gov/Blast.cgi) | 2 |
| *..........* [*Mogera insularis latouchei*](https://www.ncbi.nlm.nih.gov/Taxonomy/Browser/wwwtax.cgi?id=1340034) | [18](https://blast.ncbi.nlm.nih.gov/Blast.cgi) | 1 |
| *..........* [*Mogera insularis insularis*](https://www.ncbi.nlm.nih.gov/Taxonomy/Browser/wwwtax.cgi?id=2909291) | [1](https://blast.ncbi.nlm.nih.gov/Blast.cgi) | 1 |
| *.........* [*Mogera wogura*](https://www.ncbi.nlm.nih.gov/Taxonomy/Browser/wwwtax.cgi?id=62295) | [144](https://blast.ncbi.nlm.nih.gov/Blast.cgi) | 1 |
| *.........* [*Mogera robusta*](https://www.ncbi.nlm.nih.gov/Taxonomy/Browser/wwwtax.cgi?id=111428) | [106](https://blast.ncbi.nlm.nih.gov/Blast.cgi) | 1 |
| *.........* [*Mogera sp. FH-2015*](https://www.ncbi.nlm.nih.gov/Taxonomy/Browser/wwwtax.cgi?id=1752044) | [1](https://blast.ncbi.nlm.nih.gov/Blast.cgi) | 1 |
| *.........* [*Mogera imaizumii*](https://www.ncbi.nlm.nih.gov/Taxonomy/Browser/wwwtax.cgi?id=114415) | [30](https://blast.ncbi.nlm.nih.gov/Blast.cgi) | 1 |
| *.........* [*Mogera tokudae*](https://www.ncbi.nlm.nih.gov/Taxonomy/Browser/wwwtax.cgi?id=114414) | [3](https://blast.ncbi.nlm.nih.gov/Blast.cgi) | 1 |
| *.........* [*Mogera hainana*](https://www.ncbi.nlm.nih.gov/Taxonomy/Browser/wwwtax.cgi?id=2804713) | [3](https://blast.ncbi.nlm.nih.gov/Blast.cgi) | 1 |
| *.........* [*Mogera kanoana*](https://www.ncbi.nlm.nih.gov/Taxonomy/Browser/wwwtax.cgi?id=468912) | [2](https://blast.ncbi.nlm.nih.gov/Blast.cgi) | 1 |
| *........* [*Euroscaptor*](https://www.ncbi.nlm.nih.gov/Taxonomy/Browser/wwwtax.cgi?id=114410) | [82](https://blast.ncbi.nlm.nih.gov/Blast.cgi) | 14 |
| *.........* [*Euroscaptor kuznetsovi*](https://www.ncbi.nlm.nih.gov/Taxonomy/Browser/wwwtax.cgi?id=1955400) | [2](https://blast.ncbi.nlm.nih.gov/Blast.cgi) | 1 |
| *.........* [*Euroscaptor longirostris*](https://www.ncbi.nlm.nih.gov/Taxonomy/Browser/wwwtax.cgi?id=481574) | [16](https://blast.ncbi.nlm.nih.gov/Blast.cgi) | 1 |
| *......... Euroscaptor cf. orlovi* NTS.2019.04.56 | [1](https://blast.ncbi.nlm.nih.gov/Blast.cgi) | 1 |
| *......... Euroscaptor cf. orlovi* NTS.2019.04.50 | [1](https://blast.ncbi.nlm.nih.gov/Blast.cgi) | 1 |
| *......... Euroscaptor cf. orlovi* NTS.2019.04.48 | [1](https://blast.ncbi.nlm.nih.gov/Blast.cgi) | 1 |
| *......... Euroscaptor cf. orlovi* NTS.2019.04.38 | [1](https://blast.ncbi.nlm.nih.gov/Blast.cgi) | 1 |
| *......... Euroscaptor cf. orlovi* C.CHU.18.32 | [1](https://blast.ncbi.nlm.nih.gov/Blast.cgi) | 1 |
| *......... Euroscaptor cf. orlovi* C.CHU.18.31 | [1](https://blast.ncbi.nlm.nih.gov/Blast.cgi) | 1 |
| *.........* [*Euroscaptor klossi*](https://www.ncbi.nlm.nih.gov/Taxonomy/Browser/wwwtax.cgi?id=1340169) | [2](https://blast.ncbi.nlm.nih.gov/Blast.cgi) | 1 |
| *......... Euroscaptor cf. orlovi* NTS.2019.04.57 | [1](https://blast.ncbi.nlm.nih.gov/Blast.cgi) | 1 |
| *......... Euroscaptor parvidens* | [23](https://blast.ncbi.nlm.nih.gov/Blast.cgi) | 1 |
| *.........* [*Euroscaptor malayana*](https://www.ncbi.nlm.nih.gov/Taxonomy/Browser/wwwtax.cgi?id=533184) | [2](https://blast.ncbi.nlm.nih.gov/Blast.cgi) | 1 |
| *.........* [*Euroscaptor subanura*](https://www.ncbi.nlm.nih.gov/Taxonomy/Browser/wwwtax.cgi?id=1581555) | [29](https://blast.ncbi.nlm.nih.gov/Blast.cgi) | 1 |
| *......... Euroscaptor cf. orlovi* C.CHU.18.22 | [1](https://blast.ncbi.nlm.nih.gov/Blast.cgi) | 1 |
| *........* [*Alpiscaptulus sp. h CZ-2022*](https://www.ncbi.nlm.nih.gov/Taxonomy/Browser/wwwtax.cgi?id=2917339) | [2](https://blast.ncbi.nlm.nih.gov/Blast.cgi) | 1 |
| *........* [*Scaptonyx fusicaudus*](https://www.ncbi.nlm.nih.gov/Taxonomy/Browser/wwwtax.cgi?id=224955) | [14](https://blast.ncbi.nlm.nih.gov/Blast.cgi) | 1 |
| *........* [*Oreoscaptor mizura*](https://www.ncbi.nlm.nih.gov/Taxonomy/Browser/wwwtax.cgi?id=2835217) | [6](https://blast.ncbi.nlm.nih.gov/Blast.cgi) | 1 |
| *........* [*Scapanulus oweni*](https://www.ncbi.nlm.nih.gov/Taxonomy/Browser/wwwtax.cgi?id=1300117) | [11](https://blast.ncbi.nlm.nih.gov/Blast.cgi) | 1 |
| *........* [*Scaptochirus moschatus*](https://www.ncbi.nlm.nih.gov/Taxonomy/Browser/wwwtax.cgi?id=446353) | [2](https://blast.ncbi.nlm.nih.gov/Blast.cgi) | 1 |
| *.......* [*Microchiroptera*](https://www.ncbi.nlm.nih.gov/Taxonomy/Browser/wwwtax.cgi?id=30560) | [6](https://blast.ncbi.nlm.nih.gov/Blast.cgi) | 3 |
| *........* [*Phyllostomidae*](https://www.ncbi.nlm.nih.gov/Taxonomy/Browser/wwwtax.cgi?id=9415) | [5](https://blast.ncbi.nlm.nih.gov/Blast.cgi) | 2 |
| *.........* [*Phyllonycteris obtusa*](https://www.ncbi.nlm.nih.gov/Taxonomy/Browser/wwwtax.cgi?id=869459) | [4](https://blast.ncbi.nlm.nih.gov/Blast.cgi) | 1 |
| *.........* [*Micronycteris megalotis*](https://www.ncbi.nlm.nih.gov/Taxonomy/Browser/wwwtax.cgi?id=148066) | [1](https://blast.ncbi.nlm.nih.gov/Blast.cgi) | 1 |
| *........* [*Plecotus austriacus*](https://www.ncbi.nlm.nih.gov/Taxonomy/Browser/wwwtax.cgi?id=109483) | [1](https://blast.ncbi.nlm.nih.gov/Blast.cgi) | 1 |
| *......* [*Euarchontoglires*](https://www.ncbi.nlm.nih.gov/Taxonomy/Browser/wwwtax.cgi?id=314146) | [73](https://blast.ncbi.nlm.nih.gov/Blast.cgi) | 11 |
| *.......* [*Rodentia*](https://www.ncbi.nlm.nih.gov/Taxonomy/Browser/wwwtax.cgi?id=9989) | [67](https://blast.ncbi.nlm.nih.gov/Blast.cgi) | 10 |
| *........* [*Muroidea*](https://www.ncbi.nlm.nih.gov/Taxonomy/Browser/wwwtax.cgi?id=337687) | [14](https://blast.ncbi.nlm.nih.gov/Blast.cgi) | 5 |
| *.........* [*Murinae*](https://www.ncbi.nlm.nih.gov/Taxonomy/Browser/wwwtax.cgi?id=39107) | [11](https://blast.ncbi.nlm.nih.gov/Blast.cgi) | 4 |
| *..........* [*Pseudomys johnsoni*](https://www.ncbi.nlm.nih.gov/Taxonomy/Browser/wwwtax.cgi?id=442612) | [2](https://blast.ncbi.nlm.nih.gov/Blast.cgi) | 1 |
| *..........* [*Notomys*](https://www.ncbi.nlm.nih.gov/Taxonomy/Browser/wwwtax.cgi?id=184395) | [9](https://blast.ncbi.nlm.nih.gov/Blast.cgi) | 3 |
| *...........* [*Notomys aquilo*](https://www.ncbi.nlm.nih.gov/Taxonomy/Browser/wwwtax.cgi?id=442588) | [2](https://blast.ncbi.nlm.nih.gov/Blast.cgi) | 1 |
| *...........* [*Notomys mitchellii*](https://www.ncbi.nlm.nih.gov/Taxonomy/Browser/wwwtax.cgi?id=442590) | [2](https://blast.ncbi.nlm.nih.gov/Blast.cgi) | 1 |
| *...........* [*Notomys alexis*](https://www.ncbi.nlm.nih.gov/Taxonomy/Browser/wwwtax.cgi?id=184396) | [5](https://blast.ncbi.nlm.nih.gov/Blast.cgi) | 1 |
| *.........* [*Graomys griseoflavus*](https://www.ncbi.nlm.nih.gov/Taxonomy/Browser/wwwtax.cgi?id=118865) | [3](https://blast.ncbi.nlm.nih.gov/Blast.cgi) | 1 |
| *........* [*Hystricomorpha*](https://www.ncbi.nlm.nih.gov/Taxonomy/Browser/wwwtax.cgi?id=33550) | [50](https://blast.ncbi.nlm.nih.gov/Blast.cgi) | 3 |
| *.........* [*Fukomys foxi*](https://www.ncbi.nlm.nih.gov/Taxonomy/Browser/wwwtax.cgi?id=261004) | [1](https://blast.ncbi.nlm.nih.gov/Blast.cgi) | 1 |
| *.........* [*Cavia*](https://www.ncbi.nlm.nih.gov/Taxonomy/Browser/wwwtax.cgi?id=10140) | [49](https://blast.ncbi.nlm.nih.gov/Blast.cgi) | 2 |
| *..........* [*Cavia porcellus*](https://www.ncbi.nlm.nih.gov/Taxonomy/Browser/wwwtax.cgi?id=10141) | [48](https://blast.ncbi.nlm.nih.gov/Blast.cgi) | 1 |
| *..........* [*Cavia tschudii*](https://www.ncbi.nlm.nih.gov/Taxonomy/Browser/wwwtax.cgi?id=143287) | [1](https://blast.ncbi.nlm.nih.gov/Blast.cgi) | 1 |
| *........* [*unclassified Anomalurus*](https://www.ncbi.nlm.nih.gov/Taxonomy/Browser/wwwtax.cgi?id=2623273) | [3](https://blast.ncbi.nlm.nih.gov/Blast.cgi) | 2 |
| *.........* [*Anomalurus sp. GP-2005*](https://www.ncbi.nlm.nih.gov/Taxonomy/Browser/wwwtax.cgi?id=359030) | [2](https://blast.ncbi.nlm.nih.gov/Blast.cgi) | 1 |
| *.........* [*Anomalurus sp.*](https://www.ncbi.nlm.nih.gov/Taxonomy/Browser/wwwtax.cgi?id=101665) | [1](https://blast.ncbi.nlm.nih.gov/Blast.cgi) | 1 |
| *.......* [*Callithrix jacchus*](https://www.ncbi.nlm.nih.gov/Taxonomy/Browser/wwwtax.cgi?id=9483) | [6](https://blast.ncbi.nlm.nih.gov/Blast.cgi) | 1 |
| *.....* [*Sauria*](https://www.ncbi.nlm.nih.gov/Taxonomy/Browser/wwwtax.cgi?id=32561) | [17](https://blast.ncbi.nlm.nih.gov/Blast.cgi) | 8 |
| *......* [*Bifurcata*](https://www.ncbi.nlm.nih.gov/Taxonomy/Browser/wwwtax.cgi?id=1329961) | [12](https://blast.ncbi.nlm.nih.gov/Blast.cgi) | 7 |
| *.......* [*Unidentata*](https://www.ncbi.nlm.nih.gov/Taxonomy/Browser/wwwtax.cgi?id=1329950) | [8](https://blast.ncbi.nlm.nih.gov/Blast.cgi) | 4 |
| *........* [*Iguania*](https://www.ncbi.nlm.nih.gov/Taxonomy/Browser/wwwtax.cgi?id=8511) | [3](https://blast.ncbi.nlm.nih.gov/Blast.cgi) | 3 |
| *.........* [*Liolaemus*](https://www.ncbi.nlm.nih.gov/Taxonomy/Browser/wwwtax.cgi?id=43599) | [2](https://blast.ncbi.nlm.nih.gov/Blast.cgi) | 2 |
| *..........* [*Liolaemus cyanogaster*](https://www.ncbi.nlm.nih.gov/Taxonomy/Browser/wwwtax.cgi?id=109407) | [1](https://blast.ncbi.nlm.nih.gov/Blast.cgi) | 1 |
| *..........* [*Liolaemus cristiani*](https://www.ncbi.nlm.nih.gov/Taxonomy/Browser/wwwtax.cgi?id=2484728) | [1](https://blast.ncbi.nlm.nih.gov/Blast.cgi) | 1 |
| *.........* [*Sceloporus torquatus torquatus*](https://www.ncbi.nlm.nih.gov/Taxonomy/Browser/wwwtax.cgi?id=383828) | [1](https://blast.ncbi.nlm.nih.gov/Blast.cgi) | 1 |
| *........* [*Zonosaurus aeneus*](https://www.ncbi.nlm.nih.gov/Taxonomy/Browser/wwwtax.cgi?id=329865) | [5](https://blast.ncbi.nlm.nih.gov/Blast.cgi) | 1 |
| *.......* [*Gekkota*](https://www.ncbi.nlm.nih.gov/Taxonomy/Browser/wwwtax.cgi?id=8560) | [4](https://blast.ncbi.nlm.nih.gov/Blast.cgi) | 3 |
| *........* [*Gekkoninae*](https://www.ncbi.nlm.nih.gov/Taxonomy/Browser/wwwtax.cgi?id=385256) | [3](https://blast.ncbi.nlm.nih.gov/Blast.cgi) | 2 |
| *.........* [*Phelsuma lineata*](https://www.ncbi.nlm.nih.gov/Taxonomy/Browser/wwwtax.cgi?id=232296) | [2](https://blast.ncbi.nlm.nih.gov/Blast.cgi) | 1 |
| *.........* [*Gekko gecko*](https://www.ncbi.nlm.nih.gov/Taxonomy/Browser/wwwtax.cgi?id=36310) | [1](https://blast.ncbi.nlm.nih.gov/Blast.cgi) | 1 |
| *........* [*Goniurosaurus splendens*](https://www.ncbi.nlm.nih.gov/Taxonomy/Browser/wwwtax.cgi?id=1216930) | [1](https://blast.ncbi.nlm.nih.gov/Blast.cgi) | 1 |
| *......* [*Primolius maracana*](https://www.ncbi.nlm.nih.gov/Taxonomy/Browser/wwwtax.cgi?id=178894) | [5](https://blast.ncbi.nlm.nih.gov/Blast.cgi) | 1 |
| *....* [*Batrachia*](https://www.ncbi.nlm.nih.gov/Taxonomy/Browser/wwwtax.cgi?id=41666) | [147](https://blast.ncbi.nlm.nih.gov/Blast.cgi) | 11 |
| *.....* [*Salamandroidea*](https://www.ncbi.nlm.nih.gov/Taxonomy/Browser/wwwtax.cgi?id=30367) | [99](https://blast.ncbi.nlm.nih.gov/Blast.cgi) | 5 |
| *......* [*Ambystoma barbouri*](https://www.ncbi.nlm.nih.gov/Taxonomy/Browser/wwwtax.cgi?id=238860) | [27](https://blast.ncbi.nlm.nih.gov/Blast.cgi) | 1 |
| *......* [*Dicamptodon aterrimus*](https://www.ncbi.nlm.nih.gov/Taxonomy/Browser/wwwtax.cgi?id=294758) | [57](https://blast.ncbi.nlm.nih.gov/Blast.cgi) | 1 |
| *......* [*Rhyacotriton*](https://www.ncbi.nlm.nih.gov/Taxonomy/Browser/wwwtax.cgi?id=43568) | [6](https://blast.ncbi.nlm.nih.gov/Blast.cgi) | 2 |
| *.......* [*Rhyacotriton kezeri*](https://www.ncbi.nlm.nih.gov/Taxonomy/Browser/wwwtax.cgi?id=294362) | [1](https://blast.ncbi.nlm.nih.gov/Blast.cgi) | 1 |
| *.......* [*Rhyacotriton cascadae*](https://www.ncbi.nlm.nih.gov/Taxonomy/Browser/wwwtax.cgi?id=307116) | [5](https://blast.ncbi.nlm.nih.gov/Blast.cgi) | 1 |
| *......* [*Bolitoglossa oresbia*](https://www.ncbi.nlm.nih.gov/Taxonomy/Browser/wwwtax.cgi?id=1483524) | [9](https://blast.ncbi.nlm.nih.gov/Blast.cgi) | 1 |
| *.....* [*Anura*](https://www.ncbi.nlm.nih.gov/Taxonomy/Browser/wwwtax.cgi?id=8342) | [48](https://blast.ncbi.nlm.nih.gov/Blast.cgi) | 6 |
| *......* [*Microhyloidea*](https://www.ncbi.nlm.nih.gov/Taxonomy/Browser/wwwtax.cgi?id=8426) | [4](https://blast.ncbi.nlm.nih.gov/Blast.cgi) | 3 |
| *.......* [*Microhylidae*](https://www.ncbi.nlm.nih.gov/Taxonomy/Browser/wwwtax.cgi?id=8427) | [3](https://blast.ncbi.nlm.nih.gov/Blast.cgi) | 2 |
| *........* [*Microhyla butleri*](https://www.ncbi.nlm.nih.gov/Taxonomy/Browser/wwwtax.cgi?id=161703) | [2](https://blast.ncbi.nlm.nih.gov/Blast.cgi) | 1 |
| *........ Otophryne sp.* AMNH 101748 | [1](https://blast.ncbi.nlm.nih.gov/Blast.cgi) | 1 |
| *.......* [*Hyperolius semidiscus*](https://www.ncbi.nlm.nih.gov/Taxonomy/Browser/wwwtax.cgi?id=143419) | [1](https://blast.ncbi.nlm.nih.gov/Blast.cgi) | 1 |
| *......* [*Bombina*](https://www.ncbi.nlm.nih.gov/Taxonomy/Browser/wwwtax.cgi?id=8344) | [44](https://blast.ncbi.nlm.nih.gov/Blast.cgi) | 3 |
| *.......* [*Bombina maxima*](https://www.ncbi.nlm.nih.gov/Taxonomy/Browser/wwwtax.cgi?id=161274) | [32](https://blast.ncbi.nlm.nih.gov/Blast.cgi) | 1 |
| *.......* [*Bombina microdeladigitora*](https://www.ncbi.nlm.nih.gov/Taxonomy/Browser/wwwtax.cgi?id=356193) | [11](https://blast.ncbi.nlm.nih.gov/Blast.cgi) | 1 |
| *.......* [*Bombina fortinuptialis*](https://www.ncbi.nlm.nih.gov/Taxonomy/Browser/wwwtax.cgi?id=414351) | [1](https://blast.ncbi.nlm.nih.gov/Blast.cgi) | 1 |
| *...* [*Clupeocephala*](https://www.ncbi.nlm.nih.gov/Taxonomy/Browser/wwwtax.cgi?id=186625) | [822](https://blast.ncbi.nlm.nih.gov/Blast.cgi) | 42 |
| *....* [*Otomorpha*](https://www.ncbi.nlm.nih.gov/Taxonomy/Browser/wwwtax.cgi?id=186634) | [724](https://blast.ncbi.nlm.nih.gov/Blast.cgi) | 36 |
| *.....* [*Characiphysae*](https://www.ncbi.nlm.nih.gov/Taxonomy/Browser/wwwtax.cgi?id=186628) | [722](https://blast.ncbi.nlm.nih.gov/Blast.cgi) | 35 |
| *......* [*Siluroidei*](https://www.ncbi.nlm.nih.gov/Taxonomy/Browser/wwwtax.cgi?id=1489793) | [363](https://blast.ncbi.nlm.nih.gov/Blast.cgi) | 14 |
| *.......* [*Amphilius*](https://www.ncbi.nlm.nih.gov/Taxonomy/Browser/wwwtax.cgi?id=390476) | [4](https://blast.ncbi.nlm.nih.gov/Blast.cgi) | 2 |
| *........* [*Amphilius atesuensis*](https://www.ncbi.nlm.nih.gov/Taxonomy/Browser/wwwtax.cgi?id=691900) | [1](https://blast.ncbi.nlm.nih.gov/Blast.cgi) | 1 |
| *........* [*Amphilius cf. rheophilus 'St. Paul'*](https://www.ncbi.nlm.nih.gov/Taxonomy/Browser/wwwtax.cgi?id=1494485) | [3](https://blast.ncbi.nlm.nih.gov/Blast.cgi) | 1 |
| *....... Schilbe cf. yangambianus* FDBS-2022 | [1](https://blast.ncbi.nlm.nih.gov/Blast.cgi) | 1 |
| *.......* [*Pimelodella sp. JV-2007*](https://www.ncbi.nlm.nih.gov/Taxonomy/Browser/wwwtax.cgi?id=462336) | [1](https://blast.ncbi.nlm.nih.gov/Blast.cgi) | 1 |
| *.......* [*Rhamdia*](https://www.ncbi.nlm.nih.gov/Taxonomy/Browser/wwwtax.cgi?id=55672) | [353](https://blast.ncbi.nlm.nih.gov/Blast.cgi) | 7 |
| *........* [*Rhamdia quelen*](https://www.ncbi.nlm.nih.gov/Taxonomy/Browser/wwwtax.cgi?id=162147) | [282](https://blast.ncbi.nlm.nih.gov/Blast.cgi) | 1 |
| *........* [*Rhamdia aff. quelen CC1*](https://www.ncbi.nlm.nih.gov/Taxonomy/Browser/wwwtax.cgi?id=2748217) | [10](https://blast.ncbi.nlm.nih.gov/Blast.cgi) | 1 |
| *........* [*Rhamdia aff. quelen CC2*](https://www.ncbi.nlm.nih.gov/Taxonomy/Browser/wwwtax.cgi?id=2748218) | [10](https://blast.ncbi.nlm.nih.gov/Blast.cgi) | 1 |
| *........* [*Rhamdia aff. quelen CC3*](https://www.ncbi.nlm.nih.gov/Taxonomy/Browser/wwwtax.cgi?id=2748219) | [27](https://blast.ncbi.nlm.nih.gov/Blast.cgi) | 1 |
| *........* [*Rhamdia aff. quelen LA4*](https://www.ncbi.nlm.nih.gov/Taxonomy/Browser/wwwtax.cgi?id=2748226) | [3](https://blast.ncbi.nlm.nih.gov/Blast.cgi) | 1 |
| *........* [*Rhamdia sapo*](https://www.ncbi.nlm.nih.gov/Taxonomy/Browser/wwwtax.cgi?id=55673) | [20](https://blast.ncbi.nlm.nih.gov/Blast.cgi) | 1 |
| *........* [*Rhamdia aff. quelen HA1*](https://www.ncbi.nlm.nih.gov/Taxonomy/Browser/wwwtax.cgi?id=2748222) | [1](https://blast.ncbi.nlm.nih.gov/Blast.cgi) | 1 |
| *.......* [*Nanobagrus*](https://www.ncbi.nlm.nih.gov/Taxonomy/Browser/wwwtax.cgi?id=337727) | [3](https://blast.ncbi.nlm.nih.gov/Blast.cgi) | 2 |
| *........* [*Nanobagrus fuscus*](https://www.ncbi.nlm.nih.gov/Taxonomy/Browser/wwwtax.cgi?id=337734) | [1](https://blast.ncbi.nlm.nih.gov/Blast.cgi) | 1 |
| *........* [*Nanobagrus torquatus*](https://www.ncbi.nlm.nih.gov/Taxonomy/Browser/wwwtax.cgi?id=531821) | [2](https://blast.ncbi.nlm.nih.gov/Blast.cgi) | 1 |
| *.......* [*Hemicetopsis candiru*](https://www.ncbi.nlm.nih.gov/Taxonomy/Browser/wwwtax.cgi?id=337683) | [1](https://blast.ncbi.nlm.nih.gov/Blast.cgi) | 1 |
| *......* [*Characiformes*](https://www.ncbi.nlm.nih.gov/Taxonomy/Browser/wwwtax.cgi?id=7991) | [359](https://blast.ncbi.nlm.nih.gov/Blast.cgi) | 21 |
| *.......* [*Nannaethiops gracilis*](https://www.ncbi.nlm.nih.gov/Taxonomy/Browser/wwwtax.cgi?id=1387203) | [2](https://blast.ncbi.nlm.nih.gov/Blast.cgi) | 1 |
| *.......* [*Characidae*](https://www.ncbi.nlm.nih.gov/Taxonomy/Browser/wwwtax.cgi?id=7992) | [357](https://blast.ncbi.nlm.nih.gov/Blast.cgi) | 20 |
| *........* [*Hyphessobrycon socolofi*](https://www.ncbi.nlm.nih.gov/Taxonomy/Browser/wwwtax.cgi?id=2979627) | [2](https://blast.ncbi.nlm.nih.gov/Blast.cgi) | 1 |
| *........* [*Stethaprioninae*](https://www.ncbi.nlm.nih.gov/Taxonomy/Browser/wwwtax.cgi?id=42595) | [354](https://blast.ncbi.nlm.nih.gov/Blast.cgi) | 18 |
| *.........* [*Astyanax*](https://www.ncbi.nlm.nih.gov/Taxonomy/Browser/wwwtax.cgi?id=7993) | [277](https://blast.ncbi.nlm.nih.gov/Blast.cgi) | 17 |
| *..........* [*Astyanax hubbsi*](https://www.ncbi.nlm.nih.gov/Taxonomy/Browser/wwwtax.cgi?id=643503) | [3](https://blast.ncbi.nlm.nih.gov/Blast.cgi) | 1 |
| *..........* [*Astyanax aeneus*](https://www.ncbi.nlm.nih.gov/Taxonomy/Browser/wwwtax.cgi?id=178766) | [135](https://blast.ncbi.nlm.nih.gov/Blast.cgi) | 1 |
| *..........* [*Astyanax orthodus*](https://www.ncbi.nlm.nih.gov/Taxonomy/Browser/wwwtax.cgi?id=643506) | [1](https://blast.ncbi.nlm.nih.gov/Blast.cgi) | 1 |
| *..........* [*unclassified Astyanax*](https://www.ncbi.nlm.nih.gov/Taxonomy/Browser/wwwtax.cgi?id=2602611) | [24](https://blast.ncbi.nlm.nih.gov/Blast.cgi) | 6 |
| *........... Astyanax sp.* 6 CPOG-2009 | [4](https://blast.ncbi.nlm.nih.gov/Blast.cgi) | 1 |
| *........... Astyanax sp.* 2 CPOG-2009 | [4](https://blast.ncbi.nlm.nih.gov/Blast.cgi) | 1 |
| *........... Astyanax sp. 9* CPOG-2009 | [3](https://blast.ncbi.nlm.nih.gov/Blast.cgi) | 1 |
| *........... Astyanax sp.* 3 CPOG-2009 | [1](https://blast.ncbi.nlm.nih.gov/Blast.cgi) | 1 |
| *........... Astyanax sp.* 5 CPOG-2009 | [1](https://blast.ncbi.nlm.nih.gov/Blast.cgi) | 1 |
| *........... Astyanax sp*. 1 CPOG-2009 | [11](https://blast.ncbi.nlm.nih.gov/Blast.cgi) | 1 |
| *..........* [*Astyanax belizanus*](https://www.ncbi.nlm.nih.gov/Taxonomy/Browser/wwwtax.cgi?id=643502) | [8](https://blast.ncbi.nlm.nih.gov/Blast.cgi) | 1 |
| *..........* [*Astyanax nicaraguensis*](https://www.ncbi.nlm.nih.gov/Taxonomy/Browser/wwwtax.cgi?id=643505) | [44](https://blast.ncbi.nlm.nih.gov/Blast.cgi) | 1 |
| *..........* [*Astyanax bransfordii*](https://www.ncbi.nlm.nih.gov/Taxonomy/Browser/wwwtax.cgi?id=2816895) | [7](https://blast.ncbi.nlm.nih.gov/Blast.cgi) | 1 |
| *..........* [*Astyanax nasutus*](https://www.ncbi.nlm.nih.gov/Taxonomy/Browser/wwwtax.cgi?id=643504) | [16](https://blast.ncbi.nlm.nih.gov/Blast.cgi) | 1 |
| *..........* [*Astyanax caballeroi*](https://www.ncbi.nlm.nih.gov/Taxonomy/Browser/wwwtax.cgi?id=533334) | [1](https://blast.ncbi.nlm.nih.gov/Blast.cgi) | 1 |
| *..........* [*Astyanax petenensis*](https://www.ncbi.nlm.nih.gov/Taxonomy/Browser/wwwtax.cgi?id=643507) | [3](https://blast.ncbi.nlm.nih.gov/Blast.cgi) | 1 |
| *..........* [*Astyanax mexicanus*](https://www.ncbi.nlm.nih.gov/Taxonomy/Browser/wwwtax.cgi?id=7994) | [34](https://blast.ncbi.nlm.nih.gov/Blast.cgi) | 1 |
| *..........* [*Astyanax jordani*](https://www.ncbi.nlm.nih.gov/Taxonomy/Browser/wwwtax.cgi?id=930233) | [1](https://blast.ncbi.nlm.nih.gov/Blast.cgi) | 1 |
| *.........* [*Psalidodon fasciatus*](https://www.ncbi.nlm.nih.gov/Taxonomy/Browser/wwwtax.cgi?id=223369) | [77](https://blast.ncbi.nlm.nih.gov/Blast.cgi) | 1 |
| *........* [*Bramocharax baileyi*](https://www.ncbi.nlm.nih.gov/Taxonomy/Browser/wwwtax.cgi?id=533333) | [1](https://blast.ncbi.nlm.nih.gov/Blast.cgi) | 1 |
| *.....* [*Denticeps clupeoides*](https://www.ncbi.nlm.nih.gov/Taxonomy/Browser/wwwtax.cgi?id=299321) | [2](https://blast.ncbi.nlm.nih.gov/Blast.cgi) | 1 |
| *....* [*Percomorphaceae*](https://www.ncbi.nlm.nih.gov/Taxonomy/Browser/wwwtax.cgi?id=1489872) | [98](https://blast.ncbi.nlm.nih.gov/Blast.cgi) | 6 |
| *.....* [*Eupercaria*](https://www.ncbi.nlm.nih.gov/Taxonomy/Browser/wwwtax.cgi?id=1489922) | [17](https://blast.ncbi.nlm.nih.gov/Blast.cgi) | 3 |
| *......* [*Micropterus*](https://www.ncbi.nlm.nih.gov/Taxonomy/Browser/wwwtax.cgi?id=27705) | [5](https://blast.ncbi.nlm.nih.gov/Blast.cgi) | 2 |
| *.......* [*Micropterus punctulatus*](https://www.ncbi.nlm.nih.gov/Taxonomy/Browser/wwwtax.cgi?id=225060) | [2](https://blast.ncbi.nlm.nih.gov/Blast.cgi) | 1 |
| *.......* [*Micropterus treculii*](https://www.ncbi.nlm.nih.gov/Taxonomy/Browser/wwwtax.cgi?id=225392) | [3](https://blast.ncbi.nlm.nih.gov/Blast.cgi) | 1 |
| *......* [*Lophius vomerinus*](https://www.ncbi.nlm.nih.gov/Taxonomy/Browser/wwwtax.cgi?id=549588) | [12](https://blast.ncbi.nlm.nih.gov/Blast.cgi) | 1 |
| *.....* [*Gobionellinae*](https://www.ncbi.nlm.nih.gov/Taxonomy/Browser/wwwtax.cgi?id=497220) | [79](https://blast.ncbi.nlm.nih.gov/Blast.cgi) | 2 |
| *......* [*Tridentiger barbatus*](https://www.ncbi.nlm.nih.gov/Taxonomy/Browser/wwwtax.cgi?id=88147) | [74](https://blast.ncbi.nlm.nih.gov/Blast.cgi) | 1 |
| *......* [*Mugilogobius abei*](https://www.ncbi.nlm.nih.gov/Taxonomy/Browser/wwwtax.cgi?id=88200) | [5](https://blast.ncbi.nlm.nih.gov/Blast.cgi) | 1 |
| *.....* [*Foetorepus altivelis*](https://www.ncbi.nlm.nih.gov/Taxonomy/Browser/wwwtax.cgi?id=215388) | [2](https://blast.ncbi.nlm.nih.gov/Blast.cgi) | 1 |
| *..* [*Protostomia*](https://www.ncbi.nlm.nih.gov/Taxonomy/Browser/wwwtax.cgi?id=33317) | [252](https://blast.ncbi.nlm.nih.gov/Blast.cgi) | 92 |
| *...* [*Arthropoda*](https://www.ncbi.nlm.nih.gov/Taxonomy/Browser/wwwtax.cgi?id=6656) | [244](https://blast.ncbi.nlm.nih.gov/Blast.cgi) | 88 |
| *....* [*Pancrustacea*](https://www.ncbi.nlm.nih.gov/Taxonomy/Browser/wwwtax.cgi?id=197562) | [239](https://blast.ncbi.nlm.nih.gov/Blast.cgi) | 84 |
| *.....* [*Pterygota*](https://www.ncbi.nlm.nih.gov/Taxonomy/Browser/wwwtax.cgi?id=7496) | [231](https://blast.ncbi.nlm.nih.gov/Blast.cgi) | 81 |
| *......* [*Neoptera*](https://www.ncbi.nlm.nih.gov/Taxonomy/Browser/wwwtax.cgi?id=33340) | [229](https://blast.ncbi.nlm.nih.gov/Blast.cgi) | 80 |
| *.......* [*Polyneoptera*](https://www.ncbi.nlm.nih.gov/Taxonomy/Browser/wwwtax.cgi?id=33341) | [114](https://blast.ncbi.nlm.nih.gov/Blast.cgi) | 34 |
| *........* [*Orthoptera*](https://www.ncbi.nlm.nih.gov/Taxonomy/Browser/wwwtax.cgi?id=6993) | [106](https://blast.ncbi.nlm.nih.gov/Blast.cgi) | 28 |
| *.........* [*Acridomorpha*](https://www.ncbi.nlm.nih.gov/Taxonomy/Browser/wwwtax.cgi?id=70910) | [42](https://blast.ncbi.nlm.nih.gov/Blast.cgi) | 23 |
| *..........* [*Acridoidea*](https://www.ncbi.nlm.nih.gov/Taxonomy/Browser/wwwtax.cgi?id=92621) | [32](https://blast.ncbi.nlm.nih.gov/Blast.cgi) | 17 |
| *...........* [*Acrididae*](https://www.ncbi.nlm.nih.gov/Taxonomy/Browser/wwwtax.cgi?id=7002) | [29](https://blast.ncbi.nlm.nih.gov/Blast.cgi) | 15 |
| *............* [*Catantopinae*](https://www.ncbi.nlm.nih.gov/Taxonomy/Browser/wwwtax.cgi?id=109883) | [20](https://blast.ncbi.nlm.nih.gov/Blast.cgi) | 8 |
| *.............* [*Ranacris albicornis*](https://www.ncbi.nlm.nih.gov/Taxonomy/Browser/wwwtax.cgi?id=3016386) | [2](https://blast.ncbi.nlm.nih.gov/Blast.cgi) | 1 |
| *.............* [*Diabolocatantops pinguis*](https://www.ncbi.nlm.nih.gov/Taxonomy/Browser/wwwtax.cgi?id=509737) | [3](https://blast.ncbi.nlm.nih.gov/Blast.cgi) | 1 |
| *.............* [*Xenocatantops*](https://www.ncbi.nlm.nih.gov/Taxonomy/Browser/wwwtax.cgi?id=227618) | [6](https://blast.ncbi.nlm.nih.gov/Blast.cgi) | 2 |
| *..............* [*Xenocatantops brachycerus*](https://www.ncbi.nlm.nih.gov/Taxonomy/Browser/wwwtax.cgi?id=227619) | [4](https://blast.ncbi.nlm.nih.gov/Blast.cgi) | 1 |
| *..............* [*Xenocatantops humilis*](https://www.ncbi.nlm.nih.gov/Taxonomy/Browser/wwwtax.cgi?id=227620) | [2](https://blast.ncbi.nlm.nih.gov/Blast.cgi) | 1 |
| *.............* [*Stenocatantops*](https://www.ncbi.nlm.nih.gov/Taxonomy/Browser/wwwtax.cgi?id=227616) | [7](https://blast.ncbi.nlm.nih.gov/Blast.cgi) | 2 |
| *..............* [*Stenocatantops splendens*](https://www.ncbi.nlm.nih.gov/Taxonomy/Browser/wwwtax.cgi?id=227617) | [4](https://blast.ncbi.nlm.nih.gov/Blast.cgi) | 1 |
| *..............* [*Stenocatantops mistshenkoi*](https://www.ncbi.nlm.nih.gov/Taxonomy/Browser/wwwtax.cgi?id=2767328) | [3](https://blast.ncbi.nlm.nih.gov/Blast.cgi) | 1 |
| *.............* [*Goniaea vocans*](https://www.ncbi.nlm.nih.gov/Taxonomy/Browser/wwwtax.cgi?id=1290259) | [1](https://blast.ncbi.nlm.nih.gov/Blast.cgi) | 1 |
| *............. Catantops sp.* 1 GFJ-2008 | [1](https://blast.ncbi.nlm.nih.gov/Blast.cgi) | 1 |
| *............* [*Caryanda elegans*](https://www.ncbi.nlm.nih.gov/Taxonomy/Browser/wwwtax.cgi?id=316080) | [2](https://blast.ncbi.nlm.nih.gov/Blast.cgi) | 1 |
| *............* [*Pacris xizangensis*](https://www.ncbi.nlm.nih.gov/Taxonomy/Browser/wwwtax.cgi?id=997599) | [2](https://blast.ncbi.nlm.nih.gov/Blast.cgi) | 1 |
| *............ Gomphocerinae* | [5](https://blast.ncbi.nlm.nih.gov/Blast.cgi) | 5 |
| *.............* [*Paropomala wyomingensis*](https://www.ncbi.nlm.nih.gov/Taxonomy/Browser/wwwtax.cgi?id=1034408) | [1](https://blast.ncbi.nlm.nih.gov/Blast.cgi) | 1 |
| *.............* [*Opeia obscura*](https://www.ncbi.nlm.nih.gov/Taxonomy/Browser/wwwtax.cgi?id=1034392) | [1](https://blast.ncbi.nlm.nih.gov/Blast.cgi) | 1 |
| *.............* [*Psoloessa delicatula*](https://www.ncbi.nlm.nih.gov/Taxonomy/Browser/wwwtax.cgi?id=1034428) | [1](https://blast.ncbi.nlm.nih.gov/Blast.cgi) | 1 |
| *.............* [*Eritettix simplex*](https://www.ncbi.nlm.nih.gov/Taxonomy/Browser/wwwtax.cgi?id=1034426) | [1](https://blast.ncbi.nlm.nih.gov/Blast.cgi) | 1 |
| *.............* [*Phlaeoba infumata*](https://www.ncbi.nlm.nih.gov/Taxonomy/Browser/wwwtax.cgi?id=227577) | [1](https://blast.ncbi.nlm.nih.gov/Blast.cgi) | 1 |
| *...........* [*Romaleinae*](https://www.ncbi.nlm.nih.gov/Taxonomy/Browser/wwwtax.cgi?id=2060512) | [3](https://blast.ncbi.nlm.nih.gov/Blast.cgi) | 2 |
| *............* [*Romalea microptera*](https://www.ncbi.nlm.nih.gov/Taxonomy/Browser/wwwtax.cgi?id=7007) | [2](https://blast.ncbi.nlm.nih.gov/Blast.cgi) | 1 |
| *............* [*Taeniopoda tamaluipensis*](https://www.ncbi.nlm.nih.gov/Taxonomy/Browser/wwwtax.cgi?id=92620) | [1](https://blast.ncbi.nlm.nih.gov/Blast.cgi) | 1 |
| *..........* [*Pyrgomorphidae*](https://www.ncbi.nlm.nih.gov/Taxonomy/Browser/wwwtax.cgi?id=58543) | [10](https://blast.ncbi.nlm.nih.gov/Blast.cgi) | 6 |
| *...........* [*Mekongiella*](https://www.ncbi.nlm.nih.gov/Taxonomy/Browser/wwwtax.cgi?id=244709) | [5](https://blast.ncbi.nlm.nih.gov/Blast.cgi) | 3 |
| *............* [*Mekongiella kingdoni*](https://www.ncbi.nlm.nih.gov/Taxonomy/Browser/wwwtax.cgi?id=244710) | [2](https://blast.ncbi.nlm.nih.gov/Blast.cgi) | 1 |
| *............* [*Mekongiella wardi*](https://www.ncbi.nlm.nih.gov/Taxonomy/Browser/wwwtax.cgi?id=1570007) | [1](https://blast.ncbi.nlm.nih.gov/Blast.cgi) | 1 |
| *............* [*Mekongiella xizangensis*](https://www.ncbi.nlm.nih.gov/Taxonomy/Browser/wwwtax.cgi?id=868578) | [2](https://blast.ncbi.nlm.nih.gov/Blast.cgi) | 1 |
| *...........* [*Zonocerus elegans*](https://www.ncbi.nlm.nih.gov/Taxonomy/Browser/wwwtax.cgi?id=58579) | [1](https://blast.ncbi.nlm.nih.gov/Blast.cgi) | 1 |
| *...........* [*Tagasta*](https://www.ncbi.nlm.nih.gov/Taxonomy/Browser/wwwtax.cgi?id=281094) | [4](https://blast.ncbi.nlm.nih.gov/Blast.cgi) | 2 |
| *............* [*Tagasta indica*](https://www.ncbi.nlm.nih.gov/Taxonomy/Browser/wwwtax.cgi?id=281095) | [2](https://blast.ncbi.nlm.nih.gov/Blast.cgi) | 1 |
| *............* [*Tagasta tonkinensis*](https://www.ncbi.nlm.nih.gov/Taxonomy/Browser/wwwtax.cgi?id=316081) | [2](https://blast.ncbi.nlm.nih.gov/Blast.cgi) | 1 |
| *.........* [*Ensifera*](https://www.ncbi.nlm.nih.gov/Taxonomy/Browser/wwwtax.cgi?id=6994) | [64](https://blast.ncbi.nlm.nih.gov/Blast.cgi) | 5 |
| *.......... Myrmecophilus* | [63](https://blast.ncbi.nlm.nih.gov/Blast.cgi) | 4 |
| *...........* [*Myrmecophilus hebardi*](https://www.ncbi.nlm.nih.gov/Taxonomy/Browser/wwwtax.cgi?id=2732521) | [32](https://blast.ncbi.nlm.nih.gov/Blast.cgi) | 1 |
| *...........* [*unclassified Myrmecophilus*](https://www.ncbi.nlm.nih.gov/Taxonomy/Browser/wwwtax.cgi?id=2637704) | [2](https://blast.ncbi.nlm.nih.gov/Blast.cgi) | 2 |
| *............ Myrmecophilus sp.* PheNPLC1 | [1](https://blast.ncbi.nlm.nih.gov/Blast.cgi) | 1 |
| *............* [*Myrmecophilus sp. palM*](https://www.ncbi.nlm.nih.gov/Taxonomy/Browser/wwwtax.cgi?id=2733850) | [1](https://blast.ncbi.nlm.nih.gov/Blast.cgi) | 1 |
| *...........* [*Myrmecophilus dubius*](https://www.ncbi.nlm.nih.gov/Taxonomy/Browser/wwwtax.cgi?id=2732520) | [29](https://blast.ncbi.nlm.nih.gov/Blast.cgi) | 1 |
| *..........* [*Diestrammena sp.*](https://www.ncbi.nlm.nih.gov/Taxonomy/Browser/wwwtax.cgi?id=2844945) | [1](https://blast.ncbi.nlm.nih.gov/Blast.cgi) | 1 |
| *........* [*Blattodea*](https://www.ncbi.nlm.nih.gov/Taxonomy/Browser/wwwtax.cgi?id=85823) | [6](https://blast.ncbi.nlm.nih.gov/Blast.cgi) | 5 |
| *.........* [*Blattoidea*](https://www.ncbi.nlm.nih.gov/Taxonomy/Browser/wwwtax.cgi?id=1049657) | [5](https://blast.ncbi.nlm.nih.gov/Blast.cgi) | 4 |
| *..........* [*Termitoidae*](https://www.ncbi.nlm.nih.gov/Taxonomy/Browser/wwwtax.cgi?id=1912919) | [4](https://blast.ncbi.nlm.nih.gov/Blast.cgi) | 3 |
| *...........* [*Parrhinotermes aequalis*](https://www.ncbi.nlm.nih.gov/Taxonomy/Browser/wwwtax.cgi?id=127380) | [1](https://blast.ncbi.nlm.nih.gov/Blast.cgi) | 1 |
| *...........* [*Termitidae*](https://www.ncbi.nlm.nih.gov/Taxonomy/Browser/wwwtax.cgi?id=46569) | [3](https://blast.ncbi.nlm.nih.gov/Blast.cgi) | 2 |
| *............ Diversitermes sp.* A TB-2017 | [1](https://blast.ncbi.nlm.nih.gov/Blast.cgi) | 1 |
| *............* [*Tuberculitermes bycanistes*](https://www.ncbi.nlm.nih.gov/Taxonomy/Browser/wwwtax.cgi?id=377997) | [2](https://blast.ncbi.nlm.nih.gov/Blast.cgi) | 1 |
| *..........* [*Neostylopyga rhombifolia*](https://www.ncbi.nlm.nih.gov/Taxonomy/Browser/wwwtax.cgi?id=304879) | [1](https://blast.ncbi.nlm.nih.gov/Blast.cgi) | 1 |
| *.........* [*Episymploce torchaceus*](https://www.ncbi.nlm.nih.gov/Taxonomy/Browser/wwwtax.cgi?id=1928779) | [1](https://blast.ncbi.nlm.nih.gov/Blast.cgi) | 1 |
| *........* [*Sosibia ovata*](https://www.ncbi.nlm.nih.gov/Taxonomy/Browser/wwwtax.cgi?id=3026509) | [2](https://blast.ncbi.nlm.nih.gov/Blast.cgi) | 1 |
| *.......* [*Endopterygota*](https://www.ncbi.nlm.nih.gov/Taxonomy/Browser/wwwtax.cgi?id=33392) | [93](https://blast.ncbi.nlm.nih.gov/Blast.cgi) | 31 |
| *........* [*Coleoptera*](https://www.ncbi.nlm.nih.gov/Taxonomy/Browser/wwwtax.cgi?id=7041) | [12](https://blast.ncbi.nlm.nih.gov/Blast.cgi) | 9 |
| *.........* [*Polyphaga*](https://www.ncbi.nlm.nih.gov/Taxonomy/Browser/wwwtax.cgi?id=41084) | [10](https://blast.ncbi.nlm.nih.gov/Blast.cgi) | 8 |
| *..........* [*Cucujiformia*](https://www.ncbi.nlm.nih.gov/Taxonomy/Browser/wwwtax.cgi?id=41088) | [4](https://blast.ncbi.nlm.nih.gov/Blast.cgi) | 4 |
| *........... Alleculinae sp.* ENSP01 | [1](https://blast.ncbi.nlm.nih.gov/Blast.cgi) | 1 |
| *........... Propylea sp.* HSL-2016 | [1](https://blast.ncbi.nlm.nih.gov/Blast.cgi) | 1 |
| *...........* [*Chrysomelidae*](https://www.ncbi.nlm.nih.gov/Taxonomy/Browser/wwwtax.cgi?id=27439) | [2](https://blast.ncbi.nlm.nih.gov/Blast.cgi) | 2 |
| *............ Neolema sp.* EMHAU-15071012 | [1](https://blast.ncbi.nlm.nih.gov/Blast.cgi) | 1 |
| *............* [*Gallerucida bifasciata*](https://www.ncbi.nlm.nih.gov/Taxonomy/Browser/wwwtax.cgi?id=301066) | [1](https://blast.ncbi.nlm.nih.gov/Blast.cgi) | 1 |
| *..........* [*Staphylinidae*](https://www.ncbi.nlm.nih.gov/Taxonomy/Browser/wwwtax.cgi?id=29026) | [3](https://blast.ncbi.nlm.nih.gov/Blast.cgi) | 2 |
| *...........* [*Ocypus olens*](https://www.ncbi.nlm.nih.gov/Taxonomy/Browser/wwwtax.cgi?id=662956) | [2](https://blast.ncbi.nlm.nih.gov/Blast.cgi) | 1 |
| *........... Staphylinidae sp.* BMNH 1274658 | [1](https://blast.ncbi.nlm.nih.gov/Blast.cgi) | 1 |
| *..........* [*Elateriformia*](https://www.ncbi.nlm.nih.gov/Taxonomy/Browser/wwwtax.cgi?id=41087) | [3](https://blast.ncbi.nlm.nih.gov/Blast.cgi) | 2 |
| *...........* [*Endelus continentalis*](https://www.ncbi.nlm.nih.gov/Taxonomy/Browser/wwwtax.cgi?id=2984121) | [2](https://blast.ncbi.nlm.nih.gov/Blast.cgi) | 1 |
| *........... Chelonarium sp.* BMNH 840450 | [1](https://blast.ncbi.nlm.nih.gov/Blast.cgi) | 1 |
| *.........* [*Cybister brevis*](https://www.ncbi.nlm.nih.gov/Taxonomy/Browser/wwwtax.cgi?id=398584) | [2](https://blast.ncbi.nlm.nih.gov/Blast.cgi) | 1 |
| *........* [*Diptera*](https://www.ncbi.nlm.nih.gov/Taxonomy/Browser/wwwtax.cgi?id=7147) | [2](https://blast.ncbi.nlm.nih.gov/Blast.cgi) | 2 |
| *.........* [*Merzomyia westermanni*](https://www.ncbi.nlm.nih.gov/Taxonomy/Browser/wwwtax.cgi?id=2795681) | [1](https://blast.ncbi.nlm.nih.gov/Blast.cgi) | 1 |
| *.........* [*Coelophthinia thoracica*](https://www.ncbi.nlm.nih.gov/Taxonomy/Browser/wwwtax.cgi?id=2339105) | [1](https://blast.ncbi.nlm.nih.gov/Blast.cgi) | 1 |
| *........* [*Amphiesmenoptera*](https://www.ncbi.nlm.nih.gov/Taxonomy/Browser/wwwtax.cgi?id=85604) | [7](https://blast.ncbi.nlm.nih.gov/Blast.cgi) | 5 |
| *.........* [*Obtectomera*](https://www.ncbi.nlm.nih.gov/Taxonomy/Browser/wwwtax.cgi?id=104431) | [6](https://blast.ncbi.nlm.nih.gov/Blast.cgi) | 4 |
| *..........* [*Clanis*](https://www.ncbi.nlm.nih.gov/Taxonomy/Browser/wwwtax.cgi?id=215160) | [3](https://blast.ncbi.nlm.nih.gov/Blast.cgi) | 2 |
| *...........* [*Clanis deucalion*](https://www.ncbi.nlm.nih.gov/Taxonomy/Browser/wwwtax.cgi?id=1086238) | [1](https://blast.ncbi.nlm.nih.gov/Blast.cgi) | 1 |
| *...........* [*Clanis undulosa*](https://www.ncbi.nlm.nih.gov/Taxonomy/Browser/wwwtax.cgi?id=1086247) | [2](https://blast.ncbi.nlm.nih.gov/Blast.cgi) | 1 |
| *..........* [*Orthaga euadrusalis*](https://www.ncbi.nlm.nih.gov/Taxonomy/Browser/wwwtax.cgi?id=1418720) | [2](https://blast.ncbi.nlm.nih.gov/Blast.cgi) | 1 |
| *..........* [*Nymphalis ladakensis*](https://www.ncbi.nlm.nih.gov/Taxonomy/Browser/wwwtax.cgi?id=1916413) | [1](https://blast.ncbi.nlm.nih.gov/Blast.cgi) | 1 |
| *.........* [*Trichostegia minor*](https://www.ncbi.nlm.nih.gov/Taxonomy/Browser/wwwtax.cgi?id=1271749) | [1](https://blast.ncbi.nlm.nih.gov/Blast.cgi) | 1 |
| *........* [*Apocrita*](https://www.ncbi.nlm.nih.gov/Taxonomy/Browser/wwwtax.cgi?id=7400) | [71](https://blast.ncbi.nlm.nih.gov/Blast.cgi) | 14 |
| *.........* [*Aculeata*](https://www.ncbi.nlm.nih.gov/Taxonomy/Browser/wwwtax.cgi?id=7434) | [60](https://blast.ncbi.nlm.nih.gov/Blast.cgi) | 8 |
| *..........* [*Anthophila*](https://www.ncbi.nlm.nih.gov/Taxonomy/Browser/wwwtax.cgi?id=3042114) | [58](https://blast.ncbi.nlm.nih.gov/Blast.cgi) | 7 |
| *...........* [*Apis*](https://www.ncbi.nlm.nih.gov/Taxonomy/Browser/wwwtax.cgi?id=7459) | [55](https://blast.ncbi.nlm.nih.gov/Blast.cgi) | 4 |
| *............* [*Apis cerana*](https://www.ncbi.nlm.nih.gov/Taxonomy/Browser/wwwtax.cgi?id=7461) | [50](https://blast.ncbi.nlm.nih.gov/Blast.cgi) | 3 |
| *.............* [*Apis cerana cerana*](https://www.ncbi.nlm.nih.gov/Taxonomy/Browser/wwwtax.cgi?id=94128) | [1](https://blast.ncbi.nlm.nih.gov/Blast.cgi) | 1 |
| *.............* [*Apis cerana japonica*](https://www.ncbi.nlm.nih.gov/Taxonomy/Browser/wwwtax.cgi?id=292787) | [3](https://blast.ncbi.nlm.nih.gov/Blast.cgi) | 1 |
| *............* [*Apis nigrocincta*](https://www.ncbi.nlm.nih.gov/Taxonomy/Browser/wwwtax.cgi?id=83312) | [1](https://blast.ncbi.nlm.nih.gov/Blast.cgi) | 1 |
| *...........* [*Andrena incertae sedis*](https://www.ncbi.nlm.nih.gov/Taxonomy/Browser/wwwtax.cgi?id=1126400) | [2](https://blast.ncbi.nlm.nih.gov/Blast.cgi) | 2 |
| *............* [*Andrena camellia*](https://www.ncbi.nlm.nih.gov/Taxonomy/Browser/wwwtax.cgi?id=1862692) | [1](https://blast.ncbi.nlm.nih.gov/Blast.cgi) | 1 |
| *............* [*Andrena minutula*](https://www.ncbi.nlm.nih.gov/Taxonomy/Browser/wwwtax.cgi?id=1190802) | [1](https://blast.ncbi.nlm.nih.gov/Blast.cgi) | 1 |
| *...........* [*Lasioglossum pauxillum*](https://www.ncbi.nlm.nih.gov/Taxonomy/Browser/wwwtax.cgi?id=88516) | [1](https://blast.ncbi.nlm.nih.gov/Blast.cgi) | 1 |
| *..........* [*Nylanderia flavipes*](https://www.ncbi.nlm.nih.gov/Taxonomy/Browser/wwwtax.cgi?id=67766) | [2](https://blast.ncbi.nlm.nih.gov/Blast.cgi) | 1 |
| *.........* [*Proctotrupomorpha*](https://www.ncbi.nlm.nih.gov/Taxonomy/Browser/wwwtax.cgi?id=1955251) | [5](https://blast.ncbi.nlm.nih.gov/Blast.cgi) | 4 |
| *..........* [*Megastigmus duclouxiana*](https://www.ncbi.nlm.nih.gov/Taxonomy/Browser/wwwtax.cgi?id=2941156) | [1](https://blast.ncbi.nlm.nih.gov/Blast.cgi) | 1 |
| *..........* [*Trichopria drosophilae*](https://www.ncbi.nlm.nih.gov/Taxonomy/Browser/wwwtax.cgi?id=1507179) | [2](https://blast.ncbi.nlm.nih.gov/Blast.cgi) | 1 |
| *..........* [*Figitidae*](https://www.ncbi.nlm.nih.gov/Taxonomy/Browser/wwwtax.cgi?id=44353) | [2](https://blast.ncbi.nlm.nih.gov/Blast.cgi) | 2 |
| *........... Figites sp.* ZJUH 20220009 | [1](https://blast.ncbi.nlm.nih.gov/Blast.cgi) | 1 |
| *........... Endecameris sp.* ZJUH 20220006 | [1](https://blast.ncbi.nlm.nih.gov/Blast.cgi) | 1 |
| *.........* [*Braconidae*](https://www.ncbi.nlm.nih.gov/Taxonomy/Browser/wwwtax.cgi?id=7402) | [6](https://blast.ncbi.nlm.nih.gov/Blast.cgi) | 2 |
| *.......... Homolobus sp.* QL-2013 | [1](https://blast.ncbi.nlm.nih.gov/Blast.cgi) | 1 |
| *..........* [*Chelonus formosanus*](https://www.ncbi.nlm.nih.gov/Taxonomy/Browser/wwwtax.cgi?id=2739011) | [5](https://blast.ncbi.nlm.nih.gov/Blast.cgi) | 1 |
| *........ Coniopteryx sp.* YW-2016 | [1](https://blast.ncbi.nlm.nih.gov/Blast.cgi) | 1 |
| *.......* [*Hemiptera*](https://www.ncbi.nlm.nih.gov/Taxonomy/Browser/wwwtax.cgi?id=7524) | [22](https://blast.ncbi.nlm.nih.gov/Blast.cgi) | 15 |
| *........* [*Cicadomorpha*](https://www.ncbi.nlm.nih.gov/Taxonomy/Browser/wwwtax.cgi?id=33365) | [11](https://blast.ncbi.nlm.nih.gov/Blast.cgi) | 7 |
| *.........* [*Cicadellidae*](https://www.ncbi.nlm.nih.gov/Taxonomy/Browser/wwwtax.cgi?id=30102) | [10](https://blast.ncbi.nlm.nih.gov/Blast.cgi) | 6 |
| *..........* [*Cicadellini*](https://www.ncbi.nlm.nih.gov/Taxonomy/Browser/wwwtax.cgi?id=565680) | [7](https://blast.ncbi.nlm.nih.gov/Blast.cgi) | 4 |
| *...........* [*Atkinsoniella*](https://www.ncbi.nlm.nih.gov/Taxonomy/Browser/wwwtax.cgi?id=565723) | [3](https://blast.ncbi.nlm.nih.gov/Blast.cgi) | 2 |
| *............* [*Atkinsoniella zizhongi*](https://www.ncbi.nlm.nih.gov/Taxonomy/Browser/wwwtax.cgi?id=2971512) | [2](https://blast.ncbi.nlm.nih.gov/Blast.cgi) | 1 |
| *............* [*Atkinsoniella trimaculata*](https://www.ncbi.nlm.nih.gov/Taxonomy/Browser/wwwtax.cgi?id=700742) | [1](https://blast.ncbi.nlm.nih.gov/Blast.cgi) | 1 |
| *...........* [*Stenatkina angustata*](https://www.ncbi.nlm.nih.gov/Taxonomy/Browser/wwwtax.cgi?id=700816) | [2](https://blast.ncbi.nlm.nih.gov/Blast.cgi) | 1 |
| *...........* [*Paratkina nigrifasciana*](https://www.ncbi.nlm.nih.gov/Taxonomy/Browser/wwwtax.cgi?id=3004265) | [2](https://blast.ncbi.nlm.nih.gov/Blast.cgi) | 1 |
| *..........* [*Evacanthus danmainus*](https://www.ncbi.nlm.nih.gov/Taxonomy/Browser/wwwtax.cgi?id=2840405) | [1](https://blast.ncbi.nlm.nih.gov/Blast.cgi) | 1 |
| *..........* [*Krisna nigromarginata*](https://www.ncbi.nlm.nih.gov/Taxonomy/Browser/wwwtax.cgi?id=1962557) | [2](https://blast.ncbi.nlm.nih.gov/Blast.cgi) | 1 |
| *.........* [*Cosmoscarta mandarina*](https://www.ncbi.nlm.nih.gov/Taxonomy/Browser/wwwtax.cgi?id=797794) | [1](https://blast.ncbi.nlm.nih.gov/Blast.cgi) | 1 |
| *........* [*Aphidomorpha*](https://www.ncbi.nlm.nih.gov/Taxonomy/Browser/wwwtax.cgi?id=33380) | [9](https://blast.ncbi.nlm.nih.gov/Blast.cgi) | 7 |
| *.........* [*Aphididae*](https://www.ncbi.nlm.nih.gov/Taxonomy/Browser/wwwtax.cgi?id=27482) | [8](https://blast.ncbi.nlm.nih.gov/Blast.cgi) | 6 |
| *..........* [*Anomalosiphum*](https://www.ncbi.nlm.nih.gov/Taxonomy/Browser/wwwtax.cgi?id=1173403) | [3](https://blast.ncbi.nlm.nih.gov/Blast.cgi) | 2 |
| *...........* [*Anomalosiphum tiomanensis*](https://www.ncbi.nlm.nih.gov/Taxonomy/Browser/wwwtax.cgi?id=1173405) | [1](https://blast.ncbi.nlm.nih.gov/Blast.cgi) | 1 |
| *...........* [*Anomalosiphum takahashii*](https://www.ncbi.nlm.nih.gov/Taxonomy/Browser/wwwtax.cgi?id=1173404) | [2](https://blast.ncbi.nlm.nih.gov/Blast.cgi) | 1 |
| *..........* [*Aphidini*](https://www.ncbi.nlm.nih.gov/Taxonomy/Browser/wwwtax.cgi?id=33387) | [2](https://blast.ncbi.nlm.nih.gov/Blast.cgi) | 2 |
| *...........* [*Schizaphis scirpi*](https://www.ncbi.nlm.nih.gov/Taxonomy/Browser/wwwtax.cgi?id=511024) | [1](https://blast.ncbi.nlm.nih.gov/Blast.cgi) | 1 |
| *...........* [*Rhopalosiphum cerasifoliae*](https://www.ncbi.nlm.nih.gov/Taxonomy/Browser/wwwtax.cgi?id=168653) | [1](https://blast.ncbi.nlm.nih.gov/Blast.cgi) | 1 |
| *..........* [*Cinara tujafilina*](https://www.ncbi.nlm.nih.gov/Taxonomy/Browser/wwwtax.cgi?id=198323) | [1](https://blast.ncbi.nlm.nih.gov/Blast.cgi) | 1 |
| *.......... Allaphis sp.* 1 YL-2021a | [2](https://blast.ncbi.nlm.nih.gov/Blast.cgi) | 1 |
| *.........* [*Adelges japonicus*](https://www.ncbi.nlm.nih.gov/Taxonomy/Browser/wwwtax.cgi?id=133064) | [1](https://blast.ncbi.nlm.nih.gov/Blast.cgi) | 1 |
| *........* [*Rhagovelia reitteri*](https://www.ncbi.nlm.nih.gov/Taxonomy/Browser/wwwtax.cgi?id=2581066) | [2](https://blast.ncbi.nlm.nih.gov/Blast.cgi) | 1 |
| *......* [*Epiophlebia superstes*](https://www.ncbi.nlm.nih.gov/Taxonomy/Browser/wwwtax.cgi?id=126247) | [2](https://blast.ncbi.nlm.nih.gov/Blast.cgi) | 1 |
| *.....* [*Multicrustacea*](https://www.ncbi.nlm.nih.gov/Taxonomy/Browser/wwwtax.cgi?id=2172821) | [8](https://blast.ncbi.nlm.nih.gov/Blast.cgi) | 3 |
| *......* [*Pleocyemata*](https://www.ncbi.nlm.nih.gov/Taxonomy/Browser/wwwtax.cgi?id=6692) | [4](https://blast.ncbi.nlm.nih.gov/Blast.cgi) | 2 |
| *....... Chaceon sp.* BZ-2016 | [1](https://blast.ncbi.nlm.nih.gov/Blast.cgi) | 1 |
| *.......* [*Cambarus robustus*](https://www.ncbi.nlm.nih.gov/Taxonomy/Browser/wwwtax.cgi?id=1240928) | [3](https://blast.ncbi.nlm.nih.gov/Blast.cgi) | 1 |
| *......* [*Zausodes septimus*](https://www.ncbi.nlm.nih.gov/Taxonomy/Browser/wwwtax.cgi?id=673207) | [4](https://blast.ncbi.nlm.nih.gov/Blast.cgi) | 1 |
| *....* [*Ixodidae*](https://www.ncbi.nlm.nih.gov/Taxonomy/Browser/wwwtax.cgi?id=6939) | [5](https://blast.ncbi.nlm.nih.gov/Blast.cgi) | 4 |
| *.....* [*Ixodes*](https://www.ncbi.nlm.nih.gov/Taxonomy/Browser/wwwtax.cgi?id=6944) | [3](https://blast.ncbi.nlm.nih.gov/Blast.cgi) | 2 |
| *......* [*Ixodes loricatus*](https://www.ncbi.nlm.nih.gov/Taxonomy/Browser/wwwtax.cgi?id=59649) | [1](https://blast.ncbi.nlm.nih.gov/Blast.cgi) | 1 |
| *......* [*Ixodes woyliei*](https://www.ncbi.nlm.nih.gov/Taxonomy/Browser/wwwtax.cgi?id=1933095) | [2](https://blast.ncbi.nlm.nih.gov/Blast.cgi) | 1 |
| *.....* [*Dermacentor (Indocentor) sp.*](https://www.ncbi.nlm.nih.gov/Taxonomy/Browser/wwwtax.cgi?id=2927972) | [1](https://blast.ncbi.nlm.nih.gov/Blast.cgi) | 1 |
| *.....* [*Hyalomma anatolicum*](https://www.ncbi.nlm.nih.gov/Taxonomy/Browser/wwwtax.cgi?id=176092) | [1](https://blast.ncbi.nlm.nih.gov/Blast.cgi) | 1 |
| *...* [*Lophotrochozoa*](https://www.ncbi.nlm.nih.gov/Taxonomy/Browser/wwwtax.cgi?id=1206795) | [8](https://blast.ncbi.nlm.nih.gov/Blast.cgi) | 4 |
| *....* [*Caenogastropoda*](https://www.ncbi.nlm.nih.gov/Taxonomy/Browser/wwwtax.cgi?id=69555) | [4](https://blast.ncbi.nlm.nih.gov/Blast.cgi) | 2 |
| *.....* [*Euspira gilva*](https://www.ncbi.nlm.nih.gov/Taxonomy/Browser/wwwtax.cgi?id=2713330) | [3](https://blast.ncbi.nlm.nih.gov/Blast.cgi) | 1 |
| *..... Splendrillia sp.* 2 MNHN IM 2013-9750 | [1](https://blast.ncbi.nlm.nih.gov/Blast.cgi) | 1 |
| *....* [*Annelida*](https://www.ncbi.nlm.nih.gov/Taxonomy/Browser/wwwtax.cgi?id=6340) | [4](https://blast.ncbi.nlm.nih.gov/Blast.cgi) | 2 |
| *.....* [*Theromyzon tessulatum*](https://www.ncbi.nlm.nih.gov/Taxonomy/Browser/wwwtax.cgi?id=13286) | [3](https://blast.ncbi.nlm.nih.gov/Blast.cgi) | 1 |
| *..... Pisione sp.* YZ-2018 | [1](https://blast.ncbi.nlm.nih.gov/Blast.cgi) | 1 |
| *.* [*Helminthocladia australis*](https://www.ncbi.nlm.nih.gov/Taxonomy/Browser/wwwtax.cgi?id=260093) | [1](https://blast.ncbi.nlm.nih.gov/Blast.cgi) | 1 |
| CYB used Target sequence (137 bp): ACATGAAATATCGGCGTCCTGTTATTATTTGCCGTTATAGCCACCGCATTCATAGGGTACGTCTTACCATGGGGTCAAATATCCTTTTGAGGTGCAACAGTAATTACAAATTTACTGTCAGCCATCCCTTACATCGG (Source: Ripa et al in prep) | | | |

| Table S1 b: In silico analysis for *N. a. anomalus.*  *Number Hits: Sequences producing significant alignment* (unlimited coverage) | | | | |
| --- | --- | --- | --- | --- |
| **Organism** | **Blast Name** | **Score** | **Number Hits** | **Percent identity** |
| [*Gnathostomata*](https://www.ncbi.nlm.nih.gov/Taxonomy/Browser/wwwtax.cgi?id=7776) | [vertebrates](https://www.ncbi.nlm.nih.gov/Taxonomy/Browser/wwwtax.cgi?id=7742) |  | [3359](https://blast.ncbi.nlm.nih.gov/Blast.cgi) |  |
| *.*[*Euteleostomi*](https://www.ncbi.nlm.nih.gov/Taxonomy/Browser/wwwtax.cgi?id=117571) | [vertebrates](https://www.ncbi.nlm.nih.gov/Taxonomy/Browser/wwwtax.cgi?id=7742) |  | [3305](https://blast.ncbi.nlm.nih.gov/Blast.cgi) |  |
| *..*[*Amniota*](https://www.ncbi.nlm.nih.gov/Taxonomy/Browser/wwwtax.cgi?id=32524) | [vertebrates](https://www.ncbi.nlm.nih.gov/Taxonomy/Browser/wwwtax.cgi?id=7742) |  | [2932](https://blast.ncbi.nlm.nih.gov/Blast.cgi) |  |
| *...*[*Theria*](https://www.ncbi.nlm.nih.gov/Taxonomy/Browser/wwwtax.cgi?id=32525) | [mammals](https://www.ncbi.nlm.nih.gov/Taxonomy/Browser/wwwtax.cgi?id=40674) |  | [2360](https://blast.ncbi.nlm.nih.gov/Blast.cgi) |  |
| *....*[*Eutheria*](https://www.ncbi.nlm.nih.gov/Taxonomy/Browser/wwwtax.cgi?id=9347) | [placentals](https://www.ncbi.nlm.nih.gov/Taxonomy/Browser/wwwtax.cgi?id=9347) |  | [2333](https://blast.ncbi.nlm.nih.gov/Blast.cgi) |  |
| *.....*[*Boreoeutheria*](https://www.ncbi.nlm.nih.gov/Taxonomy/Browser/wwwtax.cgi?id=1437010) | [placentals](https://www.ncbi.nlm.nih.gov/Taxonomy/Browser/wwwtax.cgi?id=9347) |  | [2323](https://blast.ncbi.nlm.nih.gov/Blast.cgi) |  |
| *......*[*Laurasiatheria*](https://www.ncbi.nlm.nih.gov/Taxonomy/Browser/wwwtax.cgi?id=314145) | [placentals](https://www.ncbi.nlm.nih.gov/Taxonomy/Browser/wwwtax.cgi?id=9347) |  | [1693](https://blast.ncbi.nlm.nih.gov/Blast.cgi) |  |
| *.......*[*Eulipotyphla*](https://www.ncbi.nlm.nih.gov/Taxonomy/Browser/wwwtax.cgi?id=9362) | [insectivores](https://www.ncbi.nlm.nih.gov/Taxonomy/Browser/wwwtax.cgi?id=9362) |  | [635](https://blast.ncbi.nlm.nih.gov/Blast.cgi) |  |
| *........*[*Soricidae*](https://www.ncbi.nlm.nih.gov/Taxonomy/Browser/wwwtax.cgi?id=9376) | [insectivores](https://www.ncbi.nlm.nih.gov/Taxonomy/Browser/wwwtax.cgi?id=9362) |  | [632](https://blast.ncbi.nlm.nih.gov/Blast.cgi) |  |
| *.........*[*Soricinae*](https://www.ncbi.nlm.nih.gov/Taxonomy/Browser/wwwtax.cgi?id=183663) | [insectivores](https://www.ncbi.nlm.nih.gov/Taxonomy/Browser/wwwtax.cgi?id=9362) |  | [548](https://blast.ncbi.nlm.nih.gov/Blast.cgi) |  |
| *..........*[*Neomys*](https://www.ncbi.nlm.nih.gov/Taxonomy/Browser/wwwtax.cgi?id=52813) | [insectivores](https://www.ncbi.nlm.nih.gov/Taxonomy/Browser/wwwtax.cgi?id=9362) |  | [166](https://blast.ncbi.nlm.nih.gov/Blast.cgi) |  |
| *...........*[*Neomys anomalus*](https://www.ncbi.nlm.nih.gov/Taxonomy/Browser/wwwtax.cgi?id=52814) | [insectivores](https://www.ncbi.nlm.nih.gov/Taxonomy/Browser/wwwtax.cgi?id=9362) | 189 | [151](https://blast.ncbi.nlm.nih.gov/Blast.cgi) | 100 to 95.10 |
| *...........*[*Neomys anomalus anomalus*](https://www.ncbi.nlm.nih.gov/Taxonomy/Browser/wwwtax.cgi?id=1521023) | [insectivores](https://www.ncbi.nlm.nih.gov/Taxonomy/Browser/wwwtax.cgi?id=9362) | 189 | [8](https://blast.ncbi.nlm.nih.gov/Blast.cgi) |
| *...........*[*Neomys anomalus milleri*](https://www.ncbi.nlm.nih.gov/Taxonomy/Browser/wwwtax.cgi?id=1521024) | [insectivores](https://www.ncbi.nlm.nih.gov/Taxonomy/Browser/wwwtax.cgi?id=9362) | 172 | [6](https://blast.ncbi.nlm.nih.gov/Blast.cgi) |
| *...........*[*Neomys teres*](https://www.ncbi.nlm.nih.gov/Taxonomy/Browser/wwwtax.cgi?id=103776) | [insectivores](https://www.ncbi.nlm.nih.gov/Taxonomy/Browser/wwwtax.cgi?id=9362) | 150 | [1](https://blast.ncbi.nlm.nih.gov/Blast.cgi) | < 93.14 |
| *..........*[*Blarinella wardi*](https://www.ncbi.nlm.nih.gov/Taxonomy/Browser/wwwtax.cgi?id=1159883) | [insectivores](https://www.ncbi.nlm.nih.gov/Taxonomy/Browser/wwwtax.cgi?id=9362) | 134 | [22](https://blast.ncbi.nlm.nih.gov/Blast.cgi) |
| *..........*[*Sorex bedfordiae*](https://www.ncbi.nlm.nih.gov/Taxonomy/Browser/wwwtax.cgi?id=862701) | [insectivores](https://www.ncbi.nlm.nih.gov/Taxonomy/Browser/wwwtax.cgi?id=9362) | 128 | [35](https://blast.ncbi.nlm.nih.gov/Blast.cgi) |
| *..........*[*Sorex cylindricauda*](https://www.ncbi.nlm.nih.gov/Taxonomy/Browser/wwwtax.cgi?id=268767) | [insectivores](https://www.ncbi.nlm.nih.gov/Taxonomy/Browser/wwwtax.cgi?id=9362) | 122 | [1](https://blast.ncbi.nlm.nih.gov/Blast.cgi) |
| *..........*[*Chimarrogale varennei*](https://www.ncbi.nlm.nih.gov/Taxonomy/Browser/wwwtax.cgi?id=1968511) | [insectivores](https://www.ncbi.nlm.nih.gov/Taxonomy/Browser/wwwtax.cgi?id=9362) | 121 | [4](https://blast.ncbi.nlm.nih.gov/Blast.cgi) |
| *..........*[*Anourosorex squamipes*](https://www.ncbi.nlm.nih.gov/Taxonomy/Browser/wwwtax.cgi?id=127560) | [insectivores](https://www.ncbi.nlm.nih.gov/Taxonomy/Browser/wwwtax.cgi?id=9362) | 117 | [3](https://blast.ncbi.nlm.nih.gov/Blast.cgi) |
| *..........*[*Chimarrogale styani*](https://www.ncbi.nlm.nih.gov/Taxonomy/Browser/wwwtax.cgi?id=1365920) | [insectivores](https://www.ncbi.nlm.nih.gov/Taxonomy/Browser/wwwtax.cgi?id=9362) | 111 | [4](https://blast.ncbi.nlm.nih.gov/Blast.cgi) |
| *..........*[*Episoriculus soluensis*](https://www.ncbi.nlm.nih.gov/Taxonomy/Browser/wwwtax.cgi?id=2715698) | [insectivores](https://www.ncbi.nlm.nih.gov/Taxonomy/Browser/wwwtax.cgi?id=9362) | 111 | [1](https://blast.ncbi.nlm.nih.gov/Blast.cgi) |
| *..........*[*Sorex yukonicus*](https://www.ncbi.nlm.nih.gov/Taxonomy/Browser/wwwtax.cgi?id=910940) | [insectivores](https://www.ncbi.nlm.nih.gov/Taxonomy/Browser/wwwtax.cgi?id=9362) | 111 | [28](https://blast.ncbi.nlm.nih.gov/Blast.cgi) |
| *..........*[*Episoriculus caudatus soluensis*](https://www.ncbi.nlm.nih.gov/Taxonomy/Browser/wwwtax.cgi?id=268765) | [insectivores](https://www.ncbi.nlm.nih.gov/Taxonomy/Browser/wwwtax.cgi?id=9362) | 111 | [2](https://blast.ncbi.nlm.nih.gov/Blast.cgi) |
| *..........*[*Sorex buchariensis*](https://www.ncbi.nlm.nih.gov/Taxonomy/Browser/wwwtax.cgi?id=2491181) | [insectivores](https://www.ncbi.nlm.nih.gov/Taxonomy/Browser/wwwtax.cgi?id=9362) | 108 | [3](https://blast.ncbi.nlm.nih.gov/Blast.cgi) |
| *..........*[*Sorex minutissimus*](https://www.ncbi.nlm.nih.gov/Taxonomy/Browser/wwwtax.cgi?id=62274) | [insectivores](https://www.ncbi.nlm.nih.gov/Taxonomy/Browser/wwwtax.cgi?id=9362) | 106 | [111](https://blast.ncbi.nlm.nih.gov/Blast.cgi) |
| *..........*[*Sorex coronatus*](https://www.ncbi.nlm.nih.gov/Taxonomy/Browser/wwwtax.cgi?id=62895) | [insectivores](https://www.ncbi.nlm.nih.gov/Taxonomy/Browser/wwwtax.cgi?id=9362) | 106 | [25](https://blast.ncbi.nlm.nih.gov/Blast.cgi) |
| *..........*[*Chodsigoa dabieshanensis*](https://www.ncbi.nlm.nih.gov/Taxonomy/Browser/wwwtax.cgi?id=2928188) | [insectivores](https://www.ncbi.nlm.nih.gov/Taxonomy/Browser/wwwtax.cgi?id=9362) | 106 | [7](https://blast.ncbi.nlm.nih.gov/Blast.cgi) |
| *..........*[*Episoriculus fumidus*](https://www.ncbi.nlm.nih.gov/Taxonomy/Browser/wwwtax.cgi?id=150090) | [insectivores](https://www.ncbi.nlm.nih.gov/Taxonomy/Browser/wwwtax.cgi?id=9362) | 106 | [7](https://blast.ncbi.nlm.nih.gov/Blast.cgi) |
| *..........*[*Nectogale elegans*](https://www.ncbi.nlm.nih.gov/Taxonomy/Browser/wwwtax.cgi?id=268758) | [insectivores](https://www.ncbi.nlm.nih.gov/Taxonomy/Browser/wwwtax.cgi?id=9362) | 104 | [2](https://blast.ncbi.nlm.nih.gov/Blast.cgi) |
| *..........*[*Sorex granarius*](https://www.ncbi.nlm.nih.gov/Taxonomy/Browser/wwwtax.cgi?id=62894) | [insectivores](https://www.ncbi.nlm.nih.gov/Taxonomy/Browser/wwwtax.cgi?id=9362) | 100 | [5](https://blast.ncbi.nlm.nih.gov/Blast.cgi) |
| *..........*[*Sorex sinalis*](https://www.ncbi.nlm.nih.gov/Taxonomy/Browser/wwwtax.cgi?id=2083004) | [insectivores](https://www.ncbi.nlm.nih.gov/Taxonomy/Browser/wwwtax.cgi?id=9362) | 100 | [4](https://blast.ncbi.nlm.nih.gov/Blast.cgi) |
| *..........*[*Chodsigoa hypsibia*](https://www.ncbi.nlm.nih.gov/Taxonomy/Browser/wwwtax.cgi?id=862699) | [insectivores](https://www.ncbi.nlm.nih.gov/Taxonomy/Browser/wwwtax.cgi?id=9362) | 100 | [4](https://blast.ncbi.nlm.nih.gov/Blast.cgi) |
| *..........*[*Sorex fumeus*](https://www.ncbi.nlm.nih.gov/Taxonomy/Browser/wwwtax.cgi?id=62283) | [insectivores](https://www.ncbi.nlm.nih.gov/Taxonomy/Browser/wwwtax.cgi?id=9362) | 100 | [11](https://blast.ncbi.nlm.nih.gov/Blast.cgi) |
| *..........*[*Sorex excelsus*](https://www.ncbi.nlm.nih.gov/Taxonomy/Browser/wwwtax.cgi?id=62901) | [insectivores](https://www.ncbi.nlm.nih.gov/Taxonomy/Browser/wwwtax.cgi?id=9362) | 100 | [1](https://blast.ncbi.nlm.nih.gov/Blast.cgi) |
| *..........*[*Sorex araneus*](https://www.ncbi.nlm.nih.gov/Taxonomy/Browser/wwwtax.cgi?id=42254) | [insectivores](https://www.ncbi.nlm.nih.gov/Taxonomy/Browser/wwwtax.cgi?id=9362) | 100 | [10](https://blast.ncbi.nlm.nih.gov/Blast.cgi) |
| *..........*[*Sorex cinereus*](https://www.ncbi.nlm.nih.gov/Taxonomy/Browser/wwwtax.cgi?id=36803) | [insectivores](https://www.ncbi.nlm.nih.gov/Taxonomy/Browser/wwwtax.cgi?id=9362) | 100 | [1](https://blast.ncbi.nlm.nih.gov/Blast.cgi) |
| *..........*[*Sorex dispar dispar*](https://www.ncbi.nlm.nih.gov/Taxonomy/Browser/wwwtax.cgi?id=395346) | [insectivores](https://www.ncbi.nlm.nih.gov/Taxonomy/Browser/wwwtax.cgi?id=9362) | 100 | [1](https://blast.ncbi.nlm.nih.gov/Blast.cgi) |
| *..........*[*Chodsigoa parva*](https://www.ncbi.nlm.nih.gov/Taxonomy/Browser/wwwtax.cgi?id=2023227) | [insectivores](https://www.ncbi.nlm.nih.gov/Taxonomy/Browser/wwwtax.cgi?id=9362) | 99.0 | [9](https://blast.ncbi.nlm.nih.gov/Blast.cgi) |
| *..........*[*Sorex caecutiens*](https://www.ncbi.nlm.nih.gov/Taxonomy/Browser/wwwtax.cgi?id=62276) | [insectivores](https://www.ncbi.nlm.nih.gov/Taxonomy/Browser/wwwtax.cgi?id=9362) | 99.0 | [1](https://blast.ncbi.nlm.nih.gov/Blast.cgi) |
| *..........*[*Sorex shinto*](https://www.ncbi.nlm.nih.gov/Taxonomy/Browser/wwwtax.cgi?id=62286) | [insectivores](https://www.ncbi.nlm.nih.gov/Taxonomy/Browser/wwwtax.cgi?id=9362) | 97.1 | [6](https://blast.ncbi.nlm.nih.gov/Blast.cgi) |
| *..........*[*Sorex cansulus*](https://www.ncbi.nlm.nih.gov/Taxonomy/Browser/wwwtax.cgi?id=2491182) | [insectivores](https://www.ncbi.nlm.nih.gov/Taxonomy/Browser/wwwtax.cgi?id=9362) | 95.3 | [1](https://blast.ncbi.nlm.nih.gov/Blast.cgi) |
| *..........*[*Sorex antinorii*](https://www.ncbi.nlm.nih.gov/Taxonomy/Browser/wwwtax.cgi?id=268768) | [insectivores](https://www.ncbi.nlm.nih.gov/Taxonomy/Browser/wwwtax.cgi?id=9362) | 95.3 | [3](https://blast.ncbi.nlm.nih.gov/Blast.cgi) |
| *..........*[*Sorex asper*](https://www.ncbi.nlm.nih.gov/Taxonomy/Browser/wwwtax.cgi?id=62897) | [insectivores](https://www.ncbi.nlm.nih.gov/Taxonomy/Browser/wwwtax.cgi?id=9362) | 95.3 | [20](https://blast.ncbi.nlm.nih.gov/Blast.cgi) |
| *..........*[*Sorex unguiculatus*](https://www.ncbi.nlm.nih.gov/Taxonomy/Browser/wwwtax.cgi?id=62275) | [insectivores](https://www.ncbi.nlm.nih.gov/Taxonomy/Browser/wwwtax.cgi?id=9362) | 95.3 | [34](https://blast.ncbi.nlm.nih.gov/Blast.cgi) |
| *..........*[*Sorex isodon*](https://www.ncbi.nlm.nih.gov/Taxonomy/Browser/wwwtax.cgi?id=62281) | [insectivores](https://www.ncbi.nlm.nih.gov/Taxonomy/Browser/wwwtax.cgi?id=9362) | 84.2 | [16](https://blast.ncbi.nlm.nih.gov/Blast.cgi) |
| *.........*[*Crocidura montis*](https://www.ncbi.nlm.nih.gov/Taxonomy/Browser/wwwtax.cgi?id=516697) | [insectivores](https://www.ncbi.nlm.nih.gov/Taxonomy/Browser/wwwtax.cgi?id=9362) | 97.1 | [4](https://blast.ncbi.nlm.nih.gov/Blast.cgi) |
| *.........*[*Crocidura ichnusae*](https://www.ncbi.nlm.nih.gov/Taxonomy/Browser/wwwtax.cgi?id=413580) | [insectivores](https://www.ncbi.nlm.nih.gov/Taxonomy/Browser/wwwtax.cgi?id=9362) | 97.1 | [22](https://blast.ncbi.nlm.nih.gov/Blast.cgi) |
| *.........*[*Crocidura latona*](https://www.ncbi.nlm.nih.gov/Taxonomy/Browser/wwwtax.cgi?id=516696) | [insectivores](https://www.ncbi.nlm.nih.gov/Taxonomy/Browser/wwwtax.cgi?id=9362) | 91.6 | [2](https://blast.ncbi.nlm.nih.gov/Blast.cgi) |
| *.........*[*Crocidura sp. indet. Clade107*](https://www.ncbi.nlm.nih.gov/Taxonomy/Browser/wwwtax.cgi?id=2941041) | [insectivores](https://www.ncbi.nlm.nih.gov/Taxonomy/Browser/wwwtax.cgi?id=9362) | 91.6 | [1](https://blast.ncbi.nlm.nih.gov/Blast.cgi) |
| *.........*[*Crocidura obscurior*](https://www.ncbi.nlm.nih.gov/Taxonomy/Browser/wwwtax.cgi?id=148977) | [insectivores](https://www.ncbi.nlm.nih.gov/Taxonomy/Browser/wwwtax.cgi?id=9362) | 89.8 | [10](https://blast.ncbi.nlm.nih.gov/Blast.cgi) |
| *.........*[*Crocidura bottegi*](https://www.ncbi.nlm.nih.gov/Taxonomy/Browser/wwwtax.cgi?id=214465) | [insectivores](https://www.ncbi.nlm.nih.gov/Taxonomy/Browser/wwwtax.cgi?id=9362) | 89.8 | [1](https://blast.ncbi.nlm.nih.gov/Blast.cgi) |
| *.........*[*Crocidura orii*](https://www.ncbi.nlm.nih.gov/Taxonomy/Browser/wwwtax.cgi?id=268755) | [insectivores](https://www.ncbi.nlm.nih.gov/Taxonomy/Browser/wwwtax.cgi?id=9362) | 89.8 | [1](https://blast.ncbi.nlm.nih.gov/Blast.cgi) |
| *.........*[*Suncus remyi*](https://www.ncbi.nlm.nih.gov/Taxonomy/Browser/wwwtax.cgi?id=148983) | [insectivores](https://www.ncbi.nlm.nih.gov/Taxonomy/Browser/wwwtax.cgi?id=9362) | 87.9 | [1](https://blast.ncbi.nlm.nih.gov/Blast.cgi) |
| *.........*[*Crocidura littoralis*](https://www.ncbi.nlm.nih.gov/Taxonomy/Browser/wwwtax.cgi?id=214445) | [insectivores](https://www.ncbi.nlm.nih.gov/Taxonomy/Browser/wwwtax.cgi?id=9362) | 84.2 | [2](https://blast.ncbi.nlm.nih.gov/Blast.cgi) |
| *.........*[*Crocidura russula*](https://www.ncbi.nlm.nih.gov/Taxonomy/Browser/wwwtax.cgi?id=36802) | [insectivores](https://www.ncbi.nlm.nih.gov/Taxonomy/Browser/wwwtax.cgi?id=9362) | 82.4 | [1](https://blast.ncbi.nlm.nih.gov/Blast.cgi) |
| *.........*[*Crocidura foetida*](https://www.ncbi.nlm.nih.gov/Taxonomy/Browser/wwwtax.cgi?id=61089) | [insectivores](https://www.ncbi.nlm.nih.gov/Taxonomy/Browser/wwwtax.cgi?id=9362) | 78.7 | [5](https://blast.ncbi.nlm.nih.gov/Blast.cgi) |
| *.........*[*Crocidura miya*](https://www.ncbi.nlm.nih.gov/Taxonomy/Browser/wwwtax.cgi?id=481573) | [insectivores](https://www.ncbi.nlm.nih.gov/Taxonomy/Browser/wwwtax.cgi?id=9362) | 78.7 | [1](https://blast.ncbi.nlm.nih.gov/Blast.cgi) |
| *.........*[*Crocidura quasielongata*](https://www.ncbi.nlm.nih.gov/Taxonomy/Browser/wwwtax.cgi?id=2873341) | [insectivores](https://www.ncbi.nlm.nih.gov/Taxonomy/Browser/wwwtax.cgi?id=9362) | 75.0 | [6](https://blast.ncbi.nlm.nih.gov/Blast.cgi) |
| *.........*[*Crocidura rapax kurodai*](https://www.ncbi.nlm.nih.gov/Taxonomy/Browser/wwwtax.cgi?id=155306) | [insectivores](https://www.ncbi.nlm.nih.gov/Taxonomy/Browser/wwwtax.cgi?id=9362) | 75.0 | [7](https://blast.ncbi.nlm.nih.gov/Blast.cgi) |
| *.........*[*Sylvisorex ollula*](https://www.ncbi.nlm.nih.gov/Taxonomy/Browser/wwwtax.cgi?id=128151) | [insectivores](https://www.ncbi.nlm.nih.gov/Taxonomy/Browser/wwwtax.cgi?id=9362) | 75.0 | [1](https://blast.ncbi.nlm.nih.gov/Blast.cgi) |
| *.........*[*Crocidura nigripes*](https://www.ncbi.nlm.nih.gov/Taxonomy/Browser/wwwtax.cgi?id=61096) | [insectivores](https://www.ncbi.nlm.nih.gov/Taxonomy/Browser/wwwtax.cgi?id=9362) | 73.1 | [1](https://blast.ncbi.nlm.nih.gov/Blast.cgi) |
| *.........*[*Crocidura orientalis*](https://www.ncbi.nlm.nih.gov/Taxonomy/Browser/wwwtax.cgi?id=389337) | [insectivores](https://www.ncbi.nlm.nih.gov/Taxonomy/Browser/wwwtax.cgi?id=9362) | 73.1 | [1](https://blast.ncbi.nlm.nih.gov/Blast.cgi) |
| *.........*[*Crocidura orientalis lawuana*](https://www.ncbi.nlm.nih.gov/Taxonomy/Browser/wwwtax.cgi?id=458318) | [insectivores](https://www.ncbi.nlm.nih.gov/Taxonomy/Browser/wwwtax.cgi?id=9362) | 73.1 | [1](https://blast.ncbi.nlm.nih.gov/Blast.cgi) |
| *.........*[*Scutisorex congicus*](https://www.ncbi.nlm.nih.gov/Taxonomy/Browser/wwwtax.cgi?id=2899589) | [insectivores](https://www.ncbi.nlm.nih.gov/Taxonomy/Browser/wwwtax.cgi?id=9362) | 71.3 | [2](https://blast.ncbi.nlm.nih.gov/Blast.cgi) |
| *.........*[*Scutisorex somereni*](https://www.ncbi.nlm.nih.gov/Taxonomy/Browser/wwwtax.cgi?id=150088) | [insectivores](https://www.ncbi.nlm.nih.gov/Taxonomy/Browser/wwwtax.cgi?id=9362) | 71.3 | [2](https://blast.ncbi.nlm.nih.gov/Blast.cgi) |
| *.........*[*Crocidura yaldeni*](https://www.ncbi.nlm.nih.gov/Taxonomy/Browser/wwwtax.cgi?id=2794851) | [insectivores](https://www.ncbi.nlm.nih.gov/Taxonomy/Browser/wwwtax.cgi?id=9362) | 69.4 | [3](https://blast.ncbi.nlm.nih.gov/Blast.cgi) |
| *.........*[*Crocidura sp. S-165342*](https://www.ncbi.nlm.nih.gov/Taxonomy/Browser/wwwtax.cgi?id=561573) | [insectivores](https://www.ncbi.nlm.nih.gov/Taxonomy/Browser/wwwtax.cgi?id=9362) | 69.4 | [1](https://blast.ncbi.nlm.nih.gov/Blast.cgi) |
| *.........*[*Crocidura baluensis*](https://www.ncbi.nlm.nih.gov/Taxonomy/Browser/wwwtax.cgi?id=2821396) | [insectivores](https://www.ncbi.nlm.nih.gov/Taxonomy/Browser/wwwtax.cgi?id=9362) | 67.6 | [2](https://blast.ncbi.nlm.nih.gov/Blast.cgi) |
| *.........*[*Crocidura foetida doriae*](https://www.ncbi.nlm.nih.gov/Taxonomy/Browser/wwwtax.cgi?id=2821398) | [insectivores](https://www.ncbi.nlm.nih.gov/Taxonomy/Browser/wwwtax.cgi?id=9362) | 67.6 | [6](https://blast.ncbi.nlm.nih.gov/Blast.cgi) |
| *........*[*Neotetracus sinensis*](https://www.ncbi.nlm.nih.gov/Taxonomy/Browser/wwwtax.cgi?id=977878) | [insectivores](https://www.ncbi.nlm.nih.gov/Taxonomy/Browser/wwwtax.cgi?id=9362) | 82.4 | [3](https://blast.ncbi.nlm.nih.gov/Blast.cgi) |
| *.......*[*Ovis ammon hodgsoni*](https://www.ncbi.nlm.nih.gov/Taxonomy/Browser/wwwtax.cgi?id=439458) | [even-toed ungulates](https://www.ncbi.nlm.nih.gov/Taxonomy/Browser/wwwtax.cgi?id=91561) | 134 | [2](https://blast.ncbi.nlm.nih.gov/Blast.cgi) |
| *.......*[*Ovis ammon*](https://www.ncbi.nlm.nih.gov/Taxonomy/Browser/wwwtax.cgi?id=30527) | [even-toed ungulates](https://www.ncbi.nlm.nih.gov/Taxonomy/Browser/wwwtax.cgi?id=91561) | 134 | [28](https://blast.ncbi.nlm.nih.gov/Blast.cgi) |
| *.......*[*Ovis dalli*](https://www.ncbi.nlm.nih.gov/Taxonomy/Browser/wwwtax.cgi?id=9943) | [even-toed ungulates](https://www.ncbi.nlm.nih.gov/Taxonomy/Browser/wwwtax.cgi?id=91561) | 128 | [6](https://blast.ncbi.nlm.nih.gov/Blast.cgi) |
| *.......*[*Odocoileus virginianus*](https://www.ncbi.nlm.nih.gov/Taxonomy/Browser/wwwtax.cgi?id=9874) | [even-toed ungulates](https://www.ncbi.nlm.nih.gov/Taxonomy/Browser/wwwtax.cgi?id=91561) | 128 | [339](https://blast.ncbi.nlm.nih.gov/Blast.cgi) |
| *.......*[*Ovis ammon darwini*](https://www.ncbi.nlm.nih.gov/Taxonomy/Browser/wwwtax.cgi?id=72760) | [even-toed ungulates](https://www.ncbi.nlm.nih.gov/Taxonomy/Browser/wwwtax.cgi?id=91561) | 128 | [10](https://blast.ncbi.nlm.nih.gov/Blast.cgi) |
| *.......*[*Ovis aries*](https://www.ncbi.nlm.nih.gov/Taxonomy/Browser/wwwtax.cgi?id=9940) | [even-toed ungulates](https://www.ncbi.nlm.nih.gov/Taxonomy/Browser/wwwtax.cgi?id=91561) | 128 | [7](https://blast.ncbi.nlm.nih.gov/Blast.cgi) |
| *.......*[*Ovis dalli dalli*](https://www.ncbi.nlm.nih.gov/Taxonomy/Browser/wwwtax.cgi?id=72761) | [even-toed ungulates](https://www.ncbi.nlm.nih.gov/Taxonomy/Browser/wwwtax.cgi?id=91561) | 128 | [1](https://blast.ncbi.nlm.nih.gov/Blast.cgi) |
| *.......*[*Madoqua saltiana*](https://www.ncbi.nlm.nih.gov/Taxonomy/Browser/wwwtax.cgi?id=1027994) | [even-toed ungulates](https://www.ncbi.nlm.nih.gov/Taxonomy/Browser/wwwtax.cgi?id=91561) | 124 | [3](https://blast.ncbi.nlm.nih.gov/Blast.cgi) |
| *.......*[*Ovis ammon ammon*](https://www.ncbi.nlm.nih.gov/Taxonomy/Browser/wwwtax.cgi?id=156212) | [even-toed ungulates](https://www.ncbi.nlm.nih.gov/Taxonomy/Browser/wwwtax.cgi?id=91561) | 122 | [7](https://blast.ncbi.nlm.nih.gov/Blast.cgi) |
| *.......*[*Odocoileus pandora*](https://www.ncbi.nlm.nih.gov/Taxonomy/Browser/wwwtax.cgi?id=1380314) | [even-toed ungulates](https://www.ncbi.nlm.nih.gov/Taxonomy/Browser/wwwtax.cgi?id=91561) | 122 | [4](https://blast.ncbi.nlm.nih.gov/Blast.cgi) |
| *.......*[*Ovis ammon severtzovi*](https://www.ncbi.nlm.nih.gov/Taxonomy/Browser/wwwtax.cgi?id=509045) | [even-toed ungulates](https://www.ncbi.nlm.nih.gov/Taxonomy/Browser/wwwtax.cgi?id=91561) | 122 | [7](https://blast.ncbi.nlm.nih.gov/Blast.cgi) |
| *.......*[*Ovis ammon polii*](https://www.ncbi.nlm.nih.gov/Taxonomy/Browser/wwwtax.cgi?id=230172) | [even-toed ungulates](https://www.ncbi.nlm.nih.gov/Taxonomy/Browser/wwwtax.cgi?id=91561) | 122 | [12](https://blast.ncbi.nlm.nih.gov/Blast.cgi) |
| *.......*[*Pseudois nayaur*](https://www.ncbi.nlm.nih.gov/Taxonomy/Browser/wwwtax.cgi?id=59542) | [even-toed ungulates](https://www.ncbi.nlm.nih.gov/Taxonomy/Browser/wwwtax.cgi?id=91561) | 122 | [1](https://blast.ncbi.nlm.nih.gov/Blast.cgi) |
| *.......*[*Ovis ammon karelini*](https://www.ncbi.nlm.nih.gov/Taxonomy/Browser/wwwtax.cgi?id=230171) | [even-toed ungulates](https://www.ncbi.nlm.nih.gov/Taxonomy/Browser/wwwtax.cgi?id=91561) | 122 | [8](https://blast.ncbi.nlm.nih.gov/Blast.cgi) |
| *.......*[*Mazama americana*](https://www.ncbi.nlm.nih.gov/Taxonomy/Browser/wwwtax.cgi?id=43334) | [even-toed ungulates](https://www.ncbi.nlm.nih.gov/Taxonomy/Browser/wwwtax.cgi?id=91561) | 122 | [5](https://blast.ncbi.nlm.nih.gov/Blast.cgi) |
| *.......*[*Ovis orientalis*](https://www.ncbi.nlm.nih.gov/Taxonomy/Browser/wwwtax.cgi?id=469796) | [even-toed ungulates](https://www.ncbi.nlm.nih.gov/Taxonomy/Browser/wwwtax.cgi?id=91561) | 122 | [1](https://blast.ncbi.nlm.nih.gov/Blast.cgi) |
| *.......*[*Ovis vignei cycloceros*](https://www.ncbi.nlm.nih.gov/Taxonomy/Browser/wwwtax.cgi?id=509057) | [even-toed ungulates](https://www.ncbi.nlm.nih.gov/Taxonomy/Browser/wwwtax.cgi?id=91561) | 122 | [1](https://blast.ncbi.nlm.nih.gov/Blast.cgi) |
| *.......*[*Capra cylindricornis*](https://www.ncbi.nlm.nih.gov/Taxonomy/Browser/wwwtax.cgi?id=72541) | [even-toed ungulates](https://www.ncbi.nlm.nih.gov/Taxonomy/Browser/wwwtax.cgi?id=91561) | 122 | [1](https://blast.ncbi.nlm.nih.gov/Blast.cgi) |
| *.......*[*Odocoileus hemionus*](https://www.ncbi.nlm.nih.gov/Taxonomy/Browser/wwwtax.cgi?id=9872) | [even-toed ungulates](https://www.ncbi.nlm.nih.gov/Taxonomy/Browser/wwwtax.cgi?id=91561) | 121 | [131](https://blast.ncbi.nlm.nih.gov/Blast.cgi) |
| *.......*[*Capricornis crispus*](https://www.ncbi.nlm.nih.gov/Taxonomy/Browser/wwwtax.cgi?id=9966) | [even-toed ungulates](https://www.ncbi.nlm.nih.gov/Taxonomy/Browser/wwwtax.cgi?id=91561) | 117 | [10](https://blast.ncbi.nlm.nih.gov/Blast.cgi) |
| *.......*[*Mazama temama*](https://www.ncbi.nlm.nih.gov/Taxonomy/Browser/wwwtax.cgi?id=1380339) | [even-toed ungulates](https://www.ncbi.nlm.nih.gov/Taxonomy/Browser/wwwtax.cgi?id=91561) | 117 | [13](https://blast.ncbi.nlm.nih.gov/Blast.cgi) |
| *.......*[*Mazama nana*](https://www.ncbi.nlm.nih.gov/Taxonomy/Browser/wwwtax.cgi?id=397613) | [even-toed ungulates](https://www.ncbi.nlm.nih.gov/Taxonomy/Browser/wwwtax.cgi?id=91561) | 117 | [2](https://blast.ncbi.nlm.nih.gov/Blast.cgi) |
| *.......*[*Odocoileus hemionus hemionus*](https://www.ncbi.nlm.nih.gov/Taxonomy/Browser/wwwtax.cgi?id=9877) | [even-toed ungulates](https://www.ncbi.nlm.nih.gov/Taxonomy/Browser/wwwtax.cgi?id=91561) | 117 | [58](https://blast.ncbi.nlm.nih.gov/Blast.cgi) |
| *.......*[*Odocoileus hemionus californicus*](https://www.ncbi.nlm.nih.gov/Taxonomy/Browser/wwwtax.cgi?id=598490) | [even-toed ungulates](https://www.ncbi.nlm.nih.gov/Taxonomy/Browser/wwwtax.cgi?id=91561) | 117 | [17](https://blast.ncbi.nlm.nih.gov/Blast.cgi) |
| *.......*[*Odocoileus hemionus fuliginatus*](https://www.ncbi.nlm.nih.gov/Taxonomy/Browser/wwwtax.cgi?id=598493) | [even-toed ungulates](https://www.ncbi.nlm.nih.gov/Taxonomy/Browser/wwwtax.cgi?id=91561) | 117 | [9](https://blast.ncbi.nlm.nih.gov/Blast.cgi) |
| *.......*[*Odocoileus hemionus peninsulae*](https://www.ncbi.nlm.nih.gov/Taxonomy/Browser/wwwtax.cgi?id=598489) | [even-toed ungulates](https://www.ncbi.nlm.nih.gov/Taxonomy/Browser/wwwtax.cgi?id=91561) | 117 | [6](https://blast.ncbi.nlm.nih.gov/Blast.cgi) |
| *.......*[*Odocoileus hemionus crooki*](https://www.ncbi.nlm.nih.gov/Taxonomy/Browser/wwwtax.cgi?id=9876) | [even-toed ungulates](https://www.ncbi.nlm.nih.gov/Taxonomy/Browser/wwwtax.cgi?id=91561) | 117 | [25](https://blast.ncbi.nlm.nih.gov/Blast.cgi) |
| *.......*[*Mazama sp.*](https://www.ncbi.nlm.nih.gov/Taxonomy/Browser/wwwtax.cgi?id=74479) | [even-toed ungulates](https://www.ncbi.nlm.nih.gov/Taxonomy/Browser/wwwtax.cgi?id=91561) | 117 | [1](https://blast.ncbi.nlm.nih.gov/Blast.cgi) |
| *.......*[*Raphicerus sharpei*](https://www.ncbi.nlm.nih.gov/Taxonomy/Browser/wwwtax.cgi?id=66436) | [even-toed ungulates](https://www.ncbi.nlm.nih.gov/Taxonomy/Browser/wwwtax.cgi?id=91561) | 117 | [2](https://blast.ncbi.nlm.nih.gov/Blast.cgi) |
| *.......*[*Capra hircus*](https://www.ncbi.nlm.nih.gov/Taxonomy/Browser/wwwtax.cgi?id=9925) | [even-toed ungulates](https://www.ncbi.nlm.nih.gov/Taxonomy/Browser/wwwtax.cgi?id=91561) | 111 | [2](https://blast.ncbi.nlm.nih.gov/Blast.cgi) |
| *.......*[*Plecotus ognevi*](https://www.ncbi.nlm.nih.gov/Taxonomy/Browser/wwwtax.cgi?id=360160) | [bats](https://www.ncbi.nlm.nih.gov/Taxonomy/Browser/wwwtax.cgi?id=9397) | 111 | [8](https://blast.ncbi.nlm.nih.gov/Blast.cgi) |
| *.......*[*Mazama bororo*](https://www.ncbi.nlm.nih.gov/Taxonomy/Browser/wwwtax.cgi?id=397615) | [even-toed ungulates](https://www.ncbi.nlm.nih.gov/Taxonomy/Browser/wwwtax.cgi?id=91561) | 111 | [2](https://blast.ncbi.nlm.nih.gov/Blast.cgi) |
| *.......*[*Plecotus auritus*](https://www.ncbi.nlm.nih.gov/Taxonomy/Browser/wwwtax.cgi?id=61862) | [bats](https://www.ncbi.nlm.nih.gov/Taxonomy/Browser/wwwtax.cgi?id=9397) | 111 | [2](https://blast.ncbi.nlm.nih.gov/Blast.cgi) |
| *.......*[*Odocoileus hemionus sitkensis*](https://www.ncbi.nlm.nih.gov/Taxonomy/Browser/wwwtax.cgi?id=9878) | [even-toed ungulates](https://www.ncbi.nlm.nih.gov/Taxonomy/Browser/wwwtax.cgi?id=91561) | 111 | [6](https://blast.ncbi.nlm.nih.gov/Blast.cgi) |
| *.......*[*Odocoileus hemionus columbianus*](https://www.ncbi.nlm.nih.gov/Taxonomy/Browser/wwwtax.cgi?id=9873) | [even-toed ungulates](https://www.ncbi.nlm.nih.gov/Taxonomy/Browser/wwwtax.cgi?id=91561) | 111 | [41](https://blast.ncbi.nlm.nih.gov/Blast.cgi) |
| *.......*[*Odocoileus hemionus inyoensis*](https://www.ncbi.nlm.nih.gov/Taxonomy/Browser/wwwtax.cgi?id=598491) | [even-toed ungulates](https://www.ncbi.nlm.nih.gov/Taxonomy/Browser/wwwtax.cgi?id=91561) | 111 | [1](https://blast.ncbi.nlm.nih.gov/Blast.cgi) |
| *.......*[*Uroderma bilobatum*](https://www.ncbi.nlm.nih.gov/Taxonomy/Browser/wwwtax.cgi?id=27663) | [bats](https://www.ncbi.nlm.nih.gov/Taxonomy/Browser/wwwtax.cgi?id=9397) | 111 | [60](https://blast.ncbi.nlm.nih.gov/Blast.cgi) |
| *.......*[*Mazama rufina*](https://www.ncbi.nlm.nih.gov/Taxonomy/Browser/wwwtax.cgi?id=1088028) | [even-toed ungulates](https://www.ncbi.nlm.nih.gov/Taxonomy/Browser/wwwtax.cgi?id=91561) | 110 | [1](https://blast.ncbi.nlm.nih.gov/Blast.cgi) |
| *.......*[*Lophostoma silvicolum occidentalis*](https://www.ncbi.nlm.nih.gov/Taxonomy/Browser/wwwtax.cgi?id=1038732) | [bats](https://www.ncbi.nlm.nih.gov/Taxonomy/Browser/wwwtax.cgi?id=9397) | 110 | [3](https://blast.ncbi.nlm.nih.gov/Blast.cgi) |
| *.......*[*Odocoileus virginianus clavium*](https://www.ncbi.nlm.nih.gov/Taxonomy/Browser/wwwtax.cgi?id=1844112) | [even-toed ungulates](https://www.ncbi.nlm.nih.gov/Taxonomy/Browser/wwwtax.cgi?id=91561) | 106 | [36](https://blast.ncbi.nlm.nih.gov/Blast.cgi) |
| *.......*[*Odocoileus hemionus sheldoni*](https://www.ncbi.nlm.nih.gov/Taxonomy/Browser/wwwtax.cgi?id=598492) | [even-toed ungulates](https://www.ncbi.nlm.nih.gov/Taxonomy/Browser/wwwtax.cgi?id=91561) | 106 | [2](https://blast.ncbi.nlm.nih.gov/Blast.cgi) |
| *.......*[*Odocoileus hemionus eremicus*](https://www.ncbi.nlm.nih.gov/Taxonomy/Browser/wwwtax.cgi?id=598488) | [even-toed ungulates](https://www.ncbi.nlm.nih.gov/Taxonomy/Browser/wwwtax.cgi?id=91561) | 106 | [4](https://blast.ncbi.nlm.nih.gov/Blast.cgi) |
| *.......*[*Carollia brevicauda*](https://www.ncbi.nlm.nih.gov/Taxonomy/Browser/wwwtax.cgi?id=138695) | [bats](https://www.ncbi.nlm.nih.gov/Taxonomy/Browser/wwwtax.cgi?id=9397) | 106 | [1](https://blast.ncbi.nlm.nih.gov/Blast.cgi) |
| *.......*[*Hyemoschus aquaticus*](https://www.ncbi.nlm.nih.gov/Taxonomy/Browser/wwwtax.cgi?id=666915) | [even-toed ungulates](https://www.ncbi.nlm.nih.gov/Taxonomy/Browser/wwwtax.cgi?id=91561) | 104 | [2](https://blast.ncbi.nlm.nih.gov/Blast.cgi) |
| *.......*[*Lophostoma brasiliense*](https://www.ncbi.nlm.nih.gov/Taxonomy/Browser/wwwtax.cgi?id=409031) | [bats](https://www.ncbi.nlm.nih.gov/Taxonomy/Browser/wwwtax.cgi?id=9397) | 104 | [3](https://blast.ncbi.nlm.nih.gov/Blast.cgi) |
| *.......*[*Cephalophula zebra*](https://www.ncbi.nlm.nih.gov/Taxonomy/Browser/wwwtax.cgi?id=129233) | [even-toed ungulates](https://www.ncbi.nlm.nih.gov/Taxonomy/Browser/wwwtax.cgi?id=91561) | 104 | [1](https://blast.ncbi.nlm.nih.gov/Blast.cgi) |
| *.......*[*Miniopterus mossambicus*](https://www.ncbi.nlm.nih.gov/Taxonomy/Browser/wwwtax.cgi?id=1434095) | [bats](https://www.ncbi.nlm.nih.gov/Taxonomy/Browser/wwwtax.cgi?id=9397) | 102 | [13](https://blast.ncbi.nlm.nih.gov/Blast.cgi) |
| *.......*[*Sturnira oporaphilum*](https://www.ncbi.nlm.nih.gov/Taxonomy/Browser/wwwtax.cgi?id=192410) | [bats](https://www.ncbi.nlm.nih.gov/Taxonomy/Browser/wwwtax.cgi?id=9397) | 102 | [7](https://blast.ncbi.nlm.nih.gov/Blast.cgi) |
| *.......*[*Sturnira lilium*](https://www.ncbi.nlm.nih.gov/Taxonomy/Browser/wwwtax.cgi?id=27660) | [bats](https://www.ncbi.nlm.nih.gov/Taxonomy/Browser/wwwtax.cgi?id=9397) | 102 | [1](https://blast.ncbi.nlm.nih.gov/Blast.cgi) |
| *.......*[*Glischropus aquilus*](https://www.ncbi.nlm.nih.gov/Taxonomy/Browser/wwwtax.cgi?id=1699683) | [bats](https://www.ncbi.nlm.nih.gov/Taxonomy/Browser/wwwtax.cgi?id=9397) | 100 | [1](https://blast.ncbi.nlm.nih.gov/Blast.cgi) |
| *.......*[*Sturnira ludovici*](https://www.ncbi.nlm.nih.gov/Taxonomy/Browser/wwwtax.cgi?id=192405) | [bats](https://www.ncbi.nlm.nih.gov/Taxonomy/Browser/wwwtax.cgi?id=9397) | 100 | [4](https://blast.ncbi.nlm.nih.gov/Blast.cgi) |
| *.......*[*Craseonycteris thonglongyai*](https://www.ncbi.nlm.nih.gov/Taxonomy/Browser/wwwtax.cgi?id=208972) | [bats](https://www.ncbi.nlm.nih.gov/Taxonomy/Browser/wwwtax.cgi?id=9397) | 100 | [4](https://blast.ncbi.nlm.nih.gov/Blast.cgi) |
| *.......*[*Tonatia bidens*](https://www.ncbi.nlm.nih.gov/Taxonomy/Browser/wwwtax.cgi?id=9426) | [bats](https://www.ncbi.nlm.nih.gov/Taxonomy/Browser/wwwtax.cgi?id=9397) | 99.0 | [4](https://blast.ncbi.nlm.nih.gov/Blast.cgi) |
| *.......*[*Spilogale interrupta*](https://www.ncbi.nlm.nih.gov/Taxonomy/Browser/wwwtax.cgi?id=2340998) | [carnivores](https://www.ncbi.nlm.nih.gov/Taxonomy/Browser/wwwtax.cgi?id=33554) | 95.3 | [34](https://blast.ncbi.nlm.nih.gov/Blast.cgi) |
| *.......*[*Glauconycteris beatrix*](https://www.ncbi.nlm.nih.gov/Taxonomy/Browser/wwwtax.cgi?id=177182) | [bats](https://www.ncbi.nlm.nih.gov/Taxonomy/Browser/wwwtax.cgi?id=9397) | 95.3 | [12](https://blast.ncbi.nlm.nih.gov/Blast.cgi) |
| *.......*[*Scotophilus heathii*](https://www.ncbi.nlm.nih.gov/Taxonomy/Browser/wwwtax.cgi?id=159339) | [bats](https://www.ncbi.nlm.nih.gov/Taxonomy/Browser/wwwtax.cgi?id=9397) | 95.3 | [9](https://blast.ncbi.nlm.nih.gov/Blast.cgi) |
| *.......*[*Histiotus montanus*](https://www.ncbi.nlm.nih.gov/Taxonomy/Browser/wwwtax.cgi?id=1161940) | [bats](https://www.ncbi.nlm.nih.gov/Taxonomy/Browser/wwwtax.cgi?id=9397) | 93.5 | [6](https://blast.ncbi.nlm.nih.gov/Blast.cgi) |
| *.......*[*Histiotus macrotus*](https://www.ncbi.nlm.nih.gov/Taxonomy/Browser/wwwtax.cgi?id=258919) | [bats](https://www.ncbi.nlm.nih.gov/Taxonomy/Browser/wwwtax.cgi?id=9397) | 93.5 | [18](https://blast.ncbi.nlm.nih.gov/Blast.cgi) |
| *.......*[*Glauconycteris egeria*](https://www.ncbi.nlm.nih.gov/Taxonomy/Browser/wwwtax.cgi?id=909368) | [bats](https://www.ncbi.nlm.nih.gov/Taxonomy/Browser/wwwtax.cgi?id=9397) | 93.5 | [7](https://blast.ncbi.nlm.nih.gov/Blast.cgi) |
| *.......*[*Spilogale putorius*](https://www.ncbi.nlm.nih.gov/Taxonomy/Browser/wwwtax.cgi?id=30552) | [carnivores](https://www.ncbi.nlm.nih.gov/Taxonomy/Browser/wwwtax.cgi?id=33554) | 93.5 | [1](https://blast.ncbi.nlm.nih.gov/Blast.cgi) |
| *.......*[*Tylonycteris malayana*](https://www.ncbi.nlm.nih.gov/Taxonomy/Browser/wwwtax.cgi?id=1964257) | [bats](https://www.ncbi.nlm.nih.gov/Taxonomy/Browser/wwwtax.cgi?id=9397) | 93.5 | [3](https://blast.ncbi.nlm.nih.gov/Blast.cgi) |
| *.......*[*Tonatia saurophila maresi*](https://www.ncbi.nlm.nih.gov/Taxonomy/Browser/wwwtax.cgi?id=2715937) | [bats](https://www.ncbi.nlm.nih.gov/Taxonomy/Browser/wwwtax.cgi?id=9397) | 91.6 | [2](https://blast.ncbi.nlm.nih.gov/Blast.cgi) |
| *.......*[*Nycteris macrotis complex sp. 1 TD-2019*](https://www.ncbi.nlm.nih.gov/Taxonomy/Browser/wwwtax.cgi?id=2596771) | [bats](https://www.ncbi.nlm.nih.gov/Taxonomy/Browser/wwwtax.cgi?id=9397) | 91.6 | [1](https://blast.ncbi.nlm.nih.gov/Blast.cgi) |
| *.......*[*Glauconycteris poensis*](https://www.ncbi.nlm.nih.gov/Taxonomy/Browser/wwwtax.cgi?id=258909) | [bats](https://www.ncbi.nlm.nih.gov/Taxonomy/Browser/wwwtax.cgi?id=9397) | 91.6 | [3](https://blast.ncbi.nlm.nih.gov/Blast.cgi) |
| *.......*[*Myotis cf. simus*](https://www.ncbi.nlm.nih.gov/Taxonomy/Browser/wwwtax.cgi?id=1239428) | [bats](https://www.ncbi.nlm.nih.gov/Taxonomy/Browser/wwwtax.cgi?id=9397) | 91.6 | [1](https://blast.ncbi.nlm.nih.gov/Blast.cgi) |
| *.......*[*Sturnira magna*](https://www.ncbi.nlm.nih.gov/Taxonomy/Browser/wwwtax.cgi?id=192407) | [bats](https://www.ncbi.nlm.nih.gov/Taxonomy/Browser/wwwtax.cgi?id=9397) | 89.8 | [5](https://blast.ncbi.nlm.nih.gov/Blast.cgi) |
| *.......*[*Sturnira bogotensis*](https://www.ncbi.nlm.nih.gov/Taxonomy/Browser/wwwtax.cgi?id=192402) | [bats](https://www.ncbi.nlm.nih.gov/Taxonomy/Browser/wwwtax.cgi?id=9397) | 89.8 | [5](https://blast.ncbi.nlm.nih.gov/Blast.cgi) |
| *.......*[*Scotophilus marovaza*](https://www.ncbi.nlm.nih.gov/Taxonomy/Browser/wwwtax.cgi?id=565071) | [bats](https://www.ncbi.nlm.nih.gov/Taxonomy/Browser/wwwtax.cgi?id=9397) | 89.8 | [2](https://blast.ncbi.nlm.nih.gov/Blast.cgi) |
| *.......*[*Scotophilus tandrefana*](https://www.ncbi.nlm.nih.gov/Taxonomy/Browser/wwwtax.cgi?id=565070) | [bats](https://www.ncbi.nlm.nih.gov/Taxonomy/Browser/wwwtax.cgi?id=9397) | 89.8 | [1](https://blast.ncbi.nlm.nih.gov/Blast.cgi) |
| *.......Eptesicus sp.* AMNH M-278524 | [bats](https://www.ncbi.nlm.nih.gov/Taxonomy/Browser/wwwtax.cgi?id=9397) | 87.9 | [1](https://blast.ncbi.nlm.nih.gov/Blast.cgi) |
| *.......*[*Scotophilus viridis nigritellus*](https://www.ncbi.nlm.nih.gov/Taxonomy/Browser/wwwtax.cgi?id=565069) | [bats](https://www.ncbi.nlm.nih.gov/Taxonomy/Browser/wwwtax.cgi?id=9397) | 87.9 | [2](https://blast.ncbi.nlm.nih.gov/Blast.cgi) |
| *.......Eptesicus sp.* Santa Fe195 | [bats](https://www.ncbi.nlm.nih.gov/Taxonomy/Browser/wwwtax.cgi?id=9397) | 87.9 | [1](https://blast.ncbi.nlm.nih.gov/Blast.cgi) |
| *.......Eptesicus sp.* Culul142 | [bats](https://www.ncbi.nlm.nih.gov/Taxonomy/Browser/wwwtax.cgi?id=9397) | 87.9 | [1](https://blast.ncbi.nlm.nih.gov/Blast.cgi) |
| *.......Eptesicus sp.* Culul140 | [bats](https://www.ncbi.nlm.nih.gov/Taxonomy/Browser/wwwtax.cgi?id=9397) | 87.9 | [1](https://blast.ncbi.nlm.nih.gov/Blast.cgi) |
| *.......Eptesicus sp.* Culul134 | [bats](https://www.ncbi.nlm.nih.gov/Taxonomy/Browser/wwwtax.cgi?id=9397) | 87.9 | [1](https://blast.ncbi.nlm.nih.gov/Blast.cgi) |
| *.......*[*Nycticeius humeralis*](https://www.ncbi.nlm.nih.gov/Taxonomy/Browser/wwwtax.cgi?id=27670) | [bats](https://www.ncbi.nlm.nih.gov/Taxonomy/Browser/wwwtax.cgi?id=9397) | 82.4 | [1](https://blast.ncbi.nlm.nih.gov/Blast.cgi) |
| *.......*[*Pteronotus davyi*](https://www.ncbi.nlm.nih.gov/Taxonomy/Browser/wwwtax.cgi?id=94956) | [bats](https://www.ncbi.nlm.nih.gov/Taxonomy/Browser/wwwtax.cgi?id=9397) | 82.4 | [1](https://blast.ncbi.nlm.nih.gov/Blast.cgi) |
| *.......*[*Vampyrum spectrum*](https://www.ncbi.nlm.nih.gov/Taxonomy/Browser/wwwtax.cgi?id=148074) | [bats](https://www.ncbi.nlm.nih.gov/Taxonomy/Browser/wwwtax.cgi?id=9397) | 76.8 | [1](https://blast.ncbi.nlm.nih.gov/Blast.cgi) |
| *.......*[*Glauconycteris alboguttata*](https://www.ncbi.nlm.nih.gov/Taxonomy/Browser/wwwtax.cgi?id=2093328) | [bats](https://www.ncbi.nlm.nih.gov/Taxonomy/Browser/wwwtax.cgi?id=9397) | 71.3 | [12](https://blast.ncbi.nlm.nih.gov/Blast.cgi) |
| *......*[*Eospalax rufescens*](https://www.ncbi.nlm.nih.gov/Taxonomy/Browser/wwwtax.cgi?id=146137) | [rodents](https://www.ncbi.nlm.nih.gov/Taxonomy/Browser/wwwtax.cgi?id=9989) | 126 | [3](https://blast.ncbi.nlm.nih.gov/Blast.cgi) |
| *......*[*Caprolagus hispidus*](https://www.ncbi.nlm.nih.gov/Taxonomy/Browser/wwwtax.cgi?id=235649) | [rabbits & hares](https://www.ncbi.nlm.nih.gov/Taxonomy/Browser/wwwtax.cgi?id=9975) | 122 | [1](https://blast.ncbi.nlm.nih.gov/Blast.cgi) |
| *......*[*Galea musteloides*](https://www.ncbi.nlm.nih.gov/Taxonomy/Browser/wwwtax.cgi?id=10146) | [rodents](https://www.ncbi.nlm.nih.gov/Taxonomy/Browser/wwwtax.cgi?id=9989) | 115 | [10](https://blast.ncbi.nlm.nih.gov/Blast.cgi) |
| *......*[*Lepus hainanus*](https://www.ncbi.nlm.nih.gov/Taxonomy/Browser/wwwtax.cgi?id=274686) | [rabbits & hares](https://www.ncbi.nlm.nih.gov/Taxonomy/Browser/wwwtax.cgi?id=9975) | 110 | [2](https://blast.ncbi.nlm.nih.gov/Blast.cgi) |
| *......*[*Notocitellus annulatus*](https://www.ncbi.nlm.nih.gov/Taxonomy/Browser/wwwtax.cgi?id=99831) | [rodents](https://www.ncbi.nlm.nih.gov/Taxonomy/Browser/wwwtax.cgi?id=9989) | 110 | [2](https://blast.ncbi.nlm.nih.gov/Blast.cgi) |
| *......*[*Dipus sagitta*](https://www.ncbi.nlm.nih.gov/Taxonomy/Browser/wwwtax.cgi?id=73863) | [rodents](https://www.ncbi.nlm.nih.gov/Taxonomy/Browser/wwwtax.cgi?id=9989) | 106 | [70](https://blast.ncbi.nlm.nih.gov/Blast.cgi) |
| *......*[*Eospalax fontanierii baileyi*](https://www.ncbi.nlm.nih.gov/Taxonomy/Browser/wwwtax.cgi?id=146132) | [rodents](https://www.ncbi.nlm.nih.gov/Taxonomy/Browser/wwwtax.cgi?id=9989) | 104 | [29](https://blast.ncbi.nlm.nih.gov/Blast.cgi) |
| *......Niviventer sp.* 1 LL-2013 | [rodents](https://www.ncbi.nlm.nih.gov/Taxonomy/Browser/wwwtax.cgi?id=9989) | 100 | [7](https://blast.ncbi.nlm.nih.gov/Blast.cgi) |
| *......*[*Niviventer eha*](https://www.ncbi.nlm.nih.gov/Taxonomy/Browser/wwwtax.cgi?id=425033) | [rodents](https://www.ncbi.nlm.nih.gov/Taxonomy/Browser/wwwtax.cgi?id=9989) | 100 | [10](https://blast.ncbi.nlm.nih.gov/Blast.cgi) |
| *......*[*Microtus obscurus*](https://www.ncbi.nlm.nih.gov/Taxonomy/Browser/wwwtax.cgi?id=523745) | [rodents](https://www.ncbi.nlm.nih.gov/Taxonomy/Browser/wwwtax.cgi?id=9989) | 99.0 | [1](https://blast.ncbi.nlm.nih.gov/Blast.cgi) |
| *......*[*Apodemus draco*](https://www.ncbi.nlm.nih.gov/Taxonomy/Browser/wwwtax.cgi?id=129247) | [rodents](https://www.ncbi.nlm.nih.gov/Taxonomy/Browser/wwwtax.cgi?id=9989) | 97.1 | [24](https://blast.ncbi.nlm.nih.gov/Blast.cgi) |
| *......*[*Niviventer confucianus*](https://www.ncbi.nlm.nih.gov/Taxonomy/Browser/wwwtax.cgi?id=248811) | [rodents](https://www.ncbi.nlm.nih.gov/Taxonomy/Browser/wwwtax.cgi?id=9989) | 97.1 | [1](https://blast.ncbi.nlm.nih.gov/Blast.cgi) |
| *......*[*Cheracebus lugens*](https://www.ncbi.nlm.nih.gov/Taxonomy/Browser/wwwtax.cgi?id=210166) | [primates](https://www.ncbi.nlm.nih.gov/Taxonomy/Browser/wwwtax.cgi?id=9443) | 97.1 | [41](https://blast.ncbi.nlm.nih.gov/Blast.cgi) |
| *......*[*Niviventer cremoriventer*](https://www.ncbi.nlm.nih.gov/Taxonomy/Browser/wwwtax.cgi?id=69083) | [rodents](https://www.ncbi.nlm.nih.gov/Taxonomy/Browser/wwwtax.cgi?id=9989) | 95.3 | [1](https://blast.ncbi.nlm.nih.gov/Blast.cgi) |
| *......*[*Niviventer brahma*](https://www.ncbi.nlm.nih.gov/Taxonomy/Browser/wwwtax.cgi?id=1925727) | [rodents](https://www.ncbi.nlm.nih.gov/Taxonomy/Browser/wwwtax.cgi?id=9989) | 95.3 | [2](https://blast.ncbi.nlm.nih.gov/Blast.cgi) |
| *......*[*Microcebus murinus*](https://www.ncbi.nlm.nih.gov/Taxonomy/Browser/wwwtax.cgi?id=30608) | [primates](https://www.ncbi.nlm.nih.gov/Taxonomy/Browser/wwwtax.cgi?id=9443) | 95.3 | [34](https://blast.ncbi.nlm.nih.gov/Blast.cgi) |
| *......*[*Hystrix africaeaustralis*](https://www.ncbi.nlm.nih.gov/Taxonomy/Browser/wwwtax.cgi?id=10138) | [rodents](https://www.ncbi.nlm.nih.gov/Taxonomy/Browser/wwwtax.cgi?id=9989) | 95.3 | [1](https://blast.ncbi.nlm.nih.gov/Blast.cgi) |
| *......*[*Glirulus japonicus*](https://www.ncbi.nlm.nih.gov/Taxonomy/Browser/wwwtax.cgi?id=55147) | [rodents](https://www.ncbi.nlm.nih.gov/Taxonomy/Browser/wwwtax.cgi?id=9989) | 93.5 | [8](https://blast.ncbi.nlm.nih.gov/Blast.cgi) |
| *......*[*Grammomys sp.*](https://www.ncbi.nlm.nih.gov/Taxonomy/Browser/wwwtax.cgi?id=121589) | [rodents](https://www.ncbi.nlm.nih.gov/Taxonomy/Browser/wwwtax.cgi?id=9989) | 91.6 | [19](https://blast.ncbi.nlm.nih.gov/Blast.cgi) |
| *......*[*Dipus sagitta bulganensis*](https://www.ncbi.nlm.nih.gov/Taxonomy/Browser/wwwtax.cgi?id=2171710) | [rodents](https://www.ncbi.nlm.nih.gov/Taxonomy/Browser/wwwtax.cgi?id=9989) | 91.6 | [1](https://blast.ncbi.nlm.nih.gov/Blast.cgi) |
| *......*[*Steatomys krebsii*](https://www.ncbi.nlm.nih.gov/Taxonomy/Browser/wwwtax.cgi?id=1392804) | [rodents](https://www.ncbi.nlm.nih.gov/Taxonomy/Browser/wwwtax.cgi?id=9989) | 91.6 | [1](https://blast.ncbi.nlm.nih.gov/Blast.cgi) |
| *......*[*Grammomys surdaster*](https://www.ncbi.nlm.nih.gov/Taxonomy/Browser/wwwtax.cgi?id=491861) | [rodents](https://www.ncbi.nlm.nih.gov/Taxonomy/Browser/wwwtax.cgi?id=9989) | 91.6 | [11](https://blast.ncbi.nlm.nih.gov/Blast.cgi) |
| *......*[*Hystrix brachyura*](https://www.ncbi.nlm.nih.gov/Taxonomy/Browser/wwwtax.cgi?id=143286) | [rodents](https://www.ncbi.nlm.nih.gov/Taxonomy/Browser/wwwtax.cgi?id=9989) | 91.6 | [2](https://blast.ncbi.nlm.nih.gov/Blast.cgi) |
| *......*[*Hystrix indica*](https://www.ncbi.nlm.nih.gov/Taxonomy/Browser/wwwtax.cgi?id=192883) | [rodents](https://www.ncbi.nlm.nih.gov/Taxonomy/Browser/wwwtax.cgi?id=9989) | 89.8 | [3](https://blast.ncbi.nlm.nih.gov/Blast.cgi) |
| *......*[*Cheracebus torquatus*](https://www.ncbi.nlm.nih.gov/Taxonomy/Browser/wwwtax.cgi?id=30592) | [primates](https://www.ncbi.nlm.nih.gov/Taxonomy/Browser/wwwtax.cgi?id=9443) | 89.8 | [4](https://blast.ncbi.nlm.nih.gov/Blast.cgi) |
| *......*[*Tachyoryctes macrocephalus*](https://www.ncbi.nlm.nih.gov/Taxonomy/Browser/wwwtax.cgi?id=2170709) | [rodents](https://www.ncbi.nlm.nih.gov/Taxonomy/Browser/wwwtax.cgi?id=9989) | 89.8 | [1](https://blast.ncbi.nlm.nih.gov/Blast.cgi) |
| *......Dendromus sp. indet.* 6 GV-2021 | [rodents](https://www.ncbi.nlm.nih.gov/Taxonomy/Browser/wwwtax.cgi?id=9989) | 89.8 | [3](https://blast.ncbi.nlm.nih.gov/Blast.cgi) |
| *......Dendromus sp. indet. 1*0 GV-2021 | [rodents](https://www.ncbi.nlm.nih.gov/Taxonomy/Browser/wwwtax.cgi?id=9989) | 89.8 | [1](https://blast.ncbi.nlm.nih.gov/Blast.cgi) |
| *......*[*Otomys occidentalis*](https://www.ncbi.nlm.nih.gov/Taxonomy/Browser/wwwtax.cgi?id=190451) | [rodents](https://www.ncbi.nlm.nih.gov/Taxonomy/Browser/wwwtax.cgi?id=9989) | 89.8 | [1](https://blast.ncbi.nlm.nih.gov/Blast.cgi) |
| *......*[*Fukomys sp. PAVD-2007*](https://www.ncbi.nlm.nih.gov/Taxonomy/Browser/wwwtax.cgi?id=423611) | [rodents](https://www.ncbi.nlm.nih.gov/Taxonomy/Browser/wwwtax.cgi?id=9989) | 89.8 | [1](https://blast.ncbi.nlm.nih.gov/Blast.cgi) |
| *......*[*Jaculus blanfordi*](https://www.ncbi.nlm.nih.gov/Taxonomy/Browser/wwwtax.cgi?id=1041418) | [rodents](https://www.ncbi.nlm.nih.gov/Taxonomy/Browser/wwwtax.cgi?id=9989) | 87.9 | [16](https://blast.ncbi.nlm.nih.gov/Blast.cgi) |
| *......*[*Apodemus nigrus*](https://www.ncbi.nlm.nih.gov/Taxonomy/Browser/wwwtax.cgi?id=2602584) | [rodents](https://www.ncbi.nlm.nih.gov/Taxonomy/Browser/wwwtax.cgi?id=9989) | 87.9 | [5](https://blast.ncbi.nlm.nih.gov/Blast.cgi) |
| *......*[*Pithecia albicans*](https://www.ncbi.nlm.nih.gov/Taxonomy/Browser/wwwtax.cgi?id=2946514) | [primates](https://www.ncbi.nlm.nih.gov/Taxonomy/Browser/wwwtax.cgi?id=9443) | 87.9 | [2](https://blast.ncbi.nlm.nih.gov/Blast.cgi) |
| *......Graphiurus sp.* ETH1315 | [rodents](https://www.ncbi.nlm.nih.gov/Taxonomy/Browser/wwwtax.cgi?id=9989) | 87.9 | [1](https://blast.ncbi.nlm.nih.gov/Blast.cgi) |
| *......Niviventer sp.* 2 MP-2010 | [rodents](https://www.ncbi.nlm.nih.gov/Taxonomy/Browser/wwwtax.cgi?id=9989) | 87.9 | [1](https://blast.ncbi.nlm.nih.gov/Blast.cgi) |
| *......*[*Fukomys whytei*](https://www.ncbi.nlm.nih.gov/Taxonomy/Browser/wwwtax.cgi?id=423609) | [rodents](https://www.ncbi.nlm.nih.gov/Taxonomy/Browser/wwwtax.cgi?id=9989) | 86.1 | [7](https://blast.ncbi.nlm.nih.gov/Blast.cgi) |
| *......*[*Callicebus personatus*](https://www.ncbi.nlm.nih.gov/Taxonomy/Browser/wwwtax.cgi?id=70814) | [primates](https://www.ncbi.nlm.nih.gov/Taxonomy/Browser/wwwtax.cgi?id=9443) | 86.1 | [2](https://blast.ncbi.nlm.nih.gov/Blast.cgi) |
| *......*[*Heliophobius argenteocinereus*](https://www.ncbi.nlm.nih.gov/Taxonomy/Browser/wwwtax.cgi?id=10179) | [rodents](https://www.ncbi.nlm.nih.gov/Taxonomy/Browser/wwwtax.cgi?id=9989) | 86.1 | [26](https://blast.ncbi.nlm.nih.gov/Blast.cgi) |
| *......*[*Cheracebus lucifer*](https://www.ncbi.nlm.nih.gov/Taxonomy/Browser/wwwtax.cgi?id=2487712) | [primates](https://www.ncbi.nlm.nih.gov/Taxonomy/Browser/wwwtax.cgi?id=9443) | 86.1 | [11](https://blast.ncbi.nlm.nih.gov/Blast.cgi) |
| *......*[*Arborimus longicaudus*](https://www.ncbi.nlm.nih.gov/Taxonomy/Browser/wwwtax.cgi?id=251274) | [rodents](https://www.ncbi.nlm.nih.gov/Taxonomy/Browser/wwwtax.cgi?id=9989) | 84.2 | [1](https://blast.ncbi.nlm.nih.gov/Blast.cgi) |
| *......*[*Heliophobius emini*](https://www.ncbi.nlm.nih.gov/Taxonomy/Browser/wwwtax.cgi?id=1830327) | [rodents](https://www.ncbi.nlm.nih.gov/Taxonomy/Browser/wwwtax.cgi?id=9989) | 84.2 | [2](https://blast.ncbi.nlm.nih.gov/Blast.cgi) |
| *......*[*Jaculus jaculus*](https://www.ncbi.nlm.nih.gov/Taxonomy/Browser/wwwtax.cgi?id=51337) | [rodents](https://www.ncbi.nlm.nih.gov/Taxonomy/Browser/wwwtax.cgi?id=9989) | 84.2 | [16](https://blast.ncbi.nlm.nih.gov/Blast.cgi) |
| *......Graphiurus sp.* ETH1341 | [rodents](https://www.ncbi.nlm.nih.gov/Taxonomy/Browser/wwwtax.cgi?id=9989) | 84.2 | [1](https://blast.ncbi.nlm.nih.gov/Blast.cgi) |
| *......Graphiurus sp.* ETH1314 | [rodents](https://www.ncbi.nlm.nih.gov/Taxonomy/Browser/wwwtax.cgi?id=9989) | 84.2 | [1](https://blast.ncbi.nlm.nih.gov/Blast.cgi) |
| *......Graphiurus sp.* ETH0501 | [rodents](https://www.ncbi.nlm.nih.gov/Taxonomy/Browser/wwwtax.cgi?id=9989) | 84.2 | [1](https://blast.ncbi.nlm.nih.gov/Blast.cgi) |
| *......Dendromus sp. indet.* 7 GV-2021 | [rodents](https://www.ncbi.nlm.nih.gov/Taxonomy/Browser/wwwtax.cgi?id=9989) | 84.2 | [2](https://blast.ncbi.nlm.nih.gov/Blast.cgi) |
| *......Heliophobius sp.* CGF-2011a | [rodents](https://www.ncbi.nlm.nih.gov/Taxonomy/Browser/wwwtax.cgi?id=9989) | 84.2 | [4](https://blast.ncbi.nlm.nih.gov/Blast.cgi) |
| *......*[*Fukomys whytei occlusus*](https://www.ncbi.nlm.nih.gov/Taxonomy/Browser/wwwtax.cgi?id=797420) | [rodents](https://www.ncbi.nlm.nih.gov/Taxonomy/Browser/wwwtax.cgi?id=9989) | 84.2 | [5](https://blast.ncbi.nlm.nih.gov/Blast.cgi) |
| *......*[*Grammomys sp. Berega*](https://www.ncbi.nlm.nih.gov/Taxonomy/Browser/wwwtax.cgi?id=122201) | [rodents](https://www.ncbi.nlm.nih.gov/Taxonomy/Browser/wwwtax.cgi?id=9989) | 84.2 | [1](https://blast.ncbi.nlm.nih.gov/Blast.cgi) |
| *......Pithecia sp.* UFPA Pit22 | [primates](https://www.ncbi.nlm.nih.gov/Taxonomy/Browser/wwwtax.cgi?id=9443) | 82.4 | [1](https://blast.ncbi.nlm.nih.gov/Blast.cgi) |
| *......*[*Xenothrix mcgregori*](https://www.ncbi.nlm.nih.gov/Taxonomy/Browser/wwwtax.cgi?id=2490931) | [primates](https://www.ncbi.nlm.nih.gov/Taxonomy/Browser/wwwtax.cgi?id=9443) | 82.4 | [1](https://blast.ncbi.nlm.nih.gov/Blast.cgi) |
| *......*[*Muscardinus avellanarius*](https://www.ncbi.nlm.nih.gov/Taxonomy/Browser/wwwtax.cgi?id=39082) | [rodents](https://www.ncbi.nlm.nih.gov/Taxonomy/Browser/wwwtax.cgi?id=9989) | 82.4 | [3](https://blast.ncbi.nlm.nih.gov/Blast.cgi) |
| *......*[*Ateles geoffroyi*](https://www.ncbi.nlm.nih.gov/Taxonomy/Browser/wwwtax.cgi?id=9509) | [primates](https://www.ncbi.nlm.nih.gov/Taxonomy/Browser/wwwtax.cgi?id=9443) | 82.4 | [1](https://blast.ncbi.nlm.nih.gov/Blast.cgi) |
| *......*[*Pithecia pithecia*](https://www.ncbi.nlm.nih.gov/Taxonomy/Browser/wwwtax.cgi?id=43777) | [primates](https://www.ncbi.nlm.nih.gov/Taxonomy/Browser/wwwtax.cgi?id=9443) | 82.4 | [3](https://blast.ncbi.nlm.nih.gov/Blast.cgi) |
| *......*[*Pithecia chrysocephala*](https://www.ncbi.nlm.nih.gov/Taxonomy/Browser/wwwtax.cgi?id=2946515) | [primates](https://www.ncbi.nlm.nih.gov/Taxonomy/Browser/wwwtax.cgi?id=9443) | 82.4 | [1](https://blast.ncbi.nlm.nih.gov/Blast.cgi) |
| *......*[*Aotus vociferans*](https://www.ncbi.nlm.nih.gov/Taxonomy/Browser/wwwtax.cgi?id=57176) | [primates](https://www.ncbi.nlm.nih.gov/Taxonomy/Browser/wwwtax.cgi?id=9443) | 82.4 | [2](https://blast.ncbi.nlm.nih.gov/Blast.cgi) |
| *......*[*Aotus nancymaae*](https://www.ncbi.nlm.nih.gov/Taxonomy/Browser/wwwtax.cgi?id=37293) | [primates](https://www.ncbi.nlm.nih.gov/Taxonomy/Browser/wwwtax.cgi?id=9443) | 82.4 | [9](https://blast.ncbi.nlm.nih.gov/Blast.cgi) |
| *......*[*Chiromyscus thomasi*](https://www.ncbi.nlm.nih.gov/Taxonomy/Browser/wwwtax.cgi?id=1572694) | [rodents](https://www.ncbi.nlm.nih.gov/Taxonomy/Browser/wwwtax.cgi?id=9989) | 82.4 | [6](https://blast.ncbi.nlm.nih.gov/Blast.cgi) |
| *......*[*Lepus mandshuricus*](https://www.ncbi.nlm.nih.gov/Taxonomy/Browser/wwwtax.cgi?id=112021) | [rabbits & hares](https://www.ncbi.nlm.nih.gov/Taxonomy/Browser/wwwtax.cgi?id=9975) | 82.4 | [3](https://blast.ncbi.nlm.nih.gov/Blast.cgi) |
| *......*[*Lepus capensis*](https://www.ncbi.nlm.nih.gov/Taxonomy/Browser/wwwtax.cgi?id=9981) | [rabbits & hares](https://www.ncbi.nlm.nih.gov/Taxonomy/Browser/wwwtax.cgi?id=9975) | 82.4 | [1](https://blast.ncbi.nlm.nih.gov/Blast.cgi) |
| *......*[*Golunda ellioti ellioti*](https://www.ncbi.nlm.nih.gov/Taxonomy/Browser/wwwtax.cgi?id=2162857) | [rodents](https://www.ncbi.nlm.nih.gov/Taxonomy/Browser/wwwtax.cgi?id=9989) | 80.5 | [1](https://blast.ncbi.nlm.nih.gov/Blast.cgi) |
| *......*[*Myodes rex*](https://www.ncbi.nlm.nih.gov/Taxonomy/Browser/wwwtax.cgi?id=565117) | [rodents](https://www.ncbi.nlm.nih.gov/Taxonomy/Browser/wwwtax.cgi?id=9989) | 80.5 | [23](https://blast.ncbi.nlm.nih.gov/Blast.cgi) |
| *......*[*Niviventer fulvescens*](https://www.ncbi.nlm.nih.gov/Taxonomy/Browser/wwwtax.cgi?id=83774) | [rodents](https://www.ncbi.nlm.nih.gov/Taxonomy/Browser/wwwtax.cgi?id=9989) | 80.5 | [11](https://blast.ncbi.nlm.nih.gov/Blast.cgi) |
| *......*[*Cheracebus purinus*](https://www.ncbi.nlm.nih.gov/Taxonomy/Browser/wwwtax.cgi?id=1560619) | [primates](https://www.ncbi.nlm.nih.gov/Taxonomy/Browser/wwwtax.cgi?id=9443) | 80.5 | [6](https://blast.ncbi.nlm.nih.gov/Blast.cgi) |
| *......*[*Leopoldamys sabanus*](https://www.ncbi.nlm.nih.gov/Taxonomy/Browser/wwwtax.cgi?id=69073) | [rodents](https://www.ncbi.nlm.nih.gov/Taxonomy/Browser/wwwtax.cgi?id=9989) | 80.5 | [3](https://blast.ncbi.nlm.nih.gov/Blast.cgi) |
| *......*[*Cheracebus regulus*](https://www.ncbi.nlm.nih.gov/Taxonomy/Browser/wwwtax.cgi?id=1812110) | [primates](https://www.ncbi.nlm.nih.gov/Taxonomy/Browser/wwwtax.cgi?id=9443) | 80.5 | [3](https://blast.ncbi.nlm.nih.gov/Blast.cgi) |
| *......*[*Sundasciurus hippurus*](https://www.ncbi.nlm.nih.gov/Taxonomy/Browser/wwwtax.cgi?id=1620830) | [rodents](https://www.ncbi.nlm.nih.gov/Taxonomy/Browser/wwwtax.cgi?id=9989) | 80.5 | [1](https://blast.ncbi.nlm.nih.gov/Blast.cgi) |
| *......*[*Niviventer mekongis*](https://www.ncbi.nlm.nih.gov/Taxonomy/Browser/wwwtax.cgi?id=2773613) | [rodents](https://www.ncbi.nlm.nih.gov/Taxonomy/Browser/wwwtax.cgi?id=9989) | 80.5 | [9](https://blast.ncbi.nlm.nih.gov/Blast.cgi) |
| *......*[*Leopoldamys neilli*](https://www.ncbi.nlm.nih.gov/Taxonomy/Browser/wwwtax.cgi?id=857467) | [rodents](https://www.ncbi.nlm.nih.gov/Taxonomy/Browser/wwwtax.cgi?id=9989) | 80.5 | [3](https://blast.ncbi.nlm.nih.gov/Blast.cgi) |
| *......*[*Microtus rossiaemeridionalis*](https://www.ncbi.nlm.nih.gov/Taxonomy/Browser/wwwtax.cgi?id=537919) | [rodents](https://www.ncbi.nlm.nih.gov/Taxonomy/Browser/wwwtax.cgi?id=9989) | 80.5 | [4](https://blast.ncbi.nlm.nih.gov/Blast.cgi) |
| *......*[*Microtus miurus*](https://www.ncbi.nlm.nih.gov/Taxonomy/Browser/wwwtax.cgi?id=111837) | [rodents](https://www.ncbi.nlm.nih.gov/Taxonomy/Browser/wwwtax.cgi?id=9989) | 80.5 | [2](https://blast.ncbi.nlm.nih.gov/Blast.cgi) |
| *......*[*Microtus ochrogaster*](https://www.ncbi.nlm.nih.gov/Taxonomy/Browser/wwwtax.cgi?id=79684) | [rodents](https://www.ncbi.nlm.nih.gov/Taxonomy/Browser/wwwtax.cgi?id=9989) | 80.5 | [1](https://blast.ncbi.nlm.nih.gov/Blast.cgi) |
| *......*[*Microtus californicus*](https://www.ncbi.nlm.nih.gov/Taxonomy/Browser/wwwtax.cgi?id=100895) | [rodents](https://www.ncbi.nlm.nih.gov/Taxonomy/Browser/wwwtax.cgi?id=9989) | 80.5 | [2](https://blast.ncbi.nlm.nih.gov/Blast.cgi) |
| *......*[*Niviventer niviventer*](https://www.ncbi.nlm.nih.gov/Taxonomy/Browser/wwwtax.cgi?id=83757) | [rodents](https://www.ncbi.nlm.nih.gov/Taxonomy/Browser/wwwtax.cgi?id=9989) | 80.5 | [1](https://blast.ncbi.nlm.nih.gov/Blast.cgi) |
| *......*[*Niviventer andersoni*](https://www.ncbi.nlm.nih.gov/Taxonomy/Browser/wwwtax.cgi?id=425032) | [rodents](https://www.ncbi.nlm.nih.gov/Taxonomy/Browser/wwwtax.cgi?id=9989) | 80.5 | [1](https://blast.ncbi.nlm.nih.gov/Blast.cgi) |
| *......*[*Microtus townsendii*](https://www.ncbi.nlm.nih.gov/Taxonomy/Browser/wwwtax.cgi?id=111841) | [rodents](https://www.ncbi.nlm.nih.gov/Taxonomy/Browser/wwwtax.cgi?id=9989) | 80.5 | [1](https://blast.ncbi.nlm.nih.gov/Blast.cgi) |
| *......*[*Microtus pennsylvanicus*](https://www.ncbi.nlm.nih.gov/Taxonomy/Browser/wwwtax.cgi?id=10058) | [rodents](https://www.ncbi.nlm.nih.gov/Taxonomy/Browser/wwwtax.cgi?id=9989) | 80.5 | [1](https://blast.ncbi.nlm.nih.gov/Blast.cgi) |
| *......*[*Alexandromys oeconomus*](https://www.ncbi.nlm.nih.gov/Taxonomy/Browser/wwwtax.cgi?id=2162900) | [rodents](https://www.ncbi.nlm.nih.gov/Taxonomy/Browser/wwwtax.cgi?id=9989) | 80.5 | [2](https://blast.ncbi.nlm.nih.gov/Blast.cgi) |
| *......*[*Microtus montanus*](https://www.ncbi.nlm.nih.gov/Taxonomy/Browser/wwwtax.cgi?id=88450) | [rodents](https://www.ncbi.nlm.nih.gov/Taxonomy/Browser/wwwtax.cgi?id=9989) | 80.5 | [1](https://blast.ncbi.nlm.nih.gov/Blast.cgi) |
| *......*[*Apodemus peninsulae*](https://www.ncbi.nlm.nih.gov/Taxonomy/Browser/wwwtax.cgi?id=105297) | [rodents](https://www.ncbi.nlm.nih.gov/Taxonomy/Browser/wwwtax.cgi?id=9989) | 78.7 | [31](https://blast.ncbi.nlm.nih.gov/Blast.cgi) |
| *......*[*Apodemus latronum*](https://www.ncbi.nlm.nih.gov/Taxonomy/Browser/wwwtax.cgi?id=214933) | [rodents](https://www.ncbi.nlm.nih.gov/Taxonomy/Browser/wwwtax.cgi?id=9989) | 78.7 | [3](https://blast.ncbi.nlm.nih.gov/Blast.cgi) |
| *......Jaculus sp.* 'ex gr jaculus' | [rodents](https://www.ncbi.nlm.nih.gov/Taxonomy/Browser/wwwtax.cgi?id=9989) | 78.7 | [1](https://blast.ncbi.nlm.nih.gov/Blast.cgi) |
| *......*[*Graphiurus kelleni*](https://www.ncbi.nlm.nih.gov/Taxonomy/Browser/wwwtax.cgi?id=1223622) | [rodents](https://www.ncbi.nlm.nih.gov/Taxonomy/Browser/wwwtax.cgi?id=9989) | 78.7 | [1](https://blast.ncbi.nlm.nih.gov/Blast.cgi) |
| *......*[*Jaculus orientalis*](https://www.ncbi.nlm.nih.gov/Taxonomy/Browser/wwwtax.cgi?id=48868) | [rodents](https://www.ncbi.nlm.nih.gov/Taxonomy/Browser/wwwtax.cgi?id=9989) | 78.7 | [3](https://blast.ncbi.nlm.nih.gov/Blast.cgi) |
| *......Heliophobius sp.* CGF-2010a | [rodents](https://www.ncbi.nlm.nih.gov/Taxonomy/Browser/wwwtax.cgi?id=9989) | 78.7 | [2](https://blast.ncbi.nlm.nih.gov/Blast.cgi) |
| *......*[*Clethrionomys gapperi*](https://www.ncbi.nlm.nih.gov/Taxonomy/Browser/wwwtax.cgi?id=473866) | [rodents](https://www.ncbi.nlm.nih.gov/Taxonomy/Browser/wwwtax.cgi?id=9989) | 78.7 | [4](https://blast.ncbi.nlm.nih.gov/Blast.cgi) |
| *......*[*Reithrodontomys mexicanus*](https://www.ncbi.nlm.nih.gov/Taxonomy/Browser/wwwtax.cgi?id=89150) | [rodents](https://www.ncbi.nlm.nih.gov/Taxonomy/Browser/wwwtax.cgi?id=9989) | 78.7 | [2](https://blast.ncbi.nlm.nih.gov/Blast.cgi) |
| *......*[*Fukomys amatus*](https://www.ncbi.nlm.nih.gov/Taxonomy/Browser/wwwtax.cgi?id=423608) | [rodents](https://www.ncbi.nlm.nih.gov/Taxonomy/Browser/wwwtax.cgi?id=9989) | 78.7 | [4](https://blast.ncbi.nlm.nih.gov/Blast.cgi) |
| *......*[*Dipodomys phillipsii*](https://www.ncbi.nlm.nih.gov/Taxonomy/Browser/wwwtax.cgi?id=108146) | [rodents](https://www.ncbi.nlm.nih.gov/Taxonomy/Browser/wwwtax.cgi?id=9989) | 76.8 | [10](https://blast.ncbi.nlm.nih.gov/Blast.cgi) |
| *......*[*Chiropotes chiropotes*](https://www.ncbi.nlm.nih.gov/Taxonomy/Browser/wwwtax.cgi?id=658221) | [primates](https://www.ncbi.nlm.nih.gov/Taxonomy/Browser/wwwtax.cgi?id=9443) | 76.8 | [1](https://blast.ncbi.nlm.nih.gov/Blast.cgi) |
| *......*[*Chiromyscus chiropus*](https://www.ncbi.nlm.nih.gov/Taxonomy/Browser/wwwtax.cgi?id=491872) | [rodents](https://www.ncbi.nlm.nih.gov/Taxonomy/Browser/wwwtax.cgi?id=9989) | 76.8 | [2](https://blast.ncbi.nlm.nih.gov/Blast.cgi) |
| *......*[*Chiropotes utahickae*](https://www.ncbi.nlm.nih.gov/Taxonomy/Browser/wwwtax.cgi?id=280160) | [primates](https://www.ncbi.nlm.nih.gov/Taxonomy/Browser/wwwtax.cgi?id=9443) | 76.8 | [1](https://blast.ncbi.nlm.nih.gov/Blast.cgi) |
| *......*[*Sapajus xanthosternos*](https://www.ncbi.nlm.nih.gov/Taxonomy/Browser/wwwtax.cgi?id=174599) | [primates](https://www.ncbi.nlm.nih.gov/Taxonomy/Browser/wwwtax.cgi?id=9443) | 75.0 | [8](https://blast.ncbi.nlm.nih.gov/Blast.cgi) |
| *......*[*Callicebus nigrifrons*](https://www.ncbi.nlm.nih.gov/Taxonomy/Browser/wwwtax.cgi?id=867334) | [primates](https://www.ncbi.nlm.nih.gov/Taxonomy/Browser/wwwtax.cgi?id=9443) | 75.0 | [2](https://blast.ncbi.nlm.nih.gov/Blast.cgi) |
| *......*[*Microtus xanthognathus*](https://www.ncbi.nlm.nih.gov/Taxonomy/Browser/wwwtax.cgi?id=10054) | [rodents](https://www.ncbi.nlm.nih.gov/Taxonomy/Browser/wwwtax.cgi?id=9989) | 75.0 | [1](https://blast.ncbi.nlm.nih.gov/Blast.cgi) |
| *......*[*Microtus chrotorrhinus*](https://www.ncbi.nlm.nih.gov/Taxonomy/Browser/wwwtax.cgi?id=10055) | [rodents](https://www.ncbi.nlm.nih.gov/Taxonomy/Browser/wwwtax.cgi?id=9989) | 75.0 | [1](https://blast.ncbi.nlm.nih.gov/Blast.cgi) |
| *......*[*Saccostomus umbriventer*](https://www.ncbi.nlm.nih.gov/Taxonomy/Browser/wwwtax.cgi?id=1582485) | [rodents](https://www.ncbi.nlm.nih.gov/Taxonomy/Browser/wwwtax.cgi?id=9989) | 73.1 | [2](https://blast.ncbi.nlm.nih.gov/Blast.cgi) |
| *......*[*Aotus lemurinus*](https://www.ncbi.nlm.nih.gov/Taxonomy/Browser/wwwtax.cgi?id=43147) | [primates](https://www.ncbi.nlm.nih.gov/Taxonomy/Browser/wwwtax.cgi?id=9443) | 73.1 | [2](https://blast.ncbi.nlm.nih.gov/Blast.cgi) |
| *......*[*Mus booduga*](https://www.ncbi.nlm.nih.gov/Taxonomy/Browser/wwwtax.cgi?id=27681) | [rodents](https://www.ncbi.nlm.nih.gov/Taxonomy/Browser/wwwtax.cgi?id=9989) | 73.1 | [1](https://blast.ncbi.nlm.nih.gov/Blast.cgi) |
| *......*[*Sundasciurus tenuis*](https://www.ncbi.nlm.nih.gov/Taxonomy/Browser/wwwtax.cgi?id=1620839) | [rodents](https://www.ncbi.nlm.nih.gov/Taxonomy/Browser/wwwtax.cgi?id=9989) | 71.3 | [1](https://blast.ncbi.nlm.nih.gov/Blast.cgi) |
| *......*[*Cebus olivaceus castaneus*](https://www.ncbi.nlm.nih.gov/Taxonomy/Browser/wwwtax.cgi?id=1985287) | [primates](https://www.ncbi.nlm.nih.gov/Taxonomy/Browser/wwwtax.cgi?id=9443) | 71.3 | [3](https://blast.ncbi.nlm.nih.gov/Blast.cgi) |
| *......*[*Microtus kikuchii*](https://www.ncbi.nlm.nih.gov/Taxonomy/Browser/wwwtax.cgi?id=100899) | [rodents](https://www.ncbi.nlm.nih.gov/Taxonomy/Browser/wwwtax.cgi?id=9989) | 71.3 | [1](https://blast.ncbi.nlm.nih.gov/Blast.cgi) |
| *......*[*Microtus agrestis*](https://www.ncbi.nlm.nih.gov/Taxonomy/Browser/wwwtax.cgi?id=29092) | [rodents](https://www.ncbi.nlm.nih.gov/Taxonomy/Browser/wwwtax.cgi?id=9989) | 71.3 | [1](https://blast.ncbi.nlm.nih.gov/Blast.cgi) |
| *......*[*Microtus abbreviatus*](https://www.ncbi.nlm.nih.gov/Taxonomy/Browser/wwwtax.cgi?id=100894) | [rodents](https://www.ncbi.nlm.nih.gov/Taxonomy/Browser/wwwtax.cgi?id=9989) | 71.3 | [1](https://blast.ncbi.nlm.nih.gov/Blast.cgi) |
| *......*[*Microtus arvalis*](https://www.ncbi.nlm.nih.gov/Taxonomy/Browser/wwwtax.cgi?id=47230) | [rodents](https://www.ncbi.nlm.nih.gov/Taxonomy/Browser/wwwtax.cgi?id=9989) | 71.3 | [1](https://blast.ncbi.nlm.nih.gov/Blast.cgi) |
| *......*[*Mico humilis*](https://www.ncbi.nlm.nih.gov/Taxonomy/Browser/wwwtax.cgi?id=666519) | [primates](https://www.ncbi.nlm.nih.gov/Taxonomy/Browser/wwwtax.cgi?id=9443) | 69.4 | [3](https://blast.ncbi.nlm.nih.gov/Blast.cgi) |
| *......*[*Cebus kaapori*](https://www.ncbi.nlm.nih.gov/Taxonomy/Browser/wwwtax.cgi?id=37294) | [primates](https://www.ncbi.nlm.nih.gov/Taxonomy/Browser/wwwtax.cgi?id=9443) | 67.6 | [1](https://blast.ncbi.nlm.nih.gov/Blast.cgi) |
| *......*[*Cebus olivaceus*](https://www.ncbi.nlm.nih.gov/Taxonomy/Browser/wwwtax.cgi?id=37295) | [primates](https://www.ncbi.nlm.nih.gov/Taxonomy/Browser/wwwtax.cgi?id=9443) | 67.6 | [6](https://blast.ncbi.nlm.nih.gov/Blast.cgi) |
| *......*[*Sapajus cay*](https://www.ncbi.nlm.nih.gov/Taxonomy/Browser/wwwtax.cgi?id=649471) | [primates](https://www.ncbi.nlm.nih.gov/Taxonomy/Browser/wwwtax.cgi?id=9443) | 67.6 | [1](https://blast.ncbi.nlm.nih.gov/Blast.cgi) |
| *......*[*Apodemus agrarius*](https://www.ncbi.nlm.nih.gov/Taxonomy/Browser/wwwtax.cgi?id=39030) | [rodents](https://www.ncbi.nlm.nih.gov/Taxonomy/Browser/wwwtax.cgi?id=9989) | 67.6 | [1](https://blast.ncbi.nlm.nih.gov/Blast.cgi) |
| *......*[*Cebus albifrons*](https://www.ncbi.nlm.nih.gov/Taxonomy/Browser/wwwtax.cgi?id=9514) | [primates](https://www.ncbi.nlm.nih.gov/Taxonomy/Browser/wwwtax.cgi?id=9443) | 67.6 | [4](https://blast.ncbi.nlm.nih.gov/Blast.cgi) |
| *......*[*Sigmodon ochrognathus*](https://www.ncbi.nlm.nih.gov/Taxonomy/Browser/wwwtax.cgi?id=56214) | [rodents](https://www.ncbi.nlm.nih.gov/Taxonomy/Browser/wwwtax.cgi?id=9989) | 67.6 | [6](https://blast.ncbi.nlm.nih.gov/Blast.cgi) |
| *......Saccostomus sp.* 50137 | [rodents](https://www.ncbi.nlm.nih.gov/Taxonomy/Browser/wwwtax.cgi?id=9989) | 67.6 | [1](https://blast.ncbi.nlm.nih.gov/Blast.cgi) |
| *......Saccostomus sp.* 50064 | [rodents](https://www.ncbi.nlm.nih.gov/Taxonomy/Browser/wwwtax.cgi?id=9989) | 67.6 | [1](https://blast.ncbi.nlm.nih.gov/Blast.cgi) |
| *......*[*Cebus unicolor*](https://www.ncbi.nlm.nih.gov/Taxonomy/Browser/wwwtax.cgi?id=1985288) | [primates](https://www.ncbi.nlm.nih.gov/Taxonomy/Browser/wwwtax.cgi?id=9443) | 65.8 | [4](https://blast.ncbi.nlm.nih.gov/Blast.cgi) |
| *......*[*Sundasciurus philippinensis*](https://www.ncbi.nlm.nih.gov/Taxonomy/Browser/wwwtax.cgi?id=234689) | [rodents](https://www.ncbi.nlm.nih.gov/Taxonomy/Browser/wwwtax.cgi?id=9989) | 65.8 | [1](https://blast.ncbi.nlm.nih.gov/Blast.cgi) |
| *......*[*Microtus mexicanus*](https://www.ncbi.nlm.nih.gov/Taxonomy/Browser/wwwtax.cgi?id=79689) | [rodents](https://www.ncbi.nlm.nih.gov/Taxonomy/Browser/wwwtax.cgi?id=9989) | 65.8 | [1](https://blast.ncbi.nlm.nih.gov/Blast.cgi) |
| *......*[*Sapajus apella*](https://www.ncbi.nlm.nih.gov/Taxonomy/Browser/wwwtax.cgi?id=9515) | [primates](https://www.ncbi.nlm.nih.gov/Taxonomy/Browser/wwwtax.cgi?id=9443) | 58.4 | [1](https://blast.ncbi.nlm.nih.gov/Blast.cgi) |
| *......*[*Cebus versicolor*](https://www.ncbi.nlm.nih.gov/Taxonomy/Browser/wwwtax.cgi?id=1985289) | [primates](https://www.ncbi.nlm.nih.gov/Taxonomy/Browser/wwwtax.cgi?id=9443) | 54.7 | [1](https://blast.ncbi.nlm.nih.gov/Blast.cgi) |
| *......*[*Dipodomys spectabilis*](https://www.ncbi.nlm.nih.gov/Taxonomy/Browser/wwwtax.cgi?id=105255) | [rodents](https://www.ncbi.nlm.nih.gov/Taxonomy/Browser/wwwtax.cgi?id=9989) | 52.8 | [1](https://blast.ncbi.nlm.nih.gov/Blast.cgi) |
| *.....*[*Chrysospalax trevelyani*](https://www.ncbi.nlm.nih.gov/Taxonomy/Browser/wwwtax.cgi?id=176108) | [placentals](https://www.ncbi.nlm.nih.gov/Taxonomy/Browser/wwwtax.cgi?id=9347) | 117 | [1](https://blast.ncbi.nlm.nih.gov/Blast.cgi) |
| *.....*[*Chrysochloris asiatica*](https://www.ncbi.nlm.nih.gov/Taxonomy/Browser/wwwtax.cgi?id=185453) | [placentals](https://www.ncbi.nlm.nih.gov/Taxonomy/Browser/wwwtax.cgi?id=9347) | 100 | [4](https://blast.ncbi.nlm.nih.gov/Blast.cgi) |
| *.....*[*Neamblysomus gunningi*](https://www.ncbi.nlm.nih.gov/Taxonomy/Browser/wwwtax.cgi?id=745254) | [placentals](https://www.ncbi.nlm.nih.gov/Taxonomy/Browser/wwwtax.cgi?id=9347) | 99.0 | [1](https://blast.ncbi.nlm.nih.gov/Blast.cgi) |
| *.....*[*Amblysomus hottentotus longiceps*](https://www.ncbi.nlm.nih.gov/Taxonomy/Browser/wwwtax.cgi?id=1632278) | [placentals](https://www.ncbi.nlm.nih.gov/Taxonomy/Browser/wwwtax.cgi?id=9347) | 93.5 | [2](https://blast.ncbi.nlm.nih.gov/Blast.cgi) |
| *.....*[*Galegeeska rufescens*](https://www.ncbi.nlm.nih.gov/Taxonomy/Browser/wwwtax.cgi?id=42151) | [placentals](https://www.ncbi.nlm.nih.gov/Taxonomy/Browser/wwwtax.cgi?id=9347) | 91.6 | [1](https://blast.ncbi.nlm.nih.gov/Blast.cgi) |
| *.....*[*Amblysomus septentrionalis*](https://www.ncbi.nlm.nih.gov/Taxonomy/Browser/wwwtax.cgi?id=745241) | [placentals](https://www.ncbi.nlm.nih.gov/Taxonomy/Browser/wwwtax.cgi?id=9347) | 87.9 | [1](https://blast.ncbi.nlm.nih.gov/Blast.cgi) |
| *....*[*Tlacuatzin canescens*](https://www.ncbi.nlm.nih.gov/Taxonomy/Browser/wwwtax.cgi?id=225728) | [marsupials](https://www.ncbi.nlm.nih.gov/Taxonomy/Browser/wwwtax.cgi?id=9263) | 104 | [23](https://blast.ncbi.nlm.nih.gov/Blast.cgi) |
| *....*[*Antechinus swainsonii*](https://www.ncbi.nlm.nih.gov/Taxonomy/Browser/wwwtax.cgi?id=9284) | [marsupials](https://www.ncbi.nlm.nih.gov/Taxonomy/Browser/wwwtax.cgi?id=9263) | 93.5 | [1](https://blast.ncbi.nlm.nih.gov/Blast.cgi) |
| *....*[*Phascolosorex dorsalis*](https://www.ncbi.nlm.nih.gov/Taxonomy/Browser/wwwtax.cgi?id=9295) | [marsupials](https://www.ncbi.nlm.nih.gov/Taxonomy/Browser/wwwtax.cgi?id=9263) | 91.6 | [2](https://blast.ncbi.nlm.nih.gov/Blast.cgi) |
| *....*[*Murexia rothschildi*](https://www.ncbi.nlm.nih.gov/Taxonomy/Browser/wwwtax.cgi?id=418657) | [marsupials](https://www.ncbi.nlm.nih.gov/Taxonomy/Browser/wwwtax.cgi?id=9263) | 89.8 | [1](https://blast.ncbi.nlm.nih.gov/Blast.cgi) |
| *...*[*Podarcis hispanicus*](https://www.ncbi.nlm.nih.gov/Taxonomy/Browser/wwwtax.cgi?id=74081) | [lizards & snakes](https://www.ncbi.nlm.nih.gov/Taxonomy/Browser/wwwtax.cgi?id=8504) | 99.0 | [12](https://blast.ncbi.nlm.nih.gov/Blast.cgi) |
| *...*[*Podarcis filfolensis*](https://www.ncbi.nlm.nih.gov/Taxonomy/Browser/wwwtax.cgi?id=65481) | [lizards & snakes](https://www.ncbi.nlm.nih.gov/Taxonomy/Browser/wwwtax.cgi?id=8504) | 93.5 | [3](https://blast.ncbi.nlm.nih.gov/Blast.cgi) |
| *...*[*Teira dugesii*](https://www.ncbi.nlm.nih.gov/Taxonomy/Browser/wwwtax.cgi?id=106894) | [lizards & snakes](https://www.ncbi.nlm.nih.gov/Taxonomy/Browser/wwwtax.cgi?id=8504) | 89.8 | [2](https://blast.ncbi.nlm.nih.gov/Blast.cgi) |
| *...*[*Lacerta viridis viridis*](https://www.ncbi.nlm.nih.gov/Taxonomy/Browser/wwwtax.cgi?id=340657) | [lizards & snakes](https://www.ncbi.nlm.nih.gov/Taxonomy/Browser/wwwtax.cgi?id=8504) | 87.9 | [322](https://blast.ncbi.nlm.nih.gov/Blast.cgi) |
| *...*[*Phrynosoma douglasii*](https://www.ncbi.nlm.nih.gov/Taxonomy/Browser/wwwtax.cgi?id=43611) | [lizards & snakes](https://www.ncbi.nlm.nih.gov/Taxonomy/Browser/wwwtax.cgi?id=8504) | 87.9 | [11](https://blast.ncbi.nlm.nih.gov/Blast.cgi) |
| *...*[*Lacerta bilineata*](https://www.ncbi.nlm.nih.gov/Taxonomy/Browser/wwwtax.cgi?id=95620) | [lizards & snakes](https://www.ncbi.nlm.nih.gov/Taxonomy/Browser/wwwtax.cgi?id=8504) | 82.4 | [135](https://blast.ncbi.nlm.nih.gov/Blast.cgi) |
| *...*[*Sceloporus horridus*](https://www.ncbi.nlm.nih.gov/Taxonomy/Browser/wwwtax.cgi?id=59700) | [lizards & snakes](https://www.ncbi.nlm.nih.gov/Taxonomy/Browser/wwwtax.cgi?id=8504) | 82.4 | [2](https://blast.ncbi.nlm.nih.gov/Blast.cgi) |
| *...*[*Lacerta viridis*](https://www.ncbi.nlm.nih.gov/Taxonomy/Browser/wwwtax.cgi?id=65476) | [lizards & snakes](https://www.ncbi.nlm.nih.gov/Taxonomy/Browser/wwwtax.cgi?id=8504) | 82.4 | [31](https://blast.ncbi.nlm.nih.gov/Blast.cgi) |
| *...*[*Urosaurus ornatus*](https://www.ncbi.nlm.nih.gov/Taxonomy/Browser/wwwtax.cgi?id=43650) | [lizards & snakes](https://www.ncbi.nlm.nih.gov/Taxonomy/Browser/wwwtax.cgi?id=8504) | 82.4 | [2](https://blast.ncbi.nlm.nih.gov/Blast.cgi) |
| *...*[*Lacerta bilineata chlorosecunda*](https://www.ncbi.nlm.nih.gov/Taxonomy/Browser/wwwtax.cgi?id=1604043) | [lizards & snakes](https://www.ncbi.nlm.nih.gov/Taxonomy/Browser/wwwtax.cgi?id=8504) | 82.4 | [1](https://blast.ncbi.nlm.nih.gov/Blast.cgi) |
| *...*[*Lacerta viridis meridionalis*](https://www.ncbi.nlm.nih.gov/Taxonomy/Browser/wwwtax.cgi?id=340666) | [lizards & snakes](https://www.ncbi.nlm.nih.gov/Taxonomy/Browser/wwwtax.cgi?id=8504) | 82.4 | [5](https://blast.ncbi.nlm.nih.gov/Blast.cgi) |
| *...*[*Zonosaurus madagascariensis*](https://www.ncbi.nlm.nih.gov/Taxonomy/Browser/wwwtax.cgi?id=143662) | [lizards & snakes](https://www.ncbi.nlm.nih.gov/Taxonomy/Browser/wwwtax.cgi?id=8504) | 80.5 | [14](https://blast.ncbi.nlm.nih.gov/Blast.cgi) |
| *...*[*Zonosaurus laticaudatus*](https://www.ncbi.nlm.nih.gov/Taxonomy/Browser/wwwtax.cgi?id=219626) | [lizards & snakes](https://www.ncbi.nlm.nih.gov/Taxonomy/Browser/wwwtax.cgi?id=8504) | 80.5 | [2](https://blast.ncbi.nlm.nih.gov/Blast.cgi) |
| *...*[*Lacerta trilineata*](https://www.ncbi.nlm.nih.gov/Taxonomy/Browser/wwwtax.cgi?id=92836) | [lizards & snakes](https://www.ncbi.nlm.nih.gov/Taxonomy/Browser/wwwtax.cgi?id=8504) | 78.7 | [14](https://blast.ncbi.nlm.nih.gov/Blast.cgi) |
| *...*[*Phrynosoma taurus*](https://www.ncbi.nlm.nih.gov/Taxonomy/Browser/wwwtax.cgi?id=159096) | [lizards & snakes](https://www.ncbi.nlm.nih.gov/Taxonomy/Browser/wwwtax.cgi?id=8504) | 78.7 | [1](https://blast.ncbi.nlm.nih.gov/Blast.cgi) |
| *...*[*Lacerta bilineata bilineata*](https://www.ncbi.nlm.nih.gov/Taxonomy/Browser/wwwtax.cgi?id=340664) | [lizards & snakes](https://www.ncbi.nlm.nih.gov/Taxonomy/Browser/wwwtax.cgi?id=8504) | 76.8 | [7](https://blast.ncbi.nlm.nih.gov/Blast.cgi) |
| *...*[*Lacerta pamphylica*](https://www.ncbi.nlm.nih.gov/Taxonomy/Browser/wwwtax.cgi?id=95621) | [lizards & snakes](https://www.ncbi.nlm.nih.gov/Taxonomy/Browser/wwwtax.cgi?id=8504) | 76.8 | [3](https://blast.ncbi.nlm.nih.gov/Blast.cgi) |
| *...*[*Lacerta bilineata chloronota*](https://www.ncbi.nlm.nih.gov/Taxonomy/Browser/wwwtax.cgi?id=119061) | [lizards & snakes](https://www.ncbi.nlm.nih.gov/Taxonomy/Browser/wwwtax.cgi?id=8504) | 76.8 | [1](https://blast.ncbi.nlm.nih.gov/Blast.cgi) |
| *...*[*Lacerta bilineata fejervaryi*](https://www.ncbi.nlm.nih.gov/Taxonomy/Browser/wwwtax.cgi?id=1604044) | [lizards & snakes](https://www.ncbi.nlm.nih.gov/Taxonomy/Browser/wwwtax.cgi?id=8504) | 76.8 | [1](https://blast.ncbi.nlm.nih.gov/Blast.cgi) |
| *...*[*Zonosaurus haraldmeieri*](https://www.ncbi.nlm.nih.gov/Taxonomy/Browser/wwwtax.cgi?id=329868) | [lizards & snakes](https://www.ncbi.nlm.nih.gov/Taxonomy/Browser/wwwtax.cgi?id=8504) | 75.0 | [2](https://blast.ncbi.nlm.nih.gov/Blast.cgi) |
| *...*[*Zonosaurus trilineatus*](https://www.ncbi.nlm.nih.gov/Taxonomy/Browser/wwwtax.cgi?id=174286) | [lizards & snakes](https://www.ncbi.nlm.nih.gov/Taxonomy/Browser/wwwtax.cgi?id=8504) | 75.0 | [1](https://blast.ncbi.nlm.nih.gov/Blast.cgi) |
| *..*[*Hypseleotris barrawayi*](https://www.ncbi.nlm.nih.gov/Taxonomy/Browser/wwwtax.cgi?id=2849998) | [bony fishes](https://www.ncbi.nlm.nih.gov/Taxonomy/Browser/wwwtax.cgi?id=7898) | 93.5 | [1](https://blast.ncbi.nlm.nih.gov/Blast.cgi) |
| *..*[*Odontobutis potamophila*](https://www.ncbi.nlm.nih.gov/Taxonomy/Browser/wwwtax.cgi?id=308085) | [bony fishes](https://www.ncbi.nlm.nih.gov/Taxonomy/Browser/wwwtax.cgi?id=7898) | 87.9 | [236](https://blast.ncbi.nlm.nih.gov/Blast.cgi) |
| *..*[*Gymnogobius breunigii*](https://www.ncbi.nlm.nih.gov/Taxonomy/Browser/wwwtax.cgi?id=262095) | [bony fishes](https://www.ncbi.nlm.nih.gov/Taxonomy/Browser/wwwtax.cgi?id=7898) | 84.2 | [7](https://blast.ncbi.nlm.nih.gov/Blast.cgi) |
| *..*[*Redigobius sp. RediF*](https://www.ncbi.nlm.nih.gov/Taxonomy/Browser/wwwtax.cgi?id=1410379) | [bony fishes](https://www.ncbi.nlm.nih.gov/Taxonomy/Browser/wwwtax.cgi?id=7898) | 82.4 | [1](https://blast.ncbi.nlm.nih.gov/Blast.cgi) |
| *..*[*Gobiosoma hildebrandi*](https://www.ncbi.nlm.nih.gov/Taxonomy/Browser/wwwtax.cgi?id=203307) | [bony fishes](https://www.ncbi.nlm.nih.gov/Taxonomy/Browser/wwwtax.cgi?id=7898) | 78.7 | [1](https://blast.ncbi.nlm.nih.gov/Blast.cgi) |
| *..*[*Acipenser gueldenstaedtii*](https://www.ncbi.nlm.nih.gov/Taxonomy/Browser/wwwtax.cgi?id=7902) | [bony fishes](https://www.ncbi.nlm.nih.gov/Taxonomy/Browser/wwwtax.cgi?id=7898) | 76.8 | [44](https://blast.ncbi.nlm.nih.gov/Blast.cgi) |
| *..*[*Acipenser persicus*](https://www.ncbi.nlm.nih.gov/Taxonomy/Browser/wwwtax.cgi?id=61968) | [bony fishes](https://www.ncbi.nlm.nih.gov/Taxonomy/Browser/wwwtax.cgi?id=7898) | 76.8 | [6](https://blast.ncbi.nlm.nih.gov/Blast.cgi) |
| *..*[*Schismatogobius ninja*](https://www.ncbi.nlm.nih.gov/Taxonomy/Browser/wwwtax.cgi?id=1986417) | [bony fishes](https://www.ncbi.nlm.nih.gov/Taxonomy/Browser/wwwtax.cgi?id=7898) | 73.1 | [16](https://blast.ncbi.nlm.nih.gov/Blast.cgi) |
| *..*[*Acipenser gueldenstaedtii x Acipenser baerii*](https://www.ncbi.nlm.nih.gov/Taxonomy/Browser/wwwtax.cgi?id=1358432) | [bony fishes](https://www.ncbi.nlm.nih.gov/Taxonomy/Browser/wwwtax.cgi?id=7898) | 71.3 | [2](https://blast.ncbi.nlm.nih.gov/Blast.cgi) |
| *..*[*Hypseleotris klunzingeri*](https://www.ncbi.nlm.nih.gov/Taxonomy/Browser/wwwtax.cgi?id=166778) | [bony fishes](https://www.ncbi.nlm.nih.gov/Taxonomy/Browser/wwwtax.cgi?id=7898) | 71.3 | [10](https://blast.ncbi.nlm.nih.gov/Blast.cgi) |
| *..*[*Acipenser baerii*](https://www.ncbi.nlm.nih.gov/Taxonomy/Browser/wwwtax.cgi?id=27689) | [bony fishes](https://www.ncbi.nlm.nih.gov/Taxonomy/Browser/wwwtax.cgi?id=7898) | 71.3 | [7](https://blast.ncbi.nlm.nih.gov/Blast.cgi) |
| *..*[*Acipenser schrenckii x Acipenser baerii*](https://www.ncbi.nlm.nih.gov/Taxonomy/Browser/wwwtax.cgi?id=1150732) | [bony fishes](https://www.ncbi.nlm.nih.gov/Taxonomy/Browser/wwwtax.cgi?id=7898) | 71.3 | [2](https://blast.ncbi.nlm.nih.gov/Blast.cgi) |
| *..*[*Astronesthes lucifer*](https://www.ncbi.nlm.nih.gov/Taxonomy/Browser/wwwtax.cgi?id=1263173) | [bony fishes](https://www.ncbi.nlm.nih.gov/Taxonomy/Browser/wwwtax.cgi?id=7898) | 71.3 | [1](https://blast.ncbi.nlm.nih.gov/Blast.cgi) |
| *..*[*Balistes vetula*](https://www.ncbi.nlm.nih.gov/Taxonomy/Browser/wwwtax.cgi?id=303687) | [bony fishes](https://www.ncbi.nlm.nih.gov/Taxonomy/Browser/wwwtax.cgi?id=7898) | 71.3 | [4](https://blast.ncbi.nlm.nih.gov/Blast.cgi) |
| *..*[*Acipenser naccarii*](https://www.ncbi.nlm.nih.gov/Taxonomy/Browser/wwwtax.cgi?id=42330) | [bony fishes](https://www.ncbi.nlm.nih.gov/Taxonomy/Browser/wwwtax.cgi?id=7898) | 71.3 | [3](https://blast.ncbi.nlm.nih.gov/Blast.cgi) |
| *..*[*Scartelaos histophorus*](https://www.ncbi.nlm.nih.gov/Taxonomy/Browser/wwwtax.cgi?id=166764) | [bony fishes](https://www.ncbi.nlm.nih.gov/Taxonomy/Browser/wwwtax.cgi?id=7898) | 71.3 | [1](https://blast.ncbi.nlm.nih.gov/Blast.cgi) |
| *..*[*Gobiopsis arenaria*](https://www.ncbi.nlm.nih.gov/Taxonomy/Browser/wwwtax.cgi?id=1365554) | [bony fishes](https://www.ncbi.nlm.nih.gov/Taxonomy/Browser/wwwtax.cgi?id=7898) | 67.6 | [1](https://blast.ncbi.nlm.nih.gov/Blast.cgi) |
| *..*[*Huso huso*](https://www.ncbi.nlm.nih.gov/Taxonomy/Browser/wwwtax.cgi?id=61971) | [bony fishes](https://www.ncbi.nlm.nih.gov/Taxonomy/Browser/wwwtax.cgi?id=7898) | 65.8 | [12](https://blast.ncbi.nlm.nih.gov/Blast.cgi) |
| *..*[*Acipenser brevirostrum*](https://www.ncbi.nlm.nih.gov/Taxonomy/Browser/wwwtax.cgi?id=7907) | [bony fishes](https://www.ncbi.nlm.nih.gov/Taxonomy/Browser/wwwtax.cgi?id=7898) | 62.1 | [13](https://blast.ncbi.nlm.nih.gov/Blast.cgi) |
| *..*[*Gnatholepis thompsoni*](https://www.ncbi.nlm.nih.gov/Taxonomy/Browser/wwwtax.cgi?id=151728) | [bony fishes](https://www.ncbi.nlm.nih.gov/Taxonomy/Browser/wwwtax.cgi?id=7898) | 62.1 | [3](https://blast.ncbi.nlm.nih.gov/Blast.cgi) |
| *..*[*Acipenser baerii baerii*](https://www.ncbi.nlm.nih.gov/Taxonomy/Browser/wwwtax.cgi?id=101767) | [bony fishes](https://www.ncbi.nlm.nih.gov/Taxonomy/Browser/wwwtax.cgi?id=7898) | 62.1 | [2](https://blast.ncbi.nlm.nih.gov/Blast.cgi) |
| *.*[*Squatina dumeril*](https://www.ncbi.nlm.nih.gov/Taxonomy/Browser/wwwtax.cgi?id=303946) | [sharks & rays](https://www.ncbi.nlm.nih.gov/Taxonomy/Browser/wwwtax.cgi?id=7778) | 65.8 | [2](https://blast.ncbi.nlm.nih.gov/Blast.cgi) |
| *.*[*Heterodontus zebra*](https://www.ncbi.nlm.nih.gov/Taxonomy/Browser/wwwtax.cgi?id=1213687) | [sharks & rays](https://www.ncbi.nlm.nih.gov/Taxonomy/Browser/wwwtax.cgi?id=7778) | 65.8 | [2](https://blast.ncbi.nlm.nih.gov/Blast.cgi) |
| *.*[*Aetobatus narinari*](https://www.ncbi.nlm.nih.gov/Taxonomy/Browser/wwwtax.cgi?id=87140) | [sharks & rays](https://www.ncbi.nlm.nih.gov/Taxonomy/Browser/wwwtax.cgi?id=7778) | 62.1 | [44](https://blast.ncbi.nlm.nih.gov/Blast.cgi) |
| *.*[*Aetobatus ocellatus*](https://www.ncbi.nlm.nih.gov/Taxonomy/Browser/wwwtax.cgi?id=1053712) | [sharks & rays](https://www.ncbi.nlm.nih.gov/Taxonomy/Browser/wwwtax.cgi?id=7778) | 62.1 | [1](https://blast.ncbi.nlm.nih.gov/Blast.cgi) |
| *.*[*Squatina japonica*](https://www.ncbi.nlm.nih.gov/Taxonomy/Browser/wwwtax.cgi?id=661037) | [sharks & rays](https://www.ncbi.nlm.nih.gov/Taxonomy/Browser/wwwtax.cgi?id=7778) | 60.2 | [2](https://blast.ncbi.nlm.nih.gov/Blast.cgi) |
| *.*[*Heterodontus francisci*](https://www.ncbi.nlm.nih.gov/Taxonomy/Browser/wwwtax.cgi?id=7792) | [sharks & rays](https://www.ncbi.nlm.nih.gov/Taxonomy/Browser/wwwtax.cgi?id=7778) | 56.5 | [3](https://blast.ncbi.nlm.nih.gov/Blast.cgi) |
| CYB used Target sequence (102 bp): CTGAATTTTAGTAGCGGACCTTATTACACTTACATGAATTGGAGGCCAACCAGTTGAACACCCCTACATTATTATTGGACAACTAGCTTCCATCCTATATTT (source: GENEBANK Acc. N. LR585431) | | | | |

| Table S1 c: In silico test for *G. pyrenaicus.*  *Number Hits:* Sequences producing Significant alignment (coverage (90-100), percent identity (85-100) | | | | |
| --- | --- | --- | --- | --- |
| ***Organism*** | **Blast Name** | **Score** | **Number Hits** | **Percent identity** |
| [*Eutheria*](https://www.ncbi.nlm.nih.gov/Taxonomy/Browser/wwwtax.cgi?id=9347) | [placentals](https://www.ncbi.nlm.nih.gov/Taxonomy/Browser/wwwtax.cgi?id=9347) |  | [5522](https://blast.ncbi.nlm.nih.gov/Blast.cgi) |  |
| *.*[*Boreoeutheria*](https://www.ncbi.nlm.nih.gov/Taxonomy/Browser/wwwtax.cgi?id=1437010) | [placentals](https://www.ncbi.nlm.nih.gov/Taxonomy/Browser/wwwtax.cgi?id=9347) |  | [5514](https://blast.ncbi.nlm.nih.gov/Blast.cgi) |  |
| *..*[*Laurasiatheria*](https://www.ncbi.nlm.nih.gov/Taxonomy/Browser/wwwtax.cgi?id=314145) | [placentals](https://www.ncbi.nlm.nih.gov/Taxonomy/Browser/wwwtax.cgi?id=9347) |  | [4945](https://blast.ncbi.nlm.nih.gov/Blast.cgi) |  |
| *...*[*Eulipotyphla*](https://www.ncbi.nlm.nih.gov/Taxonomy/Browser/wwwtax.cgi?id=9362) | [insectivores](https://www.ncbi.nlm.nih.gov/Taxonomy/Browser/wwwtax.cgi?id=9362) |  | [227](https://blast.ncbi.nlm.nih.gov/Blast.cgi) |  |
| *....*[*Talpidae*](https://www.ncbi.nlm.nih.gov/Taxonomy/Browser/wwwtax.cgi?id=9373) | [insectivores](https://www.ncbi.nlm.nih.gov/Taxonomy/Browser/wwwtax.cgi?id=9362) |  | [159](https://blast.ncbi.nlm.nih.gov/Blast.cgi) |  |
| *.....*[*Galemys pyrenaicus*](https://www.ncbi.nlm.nih.gov/Taxonomy/Browser/wwwtax.cgi?id=202257) | [insectivores](https://www.ncbi.nlm.nih.gov/Taxonomy/Browser/wwwtax.cgi?id=9362) | 261 | [4](https://blast.ncbi.nlm.nih.gov/Blast.cgi) | 100 |
| *.....*[*Condylura cristata*](https://www.ncbi.nlm.nih.gov/Taxonomy/Browser/wwwtax.cgi?id=143302) | [insectivores](https://www.ncbi.nlm.nih.gov/Taxonomy/Browser/wwwtax.cgi?id=9362) | 207 | [4](https://blast.ncbi.nlm.nih.gov/Blast.cgi) | < 93,63 |
| *.....*[*Mogera robusta*](https://www.ncbi.nlm.nih.gov/Taxonomy/Browser/wwwtax.cgi?id=111428) | [insectivores](https://www.ncbi.nlm.nih.gov/Taxonomy/Browser/wwwtax.cgi?id=9362) | 195 | [3](https://blast.ncbi.nlm.nih.gov/Blast.cgi) |
| *.....*[*Scalopus aquaticus*](https://www.ncbi.nlm.nih.gov/Taxonomy/Browser/wwwtax.cgi?id=71119) | [insectivores](https://www.ncbi.nlm.nih.gov/Taxonomy/Browser/wwwtax.cgi?id=9362) | 193 | [1](https://blast.ncbi.nlm.nih.gov/Blast.cgi) |
| *.....*[*Scaptochirus moschatus*](https://www.ncbi.nlm.nih.gov/Taxonomy/Browser/wwwtax.cgi?id=446353) | [insectivores](https://www.ncbi.nlm.nih.gov/Taxonomy/Browser/wwwtax.cgi?id=9362) | 191 | [4](https://blast.ncbi.nlm.nih.gov/Blast.cgi) |
| *.....Mogera sp.* RZX-2022 | [insectivores](https://www.ncbi.nlm.nih.gov/Taxonomy/Browser/wwwtax.cgi?id=9362) | 189 | [1](https://blast.ncbi.nlm.nih.gov/Blast.cgi) |
| *.....*[*Mogera hainana*](https://www.ncbi.nlm.nih.gov/Taxonomy/Browser/wwwtax.cgi?id=2804713) | [insectivores](https://www.ncbi.nlm.nih.gov/Taxonomy/Browser/wwwtax.cgi?id=9362) | 189 | [2](https://blast.ncbi.nlm.nih.gov/Blast.cgi) |
| *.....*[*Mogera wogura*](https://www.ncbi.nlm.nih.gov/Taxonomy/Browser/wwwtax.cgi?id=62295) | [insectivores](https://www.ncbi.nlm.nih.gov/Taxonomy/Browser/wwwtax.cgi?id=9362) | 185 | [4](https://blast.ncbi.nlm.nih.gov/Blast.cgi) |
| *.....*[*Mogera insularis insularis*](https://www.ncbi.nlm.nih.gov/Taxonomy/Browser/wwwtax.cgi?id=2909291) | [insectivores](https://www.ncbi.nlm.nih.gov/Taxonomy/Browser/wwwtax.cgi?id=9362) | 183 | [1](https://blast.ncbi.nlm.nih.gov/Blast.cgi) |
| *.....*[*Mogera imaizumii*](https://www.ncbi.nlm.nih.gov/Taxonomy/Browser/wwwtax.cgi?id=114415) | [insectivores](https://www.ncbi.nlm.nih.gov/Taxonomy/Browser/wwwtax.cgi?id=9362) | 183 | [2](https://blast.ncbi.nlm.nih.gov/Blast.cgi) |
| *.....*[*Mogera tokudae*](https://www.ncbi.nlm.nih.gov/Taxonomy/Browser/wwwtax.cgi?id=114414) | [insectivores](https://www.ncbi.nlm.nih.gov/Taxonomy/Browser/wwwtax.cgi?id=9362) | 180 | [1](https://blast.ncbi.nlm.nih.gov/Blast.cgi) |
| *.....*[*Urotrichus talpoides*](https://www.ncbi.nlm.nih.gov/Taxonomy/Browser/wwwtax.cgi?id=106106) | [insectivores](https://www.ncbi.nlm.nih.gov/Taxonomy/Browser/wwwtax.cgi?id=9362) | 180 | [4](https://blast.ncbi.nlm.nih.gov/Blast.cgi) |
| *.....*[*Scaptonyx fusicaudus*](https://www.ncbi.nlm.nih.gov/Taxonomy/Browser/wwwtax.cgi?id=224955) | [insectivores](https://www.ncbi.nlm.nih.gov/Taxonomy/Browser/wwwtax.cgi?id=9362) | 176 | [2](https://blast.ncbi.nlm.nih.gov/Blast.cgi) |
| *.....*[*Parascalops breweri*](https://www.ncbi.nlm.nih.gov/Taxonomy/Browser/wwwtax.cgi?id=94437) | [insectivores](https://www.ncbi.nlm.nih.gov/Taxonomy/Browser/wwwtax.cgi?id=9362) | 176 | [1](https://blast.ncbi.nlm.nih.gov/Blast.cgi) |
| *.....*[*Mogera insularis latouchei*](https://www.ncbi.nlm.nih.gov/Taxonomy/Browser/wwwtax.cgi?id=1340034) | [insectivores](https://www.ncbi.nlm.nih.gov/Taxonomy/Browser/wwwtax.cgi?id=9362) | 174 | [13](https://blast.ncbi.nlm.nih.gov/Blast.cgi) |
| *.....*[*Euroscaptor subanura*](https://www.ncbi.nlm.nih.gov/Taxonomy/Browser/wwwtax.cgi?id=1581555) | [insectivores](https://www.ncbi.nlm.nih.gov/Taxonomy/Browser/wwwtax.cgi?id=9362) | 172 | [16](https://blast.ncbi.nlm.nih.gov/Blast.cgi) |
| *.....*[*Mogera insularis*](https://www.ncbi.nlm.nih.gov/Taxonomy/Browser/wwwtax.cgi?id=114413) | [insectivores](https://www.ncbi.nlm.nih.gov/Taxonomy/Browser/wwwtax.cgi?id=9362) | 172 | [12](https://blast.ncbi.nlm.nih.gov/Blast.cgi) |
| *.....*[*Talpa europaea*](https://www.ncbi.nlm.nih.gov/Taxonomy/Browser/wwwtax.cgi?id=9375) | [insectivores](https://www.ncbi.nlm.nih.gov/Taxonomy/Browser/wwwtax.cgi?id=9362) | 171 | [3](https://blast.ncbi.nlm.nih.gov/Blast.cgi) |
| *.....*[*Parascaptor leucura*](https://www.ncbi.nlm.nih.gov/Taxonomy/Browser/wwwtax.cgi?id=1300119) | [insectivores](https://www.ncbi.nlm.nih.gov/Taxonomy/Browser/wwwtax.cgi?id=9362) | 171 | [5](https://blast.ncbi.nlm.nih.gov/Blast.cgi) |
| *.....*[*Oreoscaptor mizura*](https://www.ncbi.nlm.nih.gov/Taxonomy/Browser/wwwtax.cgi?id=2835217) | [insectivores](https://www.ncbi.nlm.nih.gov/Taxonomy/Browser/wwwtax.cgi?id=9362) | 169 | [6](https://blast.ncbi.nlm.nih.gov/Blast.cgi) |
| *.....*[*Talpa aquitania*](https://www.ncbi.nlm.nih.gov/Taxonomy/Browser/wwwtax.cgi?id=2615124) | [insectivores](https://www.ncbi.nlm.nih.gov/Taxonomy/Browser/wwwtax.cgi?id=9362) | 169 | [2](https://blast.ncbi.nlm.nih.gov/Blast.cgi) |
| *.....*[*Mogera kanoana*](https://www.ncbi.nlm.nih.gov/Taxonomy/Browser/wwwtax.cgi?id=468912) | [insectivores](https://www.ncbi.nlm.nih.gov/Taxonomy/Browser/wwwtax.cgi?id=9362) | 169 | [13](https://blast.ncbi.nlm.nih.gov/Blast.cgi) |
| *.....*[*Talpa altaica*](https://www.ncbi.nlm.nih.gov/Taxonomy/Browser/wwwtax.cgi?id=114409) | [insectivores](https://www.ncbi.nlm.nih.gov/Taxonomy/Browser/wwwtax.cgi?id=9362) | 165 | [1](https://blast.ncbi.nlm.nih.gov/Blast.cgi) |
| *.....*[*Talpa occidentalis*](https://www.ncbi.nlm.nih.gov/Taxonomy/Browser/wwwtax.cgi?id=50954) | [insectivores](https://www.ncbi.nlm.nih.gov/Taxonomy/Browser/wwwtax.cgi?id=9362) | 163 | [2](https://blast.ncbi.nlm.nih.gov/Blast.cgi) |
| *.....*[*Euroscaptor longirostris*](https://www.ncbi.nlm.nih.gov/Taxonomy/Browser/wwwtax.cgi?id=481574) | [insectivores](https://www.ncbi.nlm.nih.gov/Taxonomy/Browser/wwwtax.cgi?id=9362) | 163 | [9](https://blast.ncbi.nlm.nih.gov/Blast.cgi) |
| *.....*[*Uropsilus andersoni*](https://www.ncbi.nlm.nih.gov/Taxonomy/Browser/wwwtax.cgi?id=182668) | [insectivores](https://www.ncbi.nlm.nih.gov/Taxonomy/Browser/wwwtax.cgi?id=9362) | 161 | [3](https://blast.ncbi.nlm.nih.gov/Blast.cgi) |
| *.....*[*Desmana moschata*](https://www.ncbi.nlm.nih.gov/Taxonomy/Browser/wwwtax.cgi?id=182682) | [insectivores](https://www.ncbi.nlm.nih.gov/Taxonomy/Browser/wwwtax.cgi?id=9362) | 161 | [1](https://blast.ncbi.nlm.nih.gov/Blast.cgi) |
| *.....*[*Scapanulus oweni*](https://www.ncbi.nlm.nih.gov/Taxonomy/Browser/wwwtax.cgi?id=1300117) | [insectivores](https://www.ncbi.nlm.nih.gov/Taxonomy/Browser/wwwtax.cgi?id=9362) | 161 | [2](https://blast.ncbi.nlm.nih.gov/Blast.cgi) |
| *.....*[*Uropsilus investigator*](https://www.ncbi.nlm.nih.gov/Taxonomy/Browser/wwwtax.cgi?id=182670) | [insectivores](https://www.ncbi.nlm.nih.gov/Taxonomy/Browser/wwwtax.cgi?id=9362) | 161 | [6](https://blast.ncbi.nlm.nih.gov/Blast.cgi) |
| *.....*[*Euroscaptor parvidens*](https://www.ncbi.nlm.nih.gov/Taxonomy/Browser/wwwtax.cgi?id=1340036) | [insectivores](https://www.ncbi.nlm.nih.gov/Taxonomy/Browser/wwwtax.cgi?id=9362) | 158 | [6](https://blast.ncbi.nlm.nih.gov/Blast.cgi) |
| *.....*[*Uropsilus gracilis*](https://www.ncbi.nlm.nih.gov/Taxonomy/Browser/wwwtax.cgi?id=182669) | [insectivores](https://www.ncbi.nlm.nih.gov/Taxonomy/Browser/wwwtax.cgi?id=9362) | 154 | [2](https://blast.ncbi.nlm.nih.gov/Blast.cgi) |
| *.....*[*Euroscaptor klossi*](https://www.ncbi.nlm.nih.gov/Taxonomy/Browser/wwwtax.cgi?id=1340169) | [insectivores](https://www.ncbi.nlm.nih.gov/Taxonomy/Browser/wwwtax.cgi?id=9362) | 152 | [2](https://blast.ncbi.nlm.nih.gov/Blast.cgi) |
| *.....*[*Euroscaptor malayana*](https://www.ncbi.nlm.nih.gov/Taxonomy/Browser/wwwtax.cgi?id=533184) | [insectivores](https://www.ncbi.nlm.nih.gov/Taxonomy/Browser/wwwtax.cgi?id=9362) | 152 | [2](https://blast.ncbi.nlm.nih.gov/Blast.cgi) |
| *.....*[*Uropsilus nivatus*](https://www.ncbi.nlm.nih.gov/Taxonomy/Browser/wwwtax.cgi?id=1415659) | [insectivores](https://www.ncbi.nlm.nih.gov/Taxonomy/Browser/wwwtax.cgi?id=9362) | 150 | [14](https://blast.ncbi.nlm.nih.gov/Blast.cgi) |
| *.....Uropsilus sp.* 1 FT-2014 | [insectivores](https://www.ncbi.nlm.nih.gov/Taxonomy/Browser/wwwtax.cgi?id=9362) | 150 | [2](https://blast.ncbi.nlm.nih.gov/Blast.cgi) |
| *.....*[*Uropsilus aequodonenia*](https://www.ncbi.nlm.nih.gov/Taxonomy/Browser/wwwtax.cgi?id=1171130) | [insectivores](https://www.ncbi.nlm.nih.gov/Taxonomy/Browser/wwwtax.cgi?id=9362) | 150 | [1](https://blast.ncbi.nlm.nih.gov/Blast.cgi) |
| *.....*[*Uropsilus soricipes*](https://www.ncbi.nlm.nih.gov/Taxonomy/Browser/wwwtax.cgi?id=182671) | [insectivores](https://www.ncbi.nlm.nih.gov/Taxonomy/Browser/wwwtax.cgi?id=9362) | 150 | [1](https://blast.ncbi.nlm.nih.gov/Blast.cgi) |
| *.....Uropsilus sp.* CCJD_2002 | [insectivores](https://www.ncbi.nlm.nih.gov/Taxonomy/Browser/wwwtax.cgi?id=9362) | 150 | [1](https://blast.ncbi.nlm.nih.gov/Blast.cgi) |
| *....*[*Myosorex kihaulei*](https://www.ncbi.nlm.nih.gov/Taxonomy/Browser/wwwtax.cgi?id=307108) | [insectivores](https://www.ncbi.nlm.nih.gov/Taxonomy/Browser/wwwtax.cgi?id=9362) | 183 | [1](https://blast.ncbi.nlm.nih.gov/Blast.cgi) |
| *....*[*Sorex cinereus*](https://www.ncbi.nlm.nih.gov/Taxonomy/Browser/wwwtax.cgi?id=36803) | [insectivores](https://www.ncbi.nlm.nih.gov/Taxonomy/Browser/wwwtax.cgi?id=9362) | 174 | [1](https://blast.ncbi.nlm.nih.gov/Blast.cgi) |
| *....*[*Crocidura tanakae*](https://www.ncbi.nlm.nih.gov/Taxonomy/Browser/wwwtax.cgi?id=685768) | [insectivores](https://www.ncbi.nlm.nih.gov/Taxonomy/Browser/wwwtax.cgi?id=9362) | 169 | [3](https://blast.ncbi.nlm.nih.gov/Blast.cgi) |
| *....*[*Sorex thibetanus*](https://www.ncbi.nlm.nih.gov/Taxonomy/Browser/wwwtax.cgi?id=1343854) | [insectivores](https://www.ncbi.nlm.nih.gov/Taxonomy/Browser/wwwtax.cgi?id=9362) | 163 | [2](https://blast.ncbi.nlm.nih.gov/Blast.cgi) |
| *....*[*Anourosorex yamashinai*](https://www.ncbi.nlm.nih.gov/Taxonomy/Browser/wwwtax.cgi?id=268756) | [insectivores](https://www.ncbi.nlm.nih.gov/Taxonomy/Browser/wwwtax.cgi?id=9362) | 163 | [1](https://blast.ncbi.nlm.nih.gov/Blast.cgi) |
| *....*[*Anourosorex squamipes*](https://www.ncbi.nlm.nih.gov/Taxonomy/Browser/wwwtax.cgi?id=127560) | [insectivores](https://www.ncbi.nlm.nih.gov/Taxonomy/Browser/wwwtax.cgi?id=9362) | 163 | [1](https://blast.ncbi.nlm.nih.gov/Blast.cgi) |
| *....*[*Blarinella quadraticauda*](https://www.ncbi.nlm.nih.gov/Taxonomy/Browser/wwwtax.cgi?id=1159882) | [insectivores](https://www.ncbi.nlm.nih.gov/Taxonomy/Browser/wwwtax.cgi?id=9362) | 161 | [2](https://blast.ncbi.nlm.nih.gov/Blast.cgi) |
| *....*[*Sorex gracillimus*](https://www.ncbi.nlm.nih.gov/Taxonomy/Browser/wwwtax.cgi?id=62273) | [insectivores](https://www.ncbi.nlm.nih.gov/Taxonomy/Browser/wwwtax.cgi?id=9362) | 158 | [2](https://blast.ncbi.nlm.nih.gov/Blast.cgi) |
| *....*[*Sorex caecutiens*](https://www.ncbi.nlm.nih.gov/Taxonomy/Browser/wwwtax.cgi?id=62276) | [insectivores](https://www.ncbi.nlm.nih.gov/Taxonomy/Browser/wwwtax.cgi?id=9362) | 158 | [2](https://blast.ncbi.nlm.nih.gov/Blast.cgi) |
| *....Blarinella cf. quadraticauda* KIZ AL11069 | [insectivores](https://www.ncbi.nlm.nih.gov/Taxonomy/Browser/wwwtax.cgi?id=9362) | 156 | [2](https://blast.ncbi.nlm.nih.gov/Blast.cgi) |
| *....*[*Episoriculus macrurus*](https://www.ncbi.nlm.nih.gov/Taxonomy/Browser/wwwtax.cgi?id=268760) | [insectivores](https://www.ncbi.nlm.nih.gov/Taxonomy/Browser/wwwtax.cgi?id=9362) | 156 | [6](https://blast.ncbi.nlm.nih.gov/Blast.cgi) |
| *....*[*Pantherina griselda*](https://www.ncbi.nlm.nih.gov/Taxonomy/Browser/wwwtax.cgi?id=2588392) | [insectivores](https://www.ncbi.nlm.nih.gov/Taxonomy/Browser/wwwtax.cgi?id=9362) | 156 | [5](https://blast.ncbi.nlm.nih.gov/Blast.cgi) |
| *....*[*Sorex sinalis*](https://www.ncbi.nlm.nih.gov/Taxonomy/Browser/wwwtax.cgi?id=2083004) | [insectivores](https://www.ncbi.nlm.nih.gov/Taxonomy/Browser/wwwtax.cgi?id=9362) | 152 | [2](https://blast.ncbi.nlm.nih.gov/Blast.cgi) |
| *....*[*Sorex roboratus*](https://www.ncbi.nlm.nih.gov/Taxonomy/Browser/wwwtax.cgi?id=62288) | [insectivores](https://www.ncbi.nlm.nih.gov/Taxonomy/Browser/wwwtax.cgi?id=9362) | 152 | [2](https://blast.ncbi.nlm.nih.gov/Blast.cgi) |
| *....*[*Episoriculus caudatus*](https://www.ncbi.nlm.nih.gov/Taxonomy/Browser/wwwtax.cgi?id=62291) | [insectivores](https://www.ncbi.nlm.nih.gov/Taxonomy/Browser/wwwtax.cgi?id=9362) | 152 | [8](https://blast.ncbi.nlm.nih.gov/Blast.cgi) |
| *....*[*Sorex palustris*](https://www.ncbi.nlm.nih.gov/Taxonomy/Browser/wwwtax.cgi?id=46219) | [insectivores](https://www.ncbi.nlm.nih.gov/Taxonomy/Browser/wwwtax.cgi?id=9362) | 152 | [1](https://blast.ncbi.nlm.nih.gov/Blast.cgi) |
| *....*[*Neomys fodiens*](https://www.ncbi.nlm.nih.gov/Taxonomy/Browser/wwwtax.cgi?id=62282) | [insectivores](https://www.ncbi.nlm.nih.gov/Taxonomy/Browser/wwwtax.cgi?id=9362) | 148 | [5](https://blast.ncbi.nlm.nih.gov/Blast.cgi) |
| *....*[*Chodsigoa hypsibia*](https://www.ncbi.nlm.nih.gov/Taxonomy/Browser/wwwtax.cgi?id=862699) | [insectivores](https://www.ncbi.nlm.nih.gov/Taxonomy/Browser/wwwtax.cgi?id=9362) | 148 | [5](https://blast.ncbi.nlm.nih.gov/Blast.cgi) |
| *....*[*Chodsigoa parva*](https://www.ncbi.nlm.nih.gov/Taxonomy/Browser/wwwtax.cgi?id=2023227) | [insectivores](https://www.ncbi.nlm.nih.gov/Taxonomy/Browser/wwwtax.cgi?id=9362) | 148 | [2](https://blast.ncbi.nlm.nih.gov/Blast.cgi) |
| *....*[*Sorex isodon*](https://www.ncbi.nlm.nih.gov/Taxonomy/Browser/wwwtax.cgi?id=62281) | [insectivores](https://www.ncbi.nlm.nih.gov/Taxonomy/Browser/wwwtax.cgi?id=9362) | 147 | [1](https://blast.ncbi.nlm.nih.gov/Blast.cgi) |
| *....*[*Episoriculus umbrinus*](https://www.ncbi.nlm.nih.gov/Taxonomy/Browser/wwwtax.cgi?id=2175806) | [insectivores](https://www.ncbi.nlm.nih.gov/Taxonomy/Browser/wwwtax.cgi?id=9362) | 147 | [2](https://blast.ncbi.nlm.nih.gov/Blast.cgi) |
| *....*[*Neomys anomalus*](https://www.ncbi.nlm.nih.gov/Taxonomy/Browser/wwwtax.cgi?id=52814) | [insectivores](https://www.ncbi.nlm.nih.gov/Taxonomy/Browser/wwwtax.cgi?id=9362) | 147 | [1](https://blast.ncbi.nlm.nih.gov/Blast.cgi) |
| *....*[*Chodsigoa smithii*](https://www.ncbi.nlm.nih.gov/Taxonomy/Browser/wwwtax.cgi?id=1868624) | [insectivores](https://www.ncbi.nlm.nih.gov/Taxonomy/Browser/wwwtax.cgi?id=9362) | 147 | [1](https://blast.ncbi.nlm.nih.gov/Blast.cgi) |
| *....*[*Chodsigoa sodalis*](https://www.ncbi.nlm.nih.gov/Taxonomy/Browser/wwwtax.cgi?id=257447) | [insectivores](https://www.ncbi.nlm.nih.gov/Taxonomy/Browser/wwwtax.cgi?id=9362) | 147 | [2](https://blast.ncbi.nlm.nih.gov/Blast.cgi) |
| *....*[*Hylomys suillus maxi*](https://www.ncbi.nlm.nih.gov/Taxonomy/Browser/wwwtax.cgi?id=48900) | [insectivores](https://www.ncbi.nlm.nih.gov/Taxonomy/Browser/wwwtax.cgi?id=9362) | 141 | [2](https://blast.ncbi.nlm.nih.gov/Blast.cgi) |
| *....*[*Episoriculus leucops*](https://www.ncbi.nlm.nih.gov/Taxonomy/Browser/wwwtax.cgi?id=862700) | [insectivores](https://www.ncbi.nlm.nih.gov/Taxonomy/Browser/wwwtax.cgi?id=9362) | 141 | [6](https://blast.ncbi.nlm.nih.gov/Blast.cgi) |
| *...*[*Casinycteris argynnis*](https://www.ncbi.nlm.nih.gov/Taxonomy/Browser/wwwtax.cgi?id=1091509) | [bats](https://www.ncbi.nlm.nih.gov/Taxonomy/Browser/wwwtax.cgi?id=9397) | 174 | [5](https://blast.ncbi.nlm.nih.gov/Blast.cgi) |
| *...*[*Boneia bidens*](https://www.ncbi.nlm.nih.gov/Taxonomy/Browser/wwwtax.cgi?id=270782) | [bats](https://www.ncbi.nlm.nih.gov/Taxonomy/Browser/wwwtax.cgi?id=9397) | 172 | [1](https://blast.ncbi.nlm.nih.gov/Blast.cgi) |
| *...*[*Myotragus balearicus*](https://www.ncbi.nlm.nih.gov/Taxonomy/Browser/wwwtax.cgi?id=201717) | [even-toed ungulates](https://www.ncbi.nlm.nih.gov/Taxonomy/Browser/wwwtax.cgi?id=91561) | 171 | [9](https://blast.ncbi.nlm.nih.gov/Blast.cgi) |
| *...*[*Rousettus leschenaultii*](https://www.ncbi.nlm.nih.gov/Taxonomy/Browser/wwwtax.cgi?id=9408) | [bats](https://www.ncbi.nlm.nih.gov/Taxonomy/Browser/wwwtax.cgi?id=9397) | 169 | [6](https://blast.ncbi.nlm.nih.gov/Blast.cgi) |
| *...*[*Pteropus conspicillatus*](https://www.ncbi.nlm.nih.gov/Taxonomy/Browser/wwwtax.cgi?id=328804) | [bats](https://www.ncbi.nlm.nih.gov/Taxonomy/Browser/wwwtax.cgi?id=9397) | 167 | [6](https://blast.ncbi.nlm.nih.gov/Blast.cgi) |
| *...*[*Pteropus pohlei*](https://www.ncbi.nlm.nih.gov/Taxonomy/Browser/wwwtax.cgi?id=170229) | [bats](https://www.ncbi.nlm.nih.gov/Taxonomy/Browser/wwwtax.cgi?id=9397) | 167 | [1](https://blast.ncbi.nlm.nih.gov/Blast.cgi) |
| *...*[*Pteropus griseus*](https://www.ncbi.nlm.nih.gov/Taxonomy/Browser/wwwtax.cgi?id=1495907) | [bats](https://www.ncbi.nlm.nih.gov/Taxonomy/Browser/wwwtax.cgi?id=9397) | 167 | [1](https://blast.ncbi.nlm.nih.gov/Blast.cgi) |
| *...*[*Pteropus lombocensis*](https://www.ncbi.nlm.nih.gov/Taxonomy/Browser/wwwtax.cgi?id=1495904) | [bats](https://www.ncbi.nlm.nih.gov/Taxonomy/Browser/wwwtax.cgi?id=9397) | 167 | [1](https://blast.ncbi.nlm.nih.gov/Blast.cgi) |
| *...*[*Pteropus mariannus*](https://www.ncbi.nlm.nih.gov/Taxonomy/Browser/wwwtax.cgi?id=522447) | [bats](https://www.ncbi.nlm.nih.gov/Taxonomy/Browser/wwwtax.cgi?id=9397) | 167 | [1](https://blast.ncbi.nlm.nih.gov/Blast.cgi) |
| *...*[*Pteropus pelewensis pelewensis*](https://www.ncbi.nlm.nih.gov/Taxonomy/Browser/wwwtax.cgi?id=1495878) | [bats](https://www.ncbi.nlm.nih.gov/Taxonomy/Browser/wwwtax.cgi?id=9397) | 167 | [2](https://blast.ncbi.nlm.nih.gov/Blast.cgi) |
| *...*[*Pteropus alecto*](https://www.ncbi.nlm.nih.gov/Taxonomy/Browser/wwwtax.cgi?id=9402) | [bats](https://www.ncbi.nlm.nih.gov/Taxonomy/Browser/wwwtax.cgi?id=9397) | 167 | [3](https://blast.ncbi.nlm.nih.gov/Blast.cgi) |
| *...*[*Pteropus poliocephalus*](https://www.ncbi.nlm.nih.gov/Taxonomy/Browser/wwwtax.cgi?id=9403) | [bats](https://www.ncbi.nlm.nih.gov/Taxonomy/Browser/wwwtax.cgi?id=9397) | 167 | [3](https://blast.ncbi.nlm.nih.gov/Blast.cgi) |
| *...*[*Pteropus yapensis*](https://www.ncbi.nlm.nih.gov/Taxonomy/Browser/wwwtax.cgi?id=1495872) | [bats](https://www.ncbi.nlm.nih.gov/Taxonomy/Browser/wwwtax.cgi?id=9397) | 167 | [2](https://blast.ncbi.nlm.nih.gov/Blast.cgi) |
| *...*[*Eonycteris robusta*](https://www.ncbi.nlm.nih.gov/Taxonomy/Browser/wwwtax.cgi?id=1091510) | [bats](https://www.ncbi.nlm.nih.gov/Taxonomy/Browser/wwwtax.cgi?id=9397) | 167 | [1](https://blast.ncbi.nlm.nih.gov/Blast.cgi) |
| *...*[*Pteropus hypomelanus*](https://www.ncbi.nlm.nih.gov/Taxonomy/Browser/wwwtax.cgi?id=9405) | [bats](https://www.ncbi.nlm.nih.gov/Taxonomy/Browser/wwwtax.cgi?id=9397) | 167 | [8](https://blast.ncbi.nlm.nih.gov/Blast.cgi) |
| *...*[*Acerodon jubatus*](https://www.ncbi.nlm.nih.gov/Taxonomy/Browser/wwwtax.cgi?id=505845) | [bats](https://www.ncbi.nlm.nih.gov/Taxonomy/Browser/wwwtax.cgi?id=9397) | 167 | [1](https://blast.ncbi.nlm.nih.gov/Blast.cgi) |
| *...*[*Aproteles bulmerae*](https://www.ncbi.nlm.nih.gov/Taxonomy/Browser/wwwtax.cgi?id=58059) | [bats](https://www.ncbi.nlm.nih.gov/Taxonomy/Browser/wwwtax.cgi?id=9397) | 167 | [2](https://blast.ncbi.nlm.nih.gov/Blast.cgi) |
| *...*[*Eonycteris spelaea*](https://www.ncbi.nlm.nih.gov/Taxonomy/Browser/wwwtax.cgi?id=58065) | [bats](https://www.ncbi.nlm.nih.gov/Taxonomy/Browser/wwwtax.cgi?id=9397) | 167 | [13](https://blast.ncbi.nlm.nih.gov/Blast.cgi) |
| *...Dobsonia sp.* 050810x2F | [bats](https://www.ncbi.nlm.nih.gov/Taxonomy/Browser/wwwtax.cgi?id=9397) | 165 | [1](https://blast.ncbi.nlm.nih.gov/Blast.cgi) |
| *...*[*Capra aegagrus*](https://www.ncbi.nlm.nih.gov/Taxonomy/Browser/wwwtax.cgi?id=9923) | [even-toed ungulates](https://www.ncbi.nlm.nih.gov/Taxonomy/Browser/wwwtax.cgi?id=91561) | 165 | [17](https://blast.ncbi.nlm.nih.gov/Blast.cgi) |
| *...*[*Styloctenium wallacei*](https://www.ncbi.nlm.nih.gov/Taxonomy/Browser/wwwtax.cgi?id=1442611) | [bats](https://www.ncbi.nlm.nih.gov/Taxonomy/Browser/wwwtax.cgi?id=9397) | 165 | [1](https://blast.ncbi.nlm.nih.gov/Blast.cgi) |
| *...*[*Thoopterus nigrescens*](https://www.ncbi.nlm.nih.gov/Taxonomy/Browser/wwwtax.cgi?id=58087) | [bats](https://www.ncbi.nlm.nih.gov/Taxonomy/Browser/wwwtax.cgi?id=9397) | 165 | [3](https://blast.ncbi.nlm.nih.gov/Blast.cgi) |
| *...*[*Dobsonia viridis*](https://www.ncbi.nlm.nih.gov/Taxonomy/Browser/wwwtax.cgi?id=170218) | [bats](https://www.ncbi.nlm.nih.gov/Taxonomy/Browser/wwwtax.cgi?id=9397) | 165 | [2](https://blast.ncbi.nlm.nih.gov/Blast.cgi) |
| *...*[*Dobsonia moluccensis*](https://www.ncbi.nlm.nih.gov/Taxonomy/Browser/wwwtax.cgi?id=42147) | [bats](https://www.ncbi.nlm.nih.gov/Taxonomy/Browser/wwwtax.cgi?id=9397) | 165 | [4](https://blast.ncbi.nlm.nih.gov/Blast.cgi) |
| *...*[*Desmalopex leucopterus*](https://www.ncbi.nlm.nih.gov/Taxonomy/Browser/wwwtax.cgi?id=505943) | [bats](https://www.ncbi.nlm.nih.gov/Taxonomy/Browser/wwwtax.cgi?id=9397) | 165 | [3](https://blast.ncbi.nlm.nih.gov/Blast.cgi) |
| *...*[*Desmalopex microleucopterus*](https://www.ncbi.nlm.nih.gov/Taxonomy/Browser/wwwtax.cgi?id=515995) | [bats](https://www.ncbi.nlm.nih.gov/Taxonomy/Browser/wwwtax.cgi?id=9397) | 165 | [5](https://blast.ncbi.nlm.nih.gov/Blast.cgi) |
| *...*[*Platygonus compressus*](https://www.ncbi.nlm.nih.gov/Taxonomy/Browser/wwwtax.cgi?id=1983451) | [even-toed ungulates](https://www.ncbi.nlm.nih.gov/Taxonomy/Browser/wwwtax.cgi?id=91561) | 163 | [11](https://blast.ncbi.nlm.nih.gov/Blast.cgi) |
| *...*[*Macroglossus sobrinus*](https://www.ncbi.nlm.nih.gov/Taxonomy/Browser/wwwtax.cgi?id=326083) | [bats](https://www.ncbi.nlm.nih.gov/Taxonomy/Browser/wwwtax.cgi?id=9397) | 163 | [6](https://blast.ncbi.nlm.nih.gov/Blast.cgi) |
| *...*[*Ptenochirus wetmorei*](https://www.ncbi.nlm.nih.gov/Taxonomy/Browser/wwwtax.cgi?id=414524) | [bats](https://www.ncbi.nlm.nih.gov/Taxonomy/Browser/wwwtax.cgi?id=9397) | 163 | [4](https://blast.ncbi.nlm.nih.gov/Blast.cgi) |
| *...*[*Rousettus obliviosus*](https://www.ncbi.nlm.nih.gov/Taxonomy/Browser/wwwtax.cgi?id=705079) | [bats](https://www.ncbi.nlm.nih.gov/Taxonomy/Browser/wwwtax.cgi?id=9397) | 161 | [3](https://blast.ncbi.nlm.nih.gov/Blast.cgi) |
| *...*[*Rousettus madagascariensis*](https://www.ncbi.nlm.nih.gov/Taxonomy/Browser/wwwtax.cgi?id=77223) | [bats](https://www.ncbi.nlm.nih.gov/Taxonomy/Browser/wwwtax.cgi?id=9397) | 161 | [4](https://blast.ncbi.nlm.nih.gov/Blast.cgi) |
| *...*[*Rousettus aegyptiacus*](https://www.ncbi.nlm.nih.gov/Taxonomy/Browser/wwwtax.cgi?id=9407) | [bats](https://www.ncbi.nlm.nih.gov/Taxonomy/Browser/wwwtax.cgi?id=9397) | 161 | [11](https://blast.ncbi.nlm.nih.gov/Blast.cgi) |
| *...*[*Myonycteris torquata*](https://www.ncbi.nlm.nih.gov/Taxonomy/Browser/wwwtax.cgi?id=77243) | [bats](https://www.ncbi.nlm.nih.gov/Taxonomy/Browser/wwwtax.cgi?id=9397) | 161 | [4](https://blast.ncbi.nlm.nih.gov/Blast.cgi) |
| *...*[*Megaerops niphanae*](https://www.ncbi.nlm.nih.gov/Taxonomy/Browser/wwwtax.cgi?id=77233) | [bats](https://www.ncbi.nlm.nih.gov/Taxonomy/Browser/wwwtax.cgi?id=9397) | 161 | [2](https://blast.ncbi.nlm.nih.gov/Blast.cgi) |
| *...*[*Cynopterus brachyotis*](https://www.ncbi.nlm.nih.gov/Taxonomy/Browser/wwwtax.cgi?id=58060) | [bats](https://www.ncbi.nlm.nih.gov/Taxonomy/Browser/wwwtax.cgi?id=9397) | 161 | [11](https://blast.ncbi.nlm.nih.gov/Blast.cgi) |
| *...*[*Pteropus dasymallus formosus*](https://www.ncbi.nlm.nih.gov/Taxonomy/Browser/wwwtax.cgi?id=385575) | [bats](https://www.ncbi.nlm.nih.gov/Taxonomy/Browser/wwwtax.cgi?id=9397) | 161 | [2](https://blast.ncbi.nlm.nih.gov/Blast.cgi) |
| *...*[*Lonchophylla concava*](https://www.ncbi.nlm.nih.gov/Taxonomy/Browser/wwwtax.cgi?id=1303335) | [bats](https://www.ncbi.nlm.nih.gov/Taxonomy/Browser/wwwtax.cgi?id=9397) | 161 | [3](https://blast.ncbi.nlm.nih.gov/Blast.cgi) |
| *...*[*Nandinia binotata*](https://www.ncbi.nlm.nih.gov/Taxonomy/Browser/wwwtax.cgi?id=71115) | [carnivores](https://www.ncbi.nlm.nih.gov/Taxonomy/Browser/wwwtax.cgi?id=33554) | 161 | [24](https://blast.ncbi.nlm.nih.gov/Blast.cgi) |
| *...*[*Pteropus vetulus*](https://www.ncbi.nlm.nih.gov/Taxonomy/Browser/wwwtax.cgi?id=170231) | [bats](https://www.ncbi.nlm.nih.gov/Taxonomy/Browser/wwwtax.cgi?id=9397) | 161 | [2](https://blast.ncbi.nlm.nih.gov/Blast.cgi) |
| *...*[*Pteropus rayneri*](https://www.ncbi.nlm.nih.gov/Taxonomy/Browser/wwwtax.cgi?id=110942) | [bats](https://www.ncbi.nlm.nih.gov/Taxonomy/Browser/wwwtax.cgi?id=9397) | 161 | [3](https://blast.ncbi.nlm.nih.gov/Blast.cgi) |
| *...*[*Pteropus voeltzkowi*](https://www.ncbi.nlm.nih.gov/Taxonomy/Browser/wwwtax.cgi?id=589509) | [bats](https://www.ncbi.nlm.nih.gov/Taxonomy/Browser/wwwtax.cgi?id=9397) | 161 | [3](https://blast.ncbi.nlm.nih.gov/Blast.cgi) |
| *...*[*Pteropus rayneri grandis*](https://www.ncbi.nlm.nih.gov/Taxonomy/Browser/wwwtax.cgi?id=1496126) | [bats](https://www.ncbi.nlm.nih.gov/Taxonomy/Browser/wwwtax.cgi?id=9397) | 161 | [1](https://blast.ncbi.nlm.nih.gov/Blast.cgi) |
| *...*[*Pteropus neohibernicus*](https://www.ncbi.nlm.nih.gov/Taxonomy/Browser/wwwtax.cgi?id=170224) | [bats](https://www.ncbi.nlm.nih.gov/Taxonomy/Browser/wwwtax.cgi?id=9397) | 161 | [2](https://blast.ncbi.nlm.nih.gov/Blast.cgi) |
| *...*[*Pteropus anetianus eotinus*](https://www.ncbi.nlm.nih.gov/Taxonomy/Browser/wwwtax.cgi?id=1495874) | [bats](https://www.ncbi.nlm.nih.gov/Taxonomy/Browser/wwwtax.cgi?id=9397) | 161 | [1](https://blast.ncbi.nlm.nih.gov/Blast.cgi) |
| *...*[*Pecari tajacu*](https://www.ncbi.nlm.nih.gov/Taxonomy/Browser/wwwtax.cgi?id=9829) | [even-toed ungulates](https://www.ncbi.nlm.nih.gov/Taxonomy/Browser/wwwtax.cgi?id=91561) | 161 | [6](https://blast.ncbi.nlm.nih.gov/Blast.cgi) |
| *...*[*Rousettus spinalatus*](https://www.ncbi.nlm.nih.gov/Taxonomy/Browser/wwwtax.cgi?id=414523) | [bats](https://www.ncbi.nlm.nih.gov/Taxonomy/Browser/wwwtax.cgi?id=9397) | 161 | [3](https://blast.ncbi.nlm.nih.gov/Blast.cgi) |
| *...*[*Penthetor lucasi*](https://www.ncbi.nlm.nih.gov/Taxonomy/Browser/wwwtax.cgi?id=326151) | [bats](https://www.ncbi.nlm.nih.gov/Taxonomy/Browser/wwwtax.cgi?id=9397) | 161 | [2](https://blast.ncbi.nlm.nih.gov/Blast.cgi) |
| *...*[*Dyacopterus spadiceus*](https://www.ncbi.nlm.nih.gov/Taxonomy/Browser/wwwtax.cgi?id=326081) | [bats](https://www.ncbi.nlm.nih.gov/Taxonomy/Browser/wwwtax.cgi?id=9397) | 161 | [4](https://blast.ncbi.nlm.nih.gov/Blast.cgi) |
| *...*[*Rousettus lanosus*](https://www.ncbi.nlm.nih.gov/Taxonomy/Browser/wwwtax.cgi?id=77222) | [bats](https://www.ncbi.nlm.nih.gov/Taxonomy/Browser/wwwtax.cgi?id=9397) | 161 | [1](https://blast.ncbi.nlm.nih.gov/Blast.cgi) |
| *...*[*Rousettus amplexicaudatus*](https://www.ncbi.nlm.nih.gov/Taxonomy/Browser/wwwtax.cgi?id=58083) | [bats](https://www.ncbi.nlm.nih.gov/Taxonomy/Browser/wwwtax.cgi?id=9397) | 161 | [12](https://blast.ncbi.nlm.nih.gov/Blast.cgi) |
| *...*[*Pteropus dasymallus*](https://www.ncbi.nlm.nih.gov/Taxonomy/Browser/wwwtax.cgi?id=126282) | [bats](https://www.ncbi.nlm.nih.gov/Taxonomy/Browser/wwwtax.cgi?id=9397) | 161 | [2](https://blast.ncbi.nlm.nih.gov/Blast.cgi) |
| *...*[*Plerotes anchietae*](https://www.ncbi.nlm.nih.gov/Taxonomy/Browser/wwwtax.cgi?id=1812310) | [bats](https://www.ncbi.nlm.nih.gov/Taxonomy/Browser/wwwtax.cgi?id=9397) | 159 | [4](https://blast.ncbi.nlm.nih.gov/Blast.cgi) |
| *...*[*Epomops buettikoferi*](https://www.ncbi.nlm.nih.gov/Taxonomy/Browser/wwwtax.cgi?id=1410044) | [bats](https://www.ncbi.nlm.nih.gov/Taxonomy/Browser/wwwtax.cgi?id=9397) | 159 | [2](https://blast.ncbi.nlm.nih.gov/Blast.cgi) |
| *...*[*Capra hircus*](https://www.ncbi.nlm.nih.gov/Taxonomy/Browser/wwwtax.cgi?id=9925) | [even-toed ungulates](https://www.ncbi.nlm.nih.gov/Taxonomy/Browser/wwwtax.cgi?id=91561) | 159 | [289](https://blast.ncbi.nlm.nih.gov/Blast.cgi) |
| *...*[*Capra falconeri*](https://www.ncbi.nlm.nih.gov/Taxonomy/Browser/wwwtax.cgi?id=48167) | [even-toed ungulates](https://www.ncbi.nlm.nih.gov/Taxonomy/Browser/wwwtax.cgi?id=91561) | 159 | [5](https://blast.ncbi.nlm.nih.gov/Blast.cgi) |
| *...*[*Capra cylindricornis*](https://www.ncbi.nlm.nih.gov/Taxonomy/Browser/wwwtax.cgi?id=72541) | [even-toed ungulates](https://www.ncbi.nlm.nih.gov/Taxonomy/Browser/wwwtax.cgi?id=91561) | 159 | [5](https://blast.ncbi.nlm.nih.gov/Blast.cgi) |
| *...*[*Capra sibirica*](https://www.ncbi.nlm.nih.gov/Taxonomy/Browser/wwwtax.cgi?id=72544) | [even-toed ungulates](https://www.ncbi.nlm.nih.gov/Taxonomy/Browser/wwwtax.cgi?id=91561) | 159 | [5](https://blast.ncbi.nlm.nih.gov/Blast.cgi) |
| *...*[*Capra caucasica*](https://www.ncbi.nlm.nih.gov/Taxonomy/Browser/wwwtax.cgi?id=72540) | [even-toed ungulates](https://www.ncbi.nlm.nih.gov/Taxonomy/Browser/wwwtax.cgi?id=91561) | 159 | [2](https://blast.ncbi.nlm.nih.gov/Blast.cgi) |
| *...*[*Styloctenium mindorensis*](https://www.ncbi.nlm.nih.gov/Taxonomy/Browser/wwwtax.cgi?id=1091512) | [bats](https://www.ncbi.nlm.nih.gov/Taxonomy/Browser/wwwtax.cgi?id=9397) | 159 | [1](https://blast.ncbi.nlm.nih.gov/Blast.cgi) |
| *...*[*Dobsonia praedatrix*](https://www.ncbi.nlm.nih.gov/Taxonomy/Browser/wwwtax.cgi?id=170217) | [bats](https://www.ncbi.nlm.nih.gov/Taxonomy/Browser/wwwtax.cgi?id=9397) | 159 | [2](https://blast.ncbi.nlm.nih.gov/Blast.cgi) |
| *...*[*Dobsonia inermis*](https://www.ncbi.nlm.nih.gov/Taxonomy/Browser/wwwtax.cgi?id=170214) | [bats](https://www.ncbi.nlm.nih.gov/Taxonomy/Browser/wwwtax.cgi?id=9397) | 159 | [1](https://blast.ncbi.nlm.nih.gov/Blast.cgi) |
| *...*[*Canis lupus familiaris*](https://www.ncbi.nlm.nih.gov/Taxonomy/Browser/wwwtax.cgi?id=9615) | [carnivores](https://www.ncbi.nlm.nih.gov/Taxonomy/Browser/wwwtax.cgi?id=33554) | 158 | [3254](https://blast.ncbi.nlm.nih.gov/Blast.cgi) |
| *...*[*Sphaerias blanfordi*](https://www.ncbi.nlm.nih.gov/Taxonomy/Browser/wwwtax.cgi?id=662898) | [bats](https://www.ncbi.nlm.nih.gov/Taxonomy/Browser/wwwtax.cgi?id=9397) | 158 | [4](https://blast.ncbi.nlm.nih.gov/Blast.cgi) |
| *...*[*Casinycteris ophiodon*](https://www.ncbi.nlm.nih.gov/Taxonomy/Browser/wwwtax.cgi?id=1497438) | [bats](https://www.ncbi.nlm.nih.gov/Taxonomy/Browser/wwwtax.cgi?id=9397) | 158 | [4](https://blast.ncbi.nlm.nih.gov/Blast.cgi) |
| *...*[*Neotragus moschatus*](https://www.ncbi.nlm.nih.gov/Taxonomy/Browser/wwwtax.cgi?id=66442) | [even-toed ungulates](https://www.ncbi.nlm.nih.gov/Taxonomy/Browser/wwwtax.cgi?id=91561) | 158 | [2](https://blast.ncbi.nlm.nih.gov/Blast.cgi) |
| *...*[*Macroglossus minimus*](https://www.ncbi.nlm.nih.gov/Taxonomy/Browser/wwwtax.cgi?id=29076) | [bats](https://www.ncbi.nlm.nih.gov/Taxonomy/Browser/wwwtax.cgi?id=9397) | 158 | [4](https://blast.ncbi.nlm.nih.gov/Blast.cgi) |
| *...*[*Otopteropus cartilagonodus*](https://www.ncbi.nlm.nih.gov/Taxonomy/Browser/wwwtax.cgi?id=328968) | [bats](https://www.ncbi.nlm.nih.gov/Taxonomy/Browser/wwwtax.cgi?id=9397) | 158 | [2](https://blast.ncbi.nlm.nih.gov/Blast.cgi) |
| *...*[*Chironax melanocephalus*](https://www.ncbi.nlm.nih.gov/Taxonomy/Browser/wwwtax.cgi?id=170236) | [bats](https://www.ncbi.nlm.nih.gov/Taxonomy/Browser/wwwtax.cgi?id=9397) | 158 | [6](https://blast.ncbi.nlm.nih.gov/Blast.cgi) |
| *...*[*Cynopterus sphinx*](https://www.ncbi.nlm.nih.gov/Taxonomy/Browser/wwwtax.cgi?id=9400) | [bats](https://www.ncbi.nlm.nih.gov/Taxonomy/Browser/wwwtax.cgi?id=9397) | 156 | [7](https://blast.ncbi.nlm.nih.gov/Blast.cgi) |
| *...*[*Lissonycteris angolensis*](https://www.ncbi.nlm.nih.gov/Taxonomy/Browser/wwwtax.cgi?id=58071) | [bats](https://www.ncbi.nlm.nih.gov/Taxonomy/Browser/wwwtax.cgi?id=9397) | 156 | [6](https://blast.ncbi.nlm.nih.gov/Blast.cgi) |
| *...*[*Megaloglossus woermanni*](https://www.ncbi.nlm.nih.gov/Taxonomy/Browser/wwwtax.cgi?id=58073) | [bats](https://www.ncbi.nlm.nih.gov/Taxonomy/Browser/wwwtax.cgi?id=9397) | 156 | [8](https://blast.ncbi.nlm.nih.gov/Blast.cgi) |
| *...*[*Ovis nivicola lydekkeri*](https://www.ncbi.nlm.nih.gov/Taxonomy/Browser/wwwtax.cgi?id=1867112) | [even-toed ungulates](https://www.ncbi.nlm.nih.gov/Taxonomy/Browser/wwwtax.cgi?id=91561) | 156 | [2](https://blast.ncbi.nlm.nih.gov/Blast.cgi) |
| *...*[*Pteropus macrotis*](https://www.ncbi.nlm.nih.gov/Taxonomy/Browser/wwwtax.cgi?id=448077) | [bats](https://www.ncbi.nlm.nih.gov/Taxonomy/Browser/wwwtax.cgi?id=9397) | 156 | [1](https://blast.ncbi.nlm.nih.gov/Blast.cgi) |
| *...*[*Pteropus molossinus*](https://www.ncbi.nlm.nih.gov/Taxonomy/Browser/wwwtax.cgi?id=522446) | [bats](https://www.ncbi.nlm.nih.gov/Taxonomy/Browser/wwwtax.cgi?id=9397) | 156 | [2](https://blast.ncbi.nlm.nih.gov/Blast.cgi) |
| *...*[*Mephitis mephitis*](https://www.ncbi.nlm.nih.gov/Taxonomy/Browser/wwwtax.cgi?id=30548) | [carnivores](https://www.ncbi.nlm.nih.gov/Taxonomy/Browser/wwwtax.cgi?id=33554) | 156 | [5](https://blast.ncbi.nlm.nih.gov/Blast.cgi) |
| *...*[*Epomophorus pusillus*](https://www.ncbi.nlm.nih.gov/Taxonomy/Browser/wwwtax.cgi?id=2853157) | [bats](https://www.ncbi.nlm.nih.gov/Taxonomy/Browser/wwwtax.cgi?id=9397) | 156 | [4](https://blast.ncbi.nlm.nih.gov/Blast.cgi) |
| *...*[*Megaerops ecaudatus*](https://www.ncbi.nlm.nih.gov/Taxonomy/Browser/wwwtax.cgi?id=298110) | [bats](https://www.ncbi.nlm.nih.gov/Taxonomy/Browser/wwwtax.cgi?id=9397) | 156 | [3](https://blast.ncbi.nlm.nih.gov/Blast.cgi) |
| *...*[*Eonycteris major*](https://www.ncbi.nlm.nih.gov/Taxonomy/Browser/wwwtax.cgi?id=526811) | [bats](https://www.ncbi.nlm.nih.gov/Taxonomy/Browser/wwwtax.cgi?id=9397) | 156 | [4](https://blast.ncbi.nlm.nih.gov/Blast.cgi) |
| *...*[*Syconycteris australis*](https://www.ncbi.nlm.nih.gov/Taxonomy/Browser/wwwtax.cgi?id=58085) | [bats](https://www.ncbi.nlm.nih.gov/Taxonomy/Browser/wwwtax.cgi?id=9397) | 156 | [1](https://blast.ncbi.nlm.nih.gov/Blast.cgi) |
| *...*[*Epomops franqueti*](https://www.ncbi.nlm.nih.gov/Taxonomy/Browser/wwwtax.cgi?id=77231) | [bats](https://www.ncbi.nlm.nih.gov/Taxonomy/Browser/wwwtax.cgi?id=9397) | 154 | [6](https://blast.ncbi.nlm.nih.gov/Blast.cgi) |
| *...*[*Epomophorus gambianus*](https://www.ncbi.nlm.nih.gov/Taxonomy/Browser/wwwtax.cgi?id=372077) | [bats](https://www.ncbi.nlm.nih.gov/Taxonomy/Browser/wwwtax.cgi?id=9397) | 154 | [5](https://blast.ncbi.nlm.nih.gov/Blast.cgi) |
| *...*[*Ovis dalli*](https://www.ncbi.nlm.nih.gov/Taxonomy/Browser/wwwtax.cgi?id=9943) | [even-toed ungulates](https://www.ncbi.nlm.nih.gov/Taxonomy/Browser/wwwtax.cgi?id=91561) | 154 | [3](https://blast.ncbi.nlm.nih.gov/Blast.cgi) |
| *...*[*Ovis canadensis*](https://www.ncbi.nlm.nih.gov/Taxonomy/Browser/wwwtax.cgi?id=37174) | [even-toed ungulates](https://www.ncbi.nlm.nih.gov/Taxonomy/Browser/wwwtax.cgi?id=91561) | 154 | [3](https://blast.ncbi.nlm.nih.gov/Blast.cgi) |
| *...*[*Hemitragus hylocrius*](https://www.ncbi.nlm.nih.gov/Taxonomy/Browser/wwwtax.cgi?id=330464) | [even-toed ungulates](https://www.ncbi.nlm.nih.gov/Taxonomy/Browser/wwwtax.cgi?id=91561) | 154 | [1](https://blast.ncbi.nlm.nih.gov/Blast.cgi) |
| *...*[*Epomophorus wahlbergi*](https://www.ncbi.nlm.nih.gov/Taxonomy/Browser/wwwtax.cgi?id=58067) | [bats](https://www.ncbi.nlm.nih.gov/Taxonomy/Browser/wwwtax.cgi?id=9397) | 154 | [6](https://blast.ncbi.nlm.nih.gov/Blast.cgi) |
| *...*[*Vulpes vulpes*](https://www.ncbi.nlm.nih.gov/Taxonomy/Browser/wwwtax.cgi?id=9627) | [carnivores](https://www.ncbi.nlm.nih.gov/Taxonomy/Browser/wwwtax.cgi?id=33554) | 152 | [10](https://blast.ncbi.nlm.nih.gov/Blast.cgi) |
| *...*[*Vulpes zerda*](https://www.ncbi.nlm.nih.gov/Taxonomy/Browser/wwwtax.cgi?id=68732) | [carnivores](https://www.ncbi.nlm.nih.gov/Taxonomy/Browser/wwwtax.cgi?id=33554) | 152 | [3](https://blast.ncbi.nlm.nih.gov/Blast.cgi) |
| *...*[*Vulpes vulpes montana*](https://www.ncbi.nlm.nih.gov/Taxonomy/Browser/wwwtax.cgi?id=1398410) | [carnivores](https://www.ncbi.nlm.nih.gov/Taxonomy/Browser/wwwtax.cgi?id=33554) | 152 | [1](https://blast.ncbi.nlm.nih.gov/Blast.cgi) |
| *...*[*Cephalophus spadix*](https://www.ncbi.nlm.nih.gov/Taxonomy/Browser/wwwtax.cgi?id=129231) | [even-toed ungulates](https://www.ncbi.nlm.nih.gov/Taxonomy/Browser/wwwtax.cgi?id=91561) | 152 | [2](https://blast.ncbi.nlm.nih.gov/Blast.cgi) |
| *...*[*Nasua nasua*](https://www.ncbi.nlm.nih.gov/Taxonomy/Browser/wwwtax.cgi?id=9651) | [carnivores](https://www.ncbi.nlm.nih.gov/Taxonomy/Browser/wwwtax.cgi?id=33554) | 152 | [2](https://blast.ncbi.nlm.nih.gov/Blast.cgi) |
| *...*[*Nyctimene robinsoni*](https://www.ncbi.nlm.nih.gov/Taxonomy/Browser/wwwtax.cgi?id=58079) | [bats](https://www.ncbi.nlm.nih.gov/Taxonomy/Browser/wwwtax.cgi?id=9397) | 152 | [2](https://blast.ncbi.nlm.nih.gov/Blast.cgi) |
| *...*[*Aethalops alecto*](https://www.ncbi.nlm.nih.gov/Taxonomy/Browser/wwwtax.cgi?id=77227) | [bats](https://www.ncbi.nlm.nih.gov/Taxonomy/Browser/wwwtax.cgi?id=9397) | 152 | [2](https://blast.ncbi.nlm.nih.gov/Blast.cgi) |
| *...*[*Myonycteris relicta*](https://www.ncbi.nlm.nih.gov/Taxonomy/Browser/wwwtax.cgi?id=77242) | [bats](https://www.ncbi.nlm.nih.gov/Taxonomy/Browser/wwwtax.cgi?id=9397) | 150 | [2](https://blast.ncbi.nlm.nih.gov/Blast.cgi) |
| *...*[*Myonycteris leptodon*](https://www.ncbi.nlm.nih.gov/Taxonomy/Browser/wwwtax.cgi?id=1245035) | [bats](https://www.ncbi.nlm.nih.gov/Taxonomy/Browser/wwwtax.cgi?id=9397) | 150 | [2](https://blast.ncbi.nlm.nih.gov/Blast.cgi) |
| *...*[*Myonycteris brachycephala*](https://www.ncbi.nlm.nih.gov/Taxonomy/Browser/wwwtax.cgi?id=77237) | [bats](https://www.ncbi.nlm.nih.gov/Taxonomy/Browser/wwwtax.cgi?id=9397) | 150 | [1](https://blast.ncbi.nlm.nih.gov/Blast.cgi) |
| *...*[*Tayassu pecari*](https://www.ncbi.nlm.nih.gov/Taxonomy/Browser/wwwtax.cgi?id=30535) | [even-toed ungulates](https://www.ncbi.nlm.nih.gov/Taxonomy/Browser/wwwtax.cgi?id=91561) | 150 | [2](https://blast.ncbi.nlm.nih.gov/Blast.cgi) |
| *...Rousettus sp.* FCA-2016a | [bats](https://www.ncbi.nlm.nih.gov/Taxonomy/Browser/wwwtax.cgi?id=9397) | 150 | [1](https://blast.ncbi.nlm.nih.gov/Blast.cgi) |
| *...*[*Pteropus niger*](https://www.ncbi.nlm.nih.gov/Taxonomy/Browser/wwwtax.cgi?id=170225) | [bats](https://www.ncbi.nlm.nih.gov/Taxonomy/Browser/wwwtax.cgi?id=9397) | 150 | [2](https://blast.ncbi.nlm.nih.gov/Blast.cgi) |
| *...*[*Pteropus mahaganus*](https://www.ncbi.nlm.nih.gov/Taxonomy/Browser/wwwtax.cgi?id=1496129) | [bats](https://www.ncbi.nlm.nih.gov/Taxonomy/Browser/wwwtax.cgi?id=9397) | 150 | [1](https://blast.ncbi.nlm.nih.gov/Blast.cgi) |
| *...*[*Pteropus seychellensis comorensis*](https://www.ncbi.nlm.nih.gov/Taxonomy/Browser/wwwtax.cgi?id=589510) | [bats](https://www.ncbi.nlm.nih.gov/Taxonomy/Browser/wwwtax.cgi?id=9397) | 150 | [5](https://blast.ncbi.nlm.nih.gov/Blast.cgi) |
| *...*[*Pteropus seychellensis seychellensis*](https://www.ncbi.nlm.nih.gov/Taxonomy/Browser/wwwtax.cgi?id=589511) | [bats](https://www.ncbi.nlm.nih.gov/Taxonomy/Browser/wwwtax.cgi?id=9397) | 150 | [3](https://blast.ncbi.nlm.nih.gov/Blast.cgi) |
| *...*[*Pteropus pelagicus*](https://www.ncbi.nlm.nih.gov/Taxonomy/Browser/wwwtax.cgi?id=1495906) | [bats](https://www.ncbi.nlm.nih.gov/Taxonomy/Browser/wwwtax.cgi?id=9397) | 150 | [1](https://blast.ncbi.nlm.nih.gov/Blast.cgi) |
| *...*[*Pteropus scapulatus*](https://www.ncbi.nlm.nih.gov/Taxonomy/Browser/wwwtax.cgi?id=94117) | [bats](https://www.ncbi.nlm.nih.gov/Taxonomy/Browser/wwwtax.cgi?id=9397) | 150 | [5](https://blast.ncbi.nlm.nih.gov/Blast.cgi) |
| *...*[*Pteropus lylei*](https://www.ncbi.nlm.nih.gov/Taxonomy/Browser/wwwtax.cgi?id=166054) | [bats](https://www.ncbi.nlm.nih.gov/Taxonomy/Browser/wwwtax.cgi?id=9397) | 150 | [2](https://blast.ncbi.nlm.nih.gov/Blast.cgi) |
| *...*[*Pteropus pumilus*](https://www.ncbi.nlm.nih.gov/Taxonomy/Browser/wwwtax.cgi?id=161598) | [bats](https://www.ncbi.nlm.nih.gov/Taxonomy/Browser/wwwtax.cgi?id=9397) | 150 | [12](https://blast.ncbi.nlm.nih.gov/Blast.cgi) |
| *...*[*Madoqua saltiana*](https://www.ncbi.nlm.nih.gov/Taxonomy/Browser/wwwtax.cgi?id=1027994) | [even-toed ungulates](https://www.ncbi.nlm.nih.gov/Taxonomy/Browser/wwwtax.cgi?id=91561) | 150 | [3](https://blast.ncbi.nlm.nih.gov/Blast.cgi) |
| *...*[*Megaerops kusnotoi*](https://www.ncbi.nlm.nih.gov/Taxonomy/Browser/wwwtax.cgi?id=374419) | [bats](https://www.ncbi.nlm.nih.gov/Taxonomy/Browser/wwwtax.cgi?id=9397) | 150 | [1](https://blast.ncbi.nlm.nih.gov/Blast.cgi) |
| *...*[*Ptenochirus jagorii*](https://www.ncbi.nlm.nih.gov/Taxonomy/Browser/wwwtax.cgi?id=2933645) | [bats](https://www.ncbi.nlm.nih.gov/Taxonomy/Browser/wwwtax.cgi?id=9397) | 150 | [2](https://blast.ncbi.nlm.nih.gov/Blast.cgi) |
| *...*[*Cynopterus horsfieldii*](https://www.ncbi.nlm.nih.gov/Taxonomy/Browser/wwwtax.cgi?id=298109) | [bats](https://www.ncbi.nlm.nih.gov/Taxonomy/Browser/wwwtax.cgi?id=9397) | 150 | [5](https://blast.ncbi.nlm.nih.gov/Blast.cgi) |
| *...*[*Epomops dobsonii*](https://www.ncbi.nlm.nih.gov/Taxonomy/Browser/wwwtax.cgi?id=1812330) | [bats](https://www.ncbi.nlm.nih.gov/Taxonomy/Browser/wwwtax.cgi?id=9397) | 148 | [2](https://blast.ncbi.nlm.nih.gov/Blast.cgi) |
| *...Epomophorus sp.* MNHN ZM 2011-760 | [bats](https://www.ncbi.nlm.nih.gov/Taxonomy/Browser/wwwtax.cgi?id=9397) | 148 | [1](https://blast.ncbi.nlm.nih.gov/Blast.cgi) |
| *...*[*Epomophorus minor*](https://www.ncbi.nlm.nih.gov/Taxonomy/Browser/wwwtax.cgi?id=302407) | [bats](https://www.ncbi.nlm.nih.gov/Taxonomy/Browser/wwwtax.cgi?id=9397) | 148 | [4](https://blast.ncbi.nlm.nih.gov/Blast.cgi) |
| *...Epomophorus sp.* MNHN ZM 2011-726 | [bats](https://www.ncbi.nlm.nih.gov/Taxonomy/Browser/wwwtax.cgi?id=9397) | 148 | [1](https://blast.ncbi.nlm.nih.gov/Blast.cgi) |
| *...*[*Rucervus duvaucelii*](https://www.ncbi.nlm.nih.gov/Taxonomy/Browser/wwwtax.cgi?id=43328) | [even-toed ungulates](https://www.ncbi.nlm.nih.gov/Taxonomy/Browser/wwwtax.cgi?id=91561) | 148 | [8](https://blast.ncbi.nlm.nih.gov/Blast.cgi) |
| *...*[*Raphicerus melanotis*](https://www.ncbi.nlm.nih.gov/Taxonomy/Browser/wwwtax.cgi?id=66435) | [even-toed ungulates](https://www.ncbi.nlm.nih.gov/Taxonomy/Browser/wwwtax.cgi?id=91561) | 148 | [1](https://blast.ncbi.nlm.nih.gov/Blast.cgi) |
| *...*[*Ictonyx libycus*](https://www.ncbi.nlm.nih.gov/Taxonomy/Browser/wwwtax.cgi?id=470560) | [carnivores](https://www.ncbi.nlm.nih.gov/Taxonomy/Browser/wwwtax.cgi?id=33554) | 148 | [2](https://blast.ncbi.nlm.nih.gov/Blast.cgi) |
| *...*[*Pseudois nayaur szechuanensis*](https://www.ncbi.nlm.nih.gov/Taxonomy/Browser/wwwtax.cgi?id=1531488) | [even-toed ungulates](https://www.ncbi.nlm.nih.gov/Taxonomy/Browser/wwwtax.cgi?id=91561) | 148 | [1](https://blast.ncbi.nlm.nih.gov/Blast.cgi) |
| *...*[*Mirimiri acrodonta*](https://www.ncbi.nlm.nih.gov/Taxonomy/Browser/wwwtax.cgi?id=1496138) | [bats](https://www.ncbi.nlm.nih.gov/Taxonomy/Browser/wwwtax.cgi?id=9397) | 148 | [2](https://blast.ncbi.nlm.nih.gov/Blast.cgi) |
| *...*[*Rucervus duvaucelii duvaucelii*](https://www.ncbi.nlm.nih.gov/Taxonomy/Browser/wwwtax.cgi?id=2170375) | [even-toed ungulates](https://www.ncbi.nlm.nih.gov/Taxonomy/Browser/wwwtax.cgi?id=91561) | 148 | [2](https://blast.ncbi.nlm.nih.gov/Blast.cgi) |
| *...*[*Hemitragus jemlahicus*](https://www.ncbi.nlm.nih.gov/Taxonomy/Browser/wwwtax.cgi?id=37179) | [even-toed ungulates](https://www.ncbi.nlm.nih.gov/Taxonomy/Browser/wwwtax.cgi?id=91561) | 148 | [4](https://blast.ncbi.nlm.nih.gov/Blast.cgi) |
| *...*[*Dobsonia minor*](https://www.ncbi.nlm.nih.gov/Taxonomy/Browser/wwwtax.cgi?id=170215) | [bats](https://www.ncbi.nlm.nih.gov/Taxonomy/Browser/wwwtax.cgi?id=9397) | 148 | [1](https://blast.ncbi.nlm.nih.gov/Blast.cgi) |
| *...*[*Alionycteris paucidentata*](https://www.ncbi.nlm.nih.gov/Taxonomy/Browser/wwwtax.cgi?id=662937) | [bats](https://www.ncbi.nlm.nih.gov/Taxonomy/Browser/wwwtax.cgi?id=9397) | 148 | [2](https://blast.ncbi.nlm.nih.gov/Blast.cgi) |
| *...*[*Mustela nivalis*](https://www.ncbi.nlm.nih.gov/Taxonomy/Browser/wwwtax.cgi?id=36239) | [carnivores](https://www.ncbi.nlm.nih.gov/Taxonomy/Browser/wwwtax.cgi?id=33554) | 148 | [1](https://blast.ncbi.nlm.nih.gov/Blast.cgi) |
| *...*[*Kogia breviceps*](https://www.ncbi.nlm.nih.gov/Taxonomy/Browser/wwwtax.cgi?id=27615) | [whales & dolphins](https://www.ncbi.nlm.nih.gov/Taxonomy/Browser/wwwtax.cgi?id=9721) | 148 | [2](https://blast.ncbi.nlm.nih.gov/Blast.cgi) |
| *...*[*Dobsonia andersoni*](https://www.ncbi.nlm.nih.gov/Taxonomy/Browser/wwwtax.cgi?id=170213) | [bats](https://www.ncbi.nlm.nih.gov/Taxonomy/Browser/wwwtax.cgi?id=9397) | 148 | [1](https://blast.ncbi.nlm.nih.gov/Blast.cgi) |
| *...*[*Nyctimene cephalotes*](https://www.ncbi.nlm.nih.gov/Taxonomy/Browser/wwwtax.cgi?id=170209) | [bats](https://www.ncbi.nlm.nih.gov/Taxonomy/Browser/wwwtax.cgi?id=9397) | 147 | [3](https://blast.ncbi.nlm.nih.gov/Blast.cgi) |
| *...*[*Otocyon megalotis*](https://www.ncbi.nlm.nih.gov/Taxonomy/Browser/wwwtax.cgi?id=9624) | [carnivores](https://www.ncbi.nlm.nih.gov/Taxonomy/Browser/wwwtax.cgi?id=33554) | 147 | [3](https://blast.ncbi.nlm.nih.gov/Blast.cgi) |
| *...*[*Stenella attenuata*](https://www.ncbi.nlm.nih.gov/Taxonomy/Browser/wwwtax.cgi?id=9735) | [whales & dolphins](https://www.ncbi.nlm.nih.gov/Taxonomy/Browser/wwwtax.cgi?id=9721) | 147 | [1](https://blast.ncbi.nlm.nih.gov/Blast.cgi) |
| *...*[*Vulpes chama*](https://www.ncbi.nlm.nih.gov/Taxonomy/Browser/wwwtax.cgi?id=9626) | [carnivores](https://www.ncbi.nlm.nih.gov/Taxonomy/Browser/wwwtax.cgi?id=33554) | 147 | [2](https://blast.ncbi.nlm.nih.gov/Blast.cgi) |
| *...*[*Vulpes lagopus*](https://www.ncbi.nlm.nih.gov/Taxonomy/Browser/wwwtax.cgi?id=494514) | [carnivores](https://www.ncbi.nlm.nih.gov/Taxonomy/Browser/wwwtax.cgi?id=33554) | 147 | [6](https://blast.ncbi.nlm.nih.gov/Blast.cgi) |
| *...*[*Canis mesomelas*](https://www.ncbi.nlm.nih.gov/Taxonomy/Browser/wwwtax.cgi?id=68725) | [carnivores](https://www.ncbi.nlm.nih.gov/Taxonomy/Browser/wwwtax.cgi?id=33554) | 147 | [1](https://blast.ncbi.nlm.nih.gov/Blast.cgi) |
| *...*[*Canis lupus*](https://www.ncbi.nlm.nih.gov/Taxonomy/Browser/wwwtax.cgi?id=9612) | [carnivores](https://www.ncbi.nlm.nih.gov/Taxonomy/Browser/wwwtax.cgi?id=33554) | 147 | [352](https://blast.ncbi.nlm.nih.gov/Blast.cgi) |
| *...*[*Cephalophus silvicultor*](https://www.ncbi.nlm.nih.gov/Taxonomy/Browser/wwwtax.cgi?id=50347) | [even-toed ungulates](https://www.ncbi.nlm.nih.gov/Taxonomy/Browser/wwwtax.cgi?id=91561) | 147 | [2](https://blast.ncbi.nlm.nih.gov/Blast.cgi) |
| *...*[*Cephalophorus ogilbyi*](https://www.ncbi.nlm.nih.gov/Taxonomy/Browser/wwwtax.cgi?id=129228) | [even-toed ungulates](https://www.ncbi.nlm.nih.gov/Taxonomy/Browser/wwwtax.cgi?id=91561) | 147 | [2](https://blast.ncbi.nlm.nih.gov/Blast.cgi) |
| *...*[*Cephalophorus natalensis*](https://www.ncbi.nlm.nih.gov/Taxonomy/Browser/wwwtax.cgi?id=69299) | [even-toed ungulates](https://www.ncbi.nlm.nih.gov/Taxonomy/Browser/wwwtax.cgi?id=91561) | 147 | [2](https://blast.ncbi.nlm.nih.gov/Blast.cgi) |
| *...*[*Cephalophorus leucogaster*](https://www.ncbi.nlm.nih.gov/Taxonomy/Browser/wwwtax.cgi?id=70738) | [even-toed ungulates](https://www.ncbi.nlm.nih.gov/Taxonomy/Browser/wwwtax.cgi?id=91561) | 147 | [2](https://blast.ncbi.nlm.nih.gov/Blast.cgi) |
| *...*[*Cephalophus dorsalis*](https://www.ncbi.nlm.nih.gov/Taxonomy/Browser/wwwtax.cgi?id=97360) | [even-toed ungulates](https://www.ncbi.nlm.nih.gov/Taxonomy/Browser/wwwtax.cgi?id=91561) | 147 | [3](https://blast.ncbi.nlm.nih.gov/Blast.cgi) |
| *...*[*Cephalophorus callipygus*](https://www.ncbi.nlm.nih.gov/Taxonomy/Browser/wwwtax.cgi?id=129223) | [even-toed ungulates](https://www.ncbi.nlm.nih.gov/Taxonomy/Browser/wwwtax.cgi?id=91561) | 147 | [4](https://blast.ncbi.nlm.nih.gov/Blast.cgi) |
| *...*[*Nyctimene vizcaccia*](https://www.ncbi.nlm.nih.gov/Taxonomy/Browser/wwwtax.cgi?id=170211) | [bats](https://www.ncbi.nlm.nih.gov/Taxonomy/Browser/wwwtax.cgi?id=9397) | 147 | [1](https://blast.ncbi.nlm.nih.gov/Blast.cgi) |
| *...*[*Hyperoodon ampullatus*](https://www.ncbi.nlm.nih.gov/Taxonomy/Browser/wwwtax.cgi?id=48744) | [whales & dolphins](https://www.ncbi.nlm.nih.gov/Taxonomy/Browser/wwwtax.cgi?id=9721) | 145 | [139](https://blast.ncbi.nlm.nih.gov/Blast.cgi) |
| *...*[*Moschus moschiferus*](https://www.ncbi.nlm.nih.gov/Taxonomy/Browser/wwwtax.cgi?id=68415) | [even-toed ungulates](https://www.ncbi.nlm.nih.gov/Taxonomy/Browser/wwwtax.cgi?id=91561) | 145 | [4](https://blast.ncbi.nlm.nih.gov/Blast.cgi) |
| *...*[*Pteropus vampyrus*](https://www.ncbi.nlm.nih.gov/Taxonomy/Browser/wwwtax.cgi?id=132908) | [bats](https://www.ncbi.nlm.nih.gov/Taxonomy/Browser/wwwtax.cgi?id=9397) | 145 | [7](https://blast.ncbi.nlm.nih.gov/Blast.cgi) |
| *...*[*Pteropus woodfordi*](https://www.ncbi.nlm.nih.gov/Taxonomy/Browser/wwwtax.cgi?id=170232) | [bats](https://www.ncbi.nlm.nih.gov/Taxonomy/Browser/wwwtax.cgi?id=9397) | 145 | [1](https://blast.ncbi.nlm.nih.gov/Blast.cgi) |
| *...*[*Giraffa giraffa angolensis*](https://www.ncbi.nlm.nih.gov/Taxonomy/Browser/wwwtax.cgi?id=439323) | [even-toed ungulates](https://www.ncbi.nlm.nih.gov/Taxonomy/Browser/wwwtax.cgi?id=91561) | 145 | [7](https://blast.ncbi.nlm.nih.gov/Blast.cgi) |
| *...*[*Aepyceros melampus*](https://www.ncbi.nlm.nih.gov/Taxonomy/Browser/wwwtax.cgi?id=9897) | [even-toed ungulates](https://www.ncbi.nlm.nih.gov/Taxonomy/Browser/wwwtax.cgi?id=91561) | 145 | [3](https://blast.ncbi.nlm.nih.gov/Blast.cgi) |
| *...*[*Pteropus gilliardorum*](https://www.ncbi.nlm.nih.gov/Taxonomy/Browser/wwwtax.cgi?id=1496135) | [bats](https://www.ncbi.nlm.nih.gov/Taxonomy/Browser/wwwtax.cgi?id=9397) | 145 | [1](https://blast.ncbi.nlm.nih.gov/Blast.cgi) |
| *...*[*Pteropus aldabrensis*](https://www.ncbi.nlm.nih.gov/Taxonomy/Browser/wwwtax.cgi?id=589507) | [bats](https://www.ncbi.nlm.nih.gov/Taxonomy/Browser/wwwtax.cgi?id=9397) | 145 | [1](https://blast.ncbi.nlm.nih.gov/Blast.cgi) |
| *...*[*Pteropus rufus*](https://www.ncbi.nlm.nih.gov/Taxonomy/Browser/wwwtax.cgi?id=196297) | [bats](https://www.ncbi.nlm.nih.gov/Taxonomy/Browser/wwwtax.cgi?id=9397) | 145 | [1](https://blast.ncbi.nlm.nih.gov/Blast.cgi) |
| *...*[*Pteropus rodricensis*](https://www.ncbi.nlm.nih.gov/Taxonomy/Browser/wwwtax.cgi?id=77216) | [bats](https://www.ncbi.nlm.nih.gov/Taxonomy/Browser/wwwtax.cgi?id=9397) | 145 | [2](https://blast.ncbi.nlm.nih.gov/Blast.cgi) |
| *...*[*Pteropus livingstonii*](https://www.ncbi.nlm.nih.gov/Taxonomy/Browser/wwwtax.cgi?id=589508) | [bats](https://www.ncbi.nlm.nih.gov/Taxonomy/Browser/wwwtax.cgi?id=9397) | 145 | [1](https://blast.ncbi.nlm.nih.gov/Blast.cgi) |
| *...*[*Pteropus ornatus*](https://www.ncbi.nlm.nih.gov/Taxonomy/Browser/wwwtax.cgi?id=170227) | [bats](https://www.ncbi.nlm.nih.gov/Taxonomy/Browser/wwwtax.cgi?id=9397) | 145 | [1](https://blast.ncbi.nlm.nih.gov/Blast.cgi) |
| *...*[*Pteropus ocularis*](https://www.ncbi.nlm.nih.gov/Taxonomy/Browser/wwwtax.cgi?id=170226) | [bats](https://www.ncbi.nlm.nih.gov/Taxonomy/Browser/wwwtax.cgi?id=9397) | 145 | [1](https://blast.ncbi.nlm.nih.gov/Blast.cgi) |
| *...*[*Pteropus cognatus*](https://www.ncbi.nlm.nih.gov/Taxonomy/Browser/wwwtax.cgi?id=170222) | [bats](https://www.ncbi.nlm.nih.gov/Taxonomy/Browser/wwwtax.cgi?id=9397) | 145 | [2](https://blast.ncbi.nlm.nih.gov/Blast.cgi) |
| *...*[*Pteropus anetianus*](https://www.ncbi.nlm.nih.gov/Taxonomy/Browser/wwwtax.cgi?id=170220) | [bats](https://www.ncbi.nlm.nih.gov/Taxonomy/Browser/wwwtax.cgi?id=9397) | 145 | [1](https://blast.ncbi.nlm.nih.gov/Blast.cgi) |
| *...Lycalopex sp.* CAN003 | [carnivores](https://www.ncbi.nlm.nih.gov/Taxonomy/Browser/wwwtax.cgi?id=33554) | 143 | [1](https://blast.ncbi.nlm.nih.gov/Blast.cgi) |
| *...*[*Nanonycteris veldkampii*](https://www.ncbi.nlm.nih.gov/Taxonomy/Browser/wwwtax.cgi?id=498220) | [bats](https://www.ncbi.nlm.nih.gov/Taxonomy/Browser/wwwtax.cgi?id=9397) | 143 | [5](https://blast.ncbi.nlm.nih.gov/Blast.cgi) |
| *...*[*Epomophorus minimus*](https://www.ncbi.nlm.nih.gov/Taxonomy/Browser/wwwtax.cgi?id=1898388) | [bats](https://www.ncbi.nlm.nih.gov/Taxonomy/Browser/wwwtax.cgi?id=9397) | 143 | [2](https://blast.ncbi.nlm.nih.gov/Blast.cgi) |
| *...*[*Epomophorus labiatus*](https://www.ncbi.nlm.nih.gov/Taxonomy/Browser/wwwtax.cgi?id=903567) | [bats](https://www.ncbi.nlm.nih.gov/Taxonomy/Browser/wwwtax.cgi?id=9397) | 143 | [3](https://blast.ncbi.nlm.nih.gov/Blast.cgi) |
| *...*[*Epomophorus crypturus*](https://www.ncbi.nlm.nih.gov/Taxonomy/Browser/wwwtax.cgi?id=59450) | [bats](https://www.ncbi.nlm.nih.gov/Taxonomy/Browser/wwwtax.cgi?id=9397) | 143 | [3](https://blast.ncbi.nlm.nih.gov/Blast.cgi) |
| *...*[*Rucervus duvaucelii branderi*](https://www.ncbi.nlm.nih.gov/Taxonomy/Browser/wwwtax.cgi?id=1704528) | [even-toed ungulates](https://www.ncbi.nlm.nih.gov/Taxonomy/Browser/wwwtax.cgi?id=91561) | 143 | [6](https://blast.ncbi.nlm.nih.gov/Blast.cgi) |
| *...*[*Saiga tatarica*](https://www.ncbi.nlm.nih.gov/Taxonomy/Browser/wwwtax.cgi?id=34875) | [even-toed ungulates](https://www.ncbi.nlm.nih.gov/Taxonomy/Browser/wwwtax.cgi?id=91561) | 143 | [7](https://blast.ncbi.nlm.nih.gov/Blast.cgi) |
| *...*[*Rucervus duvaucelii ranjitsinhi*](https://www.ncbi.nlm.nih.gov/Taxonomy/Browser/wwwtax.cgi?id=2863190) | [even-toed ungulates](https://www.ncbi.nlm.nih.gov/Taxonomy/Browser/wwwtax.cgi?id=91561) | 143 | [2](https://blast.ncbi.nlm.nih.gov/Blast.cgi) |
| *...*[*Dama dama*](https://www.ncbi.nlm.nih.gov/Taxonomy/Browser/wwwtax.cgi?id=30532) | [even-toed ungulates](https://www.ncbi.nlm.nih.gov/Taxonomy/Browser/wwwtax.cgi?id=91561) | 143 | [5](https://blast.ncbi.nlm.nih.gov/Blast.cgi) |
| *...*[*Gazella arabica*](https://www.ncbi.nlm.nih.gov/Taxonomy/Browser/wwwtax.cgi?id=1229624) | [even-toed ungulates](https://www.ncbi.nlm.nih.gov/Taxonomy/Browser/wwwtax.cgi?id=91561) | 143 | [1](https://blast.ncbi.nlm.nih.gov/Blast.cgi) |
| *...*[*Neotragus batesi*](https://www.ncbi.nlm.nih.gov/Taxonomy/Browser/wwwtax.cgi?id=1088033) | [even-toed ungulates](https://www.ncbi.nlm.nih.gov/Taxonomy/Browser/wwwtax.cgi?id=91561) | 143 | [2](https://blast.ncbi.nlm.nih.gov/Blast.cgi) |
| *...*[*Nanger soemmerringii*](https://www.ncbi.nlm.nih.gov/Taxonomy/Browser/wwwtax.cgi?id=69306) | [even-toed ungulates](https://www.ncbi.nlm.nih.gov/Taxonomy/Browser/wwwtax.cgi?id=91561) | 143 | [2](https://blast.ncbi.nlm.nih.gov/Blast.cgi) |
| *...*[*Saiga tatarica mongolica*](https://www.ncbi.nlm.nih.gov/Taxonomy/Browser/wwwtax.cgi?id=1218238) | [even-toed ungulates](https://www.ncbi.nlm.nih.gov/Taxonomy/Browser/wwwtax.cgi?id=91561) | 143 | [1](https://blast.ncbi.nlm.nih.gov/Blast.cgi) |
| *...*[*Harpyionycteris whiteheadi*](https://www.ncbi.nlm.nih.gov/Taxonomy/Browser/wwwtax.cgi?id=378750) | [bats](https://www.ncbi.nlm.nih.gov/Taxonomy/Browser/wwwtax.cgi?id=9397) | 143 | [1](https://blast.ncbi.nlm.nih.gov/Blast.cgi) |
| *...*[*Pseudois nayaur*](https://www.ncbi.nlm.nih.gov/Taxonomy/Browser/wwwtax.cgi?id=59542) | [even-toed ungulates](https://www.ncbi.nlm.nih.gov/Taxonomy/Browser/wwwtax.cgi?id=91561) | 143 | [3](https://blast.ncbi.nlm.nih.gov/Blast.cgi) |
| *...*[*Antilope cervicapra*](https://www.ncbi.nlm.nih.gov/Taxonomy/Browser/wwwtax.cgi?id=59525) | [even-toed ungulates](https://www.ncbi.nlm.nih.gov/Taxonomy/Browser/wwwtax.cgi?id=91561) | 143 | [2](https://blast.ncbi.nlm.nih.gov/Blast.cgi) |
| *...*[*Mormoops blainvillei*](https://www.ncbi.nlm.nih.gov/Taxonomy/Browser/wwwtax.cgi?id=118852) | [bats](https://www.ncbi.nlm.nih.gov/Taxonomy/Browser/wwwtax.cgi?id=9397) | 143 | [1](https://blast.ncbi.nlm.nih.gov/Blast.cgi) |
| *...*[*Pseudonovibos spiralis*](https://www.ncbi.nlm.nih.gov/Taxonomy/Browser/wwwtax.cgi?id=77135) | [even-toed ungulates](https://www.ncbi.nlm.nih.gov/Taxonomy/Browser/wwwtax.cgi?id=91561) | 143 | [1](https://blast.ncbi.nlm.nih.gov/Blast.cgi) |
| *...*[*Moschus leucogaster*](https://www.ncbi.nlm.nih.gov/Taxonomy/Browser/wwwtax.cgi?id=68414) | [even-toed ungulates](https://www.ncbi.nlm.nih.gov/Taxonomy/Browser/wwwtax.cgi?id=91561) | 143 | [1](https://blast.ncbi.nlm.nih.gov/Blast.cgi) |
| *...*[*Hypsignathus monstrosus*](https://www.ncbi.nlm.nih.gov/Taxonomy/Browser/wwwtax.cgi?id=448084) | [bats](https://www.ncbi.nlm.nih.gov/Taxonomy/Browser/wwwtax.cgi?id=9397) | 141 | [3](https://blast.ncbi.nlm.nih.gov/Blast.cgi) |
| *...*[*Canis lupus hodophilax*](https://www.ncbi.nlm.nih.gov/Taxonomy/Browser/wwwtax.cgi?id=188536) | [carnivores](https://www.ncbi.nlm.nih.gov/Taxonomy/Browser/wwwtax.cgi?id=33554) | 141 | [7](https://blast.ncbi.nlm.nih.gov/Blast.cgi) |
| *...*[*Canis lupus orion*](https://www.ncbi.nlm.nih.gov/Taxonomy/Browser/wwwtax.cgi?id=2605939) | [carnivores](https://www.ncbi.nlm.nih.gov/Taxonomy/Browser/wwwtax.cgi?id=33554) | 141 | [1](https://blast.ncbi.nlm.nih.gov/Blast.cgi) |
| *...*[*Canis lupus lupus*](https://www.ncbi.nlm.nih.gov/Taxonomy/Browser/wwwtax.cgi?id=443256) | [carnivores](https://www.ncbi.nlm.nih.gov/Taxonomy/Browser/wwwtax.cgi?id=33554) | 141 | [1](https://blast.ncbi.nlm.nih.gov/Blast.cgi) |
| *...*[*Canis lupus dingo*](https://www.ncbi.nlm.nih.gov/Taxonomy/Browser/wwwtax.cgi?id=286419) | [carnivores](https://www.ncbi.nlm.nih.gov/Taxonomy/Browser/wwwtax.cgi?id=33554) | 141 | [5](https://blast.ncbi.nlm.nih.gov/Blast.cgi) |
| *...*[*Canis simensis*](https://www.ncbi.nlm.nih.gov/Taxonomy/Browser/wwwtax.cgi?id=32534) | [carnivores](https://www.ncbi.nlm.nih.gov/Taxonomy/Browser/wwwtax.cgi?id=33554) | 141 | [4](https://blast.ncbi.nlm.nih.gov/Blast.cgi) |
| *...*[*Canis latrans*](https://www.ncbi.nlm.nih.gov/Taxonomy/Browser/wwwtax.cgi?id=9614) | [carnivores](https://www.ncbi.nlm.nih.gov/Taxonomy/Browser/wwwtax.cgi?id=33554) | 141 | [42](https://blast.ncbi.nlm.nih.gov/Blast.cgi) |
| *...*[*Vulpes ferrilata*](https://www.ncbi.nlm.nih.gov/Taxonomy/Browser/wwwtax.cgi?id=561074) | [carnivores](https://www.ncbi.nlm.nih.gov/Taxonomy/Browser/wwwtax.cgi?id=33554) | 141 | [2](https://blast.ncbi.nlm.nih.gov/Blast.cgi) |
| *...*[*Canis aureus*](https://www.ncbi.nlm.nih.gov/Taxonomy/Browser/wwwtax.cgi?id=68724) | [carnivores](https://www.ncbi.nlm.nih.gov/Taxonomy/Browser/wwwtax.cgi?id=33554) | 141 | [2](https://blast.ncbi.nlm.nih.gov/Blast.cgi) |
| *...*[*Canis aureus cruesemanni*](https://www.ncbi.nlm.nih.gov/Taxonomy/Browser/wwwtax.cgi?id=2957507) | [carnivores](https://www.ncbi.nlm.nih.gov/Taxonomy/Browser/wwwtax.cgi?id=33554) | 141 | [1](https://blast.ncbi.nlm.nih.gov/Blast.cgi) |
| *...*[*Canis rufus*](https://www.ncbi.nlm.nih.gov/Taxonomy/Browser/wwwtax.cgi?id=45781) | [carnivores](https://www.ncbi.nlm.nih.gov/Taxonomy/Browser/wwwtax.cgi?id=33554) | 141 | [9](https://blast.ncbi.nlm.nih.gov/Blast.cgi) |
| *...*[*Canis lupus signatus*](https://www.ncbi.nlm.nih.gov/Taxonomy/Browser/wwwtax.cgi?id=425934) | [carnivores](https://www.ncbi.nlm.nih.gov/Taxonomy/Browser/wwwtax.cgi?id=33554) | 141 | [7](https://blast.ncbi.nlm.nih.gov/Blast.cgi) |
| *...*[*Canis lupus pallipes*](https://www.ncbi.nlm.nih.gov/Taxonomy/Browser/wwwtax.cgi?id=246882) | [carnivores](https://www.ncbi.nlm.nih.gov/Taxonomy/Browser/wwwtax.cgi?id=33554) | 141 | [7](https://blast.ncbi.nlm.nih.gov/Blast.cgi) |
| *...*[*Vulpes corsac*](https://www.ncbi.nlm.nih.gov/Taxonomy/Browser/wwwtax.cgi?id=9629) | [carnivores](https://www.ncbi.nlm.nih.gov/Taxonomy/Browser/wwwtax.cgi?id=33554) | 141 | [2](https://blast.ncbi.nlm.nih.gov/Blast.cgi) |
| *...*[*Canis lupus chanco*](https://www.ncbi.nlm.nih.gov/Taxonomy/Browser/wwwtax.cgi?id=246881) | [carnivores](https://www.ncbi.nlm.nih.gov/Taxonomy/Browser/wwwtax.cgi?id=33554) | 141 | [2](https://blast.ncbi.nlm.nih.gov/Blast.cgi) |
| *...*[*Canis lupus lycaon*](https://www.ncbi.nlm.nih.gov/Taxonomy/Browser/wwwtax.cgi?id=228401) | [carnivores](https://www.ncbi.nlm.nih.gov/Taxonomy/Browser/wwwtax.cgi?id=33554) | 141 | [3](https://blast.ncbi.nlm.nih.gov/Blast.cgi) |
| *...*[*Canis sp. Russia/33,500*](https://www.ncbi.nlm.nih.gov/Taxonomy/Browser/wwwtax.cgi?id=1419712) | [carnivores](https://www.ncbi.nlm.nih.gov/Taxonomy/Browser/wwwtax.cgi?id=33554) | 141 | [1](https://blast.ncbi.nlm.nih.gov/Blast.cgi) |
| *...*[*Canis sp. Belgium/36,000*](https://www.ncbi.nlm.nih.gov/Taxonomy/Browser/wwwtax.cgi?id=1419257) | [carnivores](https://www.ncbi.nlm.nih.gov/Taxonomy/Browser/wwwtax.cgi?id=33554) | 141 | [1](https://blast.ncbi.nlm.nih.gov/Blast.cgi) |
| *...*[*Canis lupus laniger*](https://www.ncbi.nlm.nih.gov/Taxonomy/Browser/wwwtax.cgi?id=554455) | [carnivores](https://www.ncbi.nlm.nih.gov/Taxonomy/Browser/wwwtax.cgi?id=33554) | 141 | [1](https://blast.ncbi.nlm.nih.gov/Blast.cgi) |
| *...*[*Canis lupus baileyi*](https://www.ncbi.nlm.nih.gov/Taxonomy/Browser/wwwtax.cgi?id=143281) | [carnivores](https://www.ncbi.nlm.nih.gov/Taxonomy/Browser/wwwtax.cgi?id=33554) | 141 | [7](https://blast.ncbi.nlm.nih.gov/Blast.cgi) |
| *...*[*Canis lupus campestris*](https://www.ncbi.nlm.nih.gov/Taxonomy/Browser/wwwtax.cgi?id=1341016) | [carnivores](https://www.ncbi.nlm.nih.gov/Taxonomy/Browser/wwwtax.cgi?id=33554) | 141 | [1](https://blast.ncbi.nlm.nih.gov/Blast.cgi) |
| *...*[*Canis lupus desertorum*](https://www.ncbi.nlm.nih.gov/Taxonomy/Browser/wwwtax.cgi?id=1295334) | [carnivores](https://www.ncbi.nlm.nih.gov/Taxonomy/Browser/wwwtax.cgi?id=33554) | 141 | [1](https://blast.ncbi.nlm.nih.gov/Blast.cgi) |
| *...*[*Sylvicapra grimmia*](https://www.ncbi.nlm.nih.gov/Taxonomy/Browser/wwwtax.cgi?id=119562) | [even-toed ungulates](https://www.ncbi.nlm.nih.gov/Taxonomy/Browser/wwwtax.cgi?id=91561) | 141 | [2](https://blast.ncbi.nlm.nih.gov/Blast.cgi) |
| *...*[*Litocranius walleri*](https://www.ncbi.nlm.nih.gov/Taxonomy/Browser/wwwtax.cgi?id=69311) | [even-toed ungulates](https://www.ncbi.nlm.nih.gov/Taxonomy/Browser/wwwtax.cgi?id=91561) | 141 | [2](https://blast.ncbi.nlm.nih.gov/Blast.cgi) |
| *...*[*Cephalophorus rufilatus*](https://www.ncbi.nlm.nih.gov/Taxonomy/Browser/wwwtax.cgi?id=129230) | [even-toed ungulates](https://www.ncbi.nlm.nih.gov/Taxonomy/Browser/wwwtax.cgi?id=91561) | 141 | [2](https://blast.ncbi.nlm.nih.gov/Blast.cgi) |
| *...*[*Cephalophorus nigrifrons*](https://www.ncbi.nlm.nih.gov/Taxonomy/Browser/wwwtax.cgi?id=129227) | [even-toed ungulates](https://www.ncbi.nlm.nih.gov/Taxonomy/Browser/wwwtax.cgi?id=91561) | 141 | [2](https://blast.ncbi.nlm.nih.gov/Blast.cgi) |
| *..*[*Propithecus deckenii*](https://www.ncbi.nlm.nih.gov/Taxonomy/Browser/wwwtax.cgi?id=475618) | [primates](https://www.ncbi.nlm.nih.gov/Taxonomy/Browser/wwwtax.cgi?id=9443) | 172 | [7](https://blast.ncbi.nlm.nih.gov/Blast.cgi) |
| *..*[*Apodemus semotus*](https://www.ncbi.nlm.nih.gov/Taxonomy/Browser/wwwtax.cgi?id=105299) | [rodents](https://www.ncbi.nlm.nih.gov/Taxonomy/Browser/wwwtax.cgi?id=9989) | 169 | [2](https://blast.ncbi.nlm.nih.gov/Blast.cgi) |
| *..*[*Procolobus verus*](https://www.ncbi.nlm.nih.gov/Taxonomy/Browser/wwwtax.cgi?id=373033) | [primates](https://www.ncbi.nlm.nih.gov/Taxonomy/Browser/wwwtax.cgi?id=9443) | 169 | [2](https://blast.ncbi.nlm.nih.gov/Blast.cgi) |
| *..*[*Apodemus draco*](https://www.ncbi.nlm.nih.gov/Taxonomy/Browser/wwwtax.cgi?id=129247) | [rodents](https://www.ncbi.nlm.nih.gov/Taxonomy/Browser/wwwtax.cgi?id=9989) | 163 | [3](https://blast.ncbi.nlm.nih.gov/Blast.cgi) |
| *..*[*Propithecus deckenii coronatus*](https://www.ncbi.nlm.nih.gov/Taxonomy/Browser/wwwtax.cgi?id=475619) | [primates](https://www.ncbi.nlm.nih.gov/Taxonomy/Browser/wwwtax.cgi?id=9443) | 163 | [4](https://blast.ncbi.nlm.nih.gov/Blast.cgi) |
| *..*[*Propithecus verreauxi*](https://www.ncbi.nlm.nih.gov/Taxonomy/Browser/wwwtax.cgi?id=34825) | [primates](https://www.ncbi.nlm.nih.gov/Taxonomy/Browser/wwwtax.cgi?id=9443) | 163 | [43](https://blast.ncbi.nlm.nih.gov/Blast.cgi) |
| *..*[*Neotoma mexicana*](https://www.ncbi.nlm.nih.gov/Taxonomy/Browser/wwwtax.cgi?id=105149) | [rodents](https://www.ncbi.nlm.nih.gov/Taxonomy/Browser/wwwtax.cgi?id=9989) | 161 | [5](https://blast.ncbi.nlm.nih.gov/Blast.cgi) |
| *..*[*Romerolagus diazi*](https://www.ncbi.nlm.nih.gov/Taxonomy/Browser/wwwtax.cgi?id=48089) | [rabbits & hares](https://www.ncbi.nlm.nih.gov/Taxonomy/Browser/wwwtax.cgi?id=9975) | 161 | [5](https://blast.ncbi.nlm.nih.gov/Blast.cgi) |
| *..*[*Grammomys macmillani*](https://www.ncbi.nlm.nih.gov/Taxonomy/Browser/wwwtax.cgi?id=248794) | [rodents](https://www.ncbi.nlm.nih.gov/Taxonomy/Browser/wwwtax.cgi?id=9989) | 161 | [1](https://blast.ncbi.nlm.nih.gov/Blast.cgi) |
| *..*[*Neotoma stephensi*](https://www.ncbi.nlm.nih.gov/Taxonomy/Browser/wwwtax.cgi?id=164611) | [rodents](https://www.ncbi.nlm.nih.gov/Taxonomy/Browser/wwwtax.cgi?id=9989) | 161 | [1](https://blast.ncbi.nlm.nih.gov/Blast.cgi) |
| *..*[*Neotoma isthmica*](https://www.ncbi.nlm.nih.gov/Taxonomy/Browser/wwwtax.cgi?id=316116) | [rodents](https://www.ncbi.nlm.nih.gov/Taxonomy/Browser/wwwtax.cgi?id=9989) | 161 | [2](https://blast.ncbi.nlm.nih.gov/Blast.cgi) |
| *..*[*Dactylomys boliviensis*](https://www.ncbi.nlm.nih.gov/Taxonomy/Browser/wwwtax.cgi?id=30618) | [rodents](https://www.ncbi.nlm.nih.gov/Taxonomy/Browser/wwwtax.cgi?id=9989) | 159 | [1](https://blast.ncbi.nlm.nih.gov/Blast.cgi) |
| *..*[*Hystrix indica*](https://www.ncbi.nlm.nih.gov/Taxonomy/Browser/wwwtax.cgi?id=192883) | [rodents](https://www.ncbi.nlm.nih.gov/Taxonomy/Browser/wwwtax.cgi?id=9989) | 158 | [4](https://blast.ncbi.nlm.nih.gov/Blast.cgi) |
| *..*[*Hystrix brachyura*](https://www.ncbi.nlm.nih.gov/Taxonomy/Browser/wwwtax.cgi?id=143286) | [rodents](https://www.ncbi.nlm.nih.gov/Taxonomy/Browser/wwwtax.cgi?id=9989) | 158 | [1](https://blast.ncbi.nlm.nih.gov/Blast.cgi) |
| *..*[*Apodemus agrarius*](https://www.ncbi.nlm.nih.gov/Taxonomy/Browser/wwwtax.cgi?id=39030) | [rodents](https://www.ncbi.nlm.nih.gov/Taxonomy/Browser/wwwtax.cgi?id=9989) | 158 | [76](https://blast.ncbi.nlm.nih.gov/Blast.cgi) |
| *..*[*Pipanacoctomys aureus*](https://www.ncbi.nlm.nih.gov/Taxonomy/Browser/wwwtax.cgi?id=227730) | [rodents](https://www.ncbi.nlm.nih.gov/Taxonomy/Browser/wwwtax.cgi?id=9989) | 158 | [2](https://blast.ncbi.nlm.nih.gov/Blast.cgi) |
| *..*[*Grammomys surdaster*](https://www.ncbi.nlm.nih.gov/Taxonomy/Browser/wwwtax.cgi?id=491861) | [rodents](https://www.ncbi.nlm.nih.gov/Taxonomy/Browser/wwwtax.cgi?id=9989) | 158 | [4](https://blast.ncbi.nlm.nih.gov/Blast.cgi) |
| *..*[*Aethomys nyikae*](https://www.ncbi.nlm.nih.gov/Taxonomy/Browser/wwwtax.cgi?id=1890048) | [rodents](https://www.ncbi.nlm.nih.gov/Taxonomy/Browser/wwwtax.cgi?id=9989) | 158 | [1](https://blast.ncbi.nlm.nih.gov/Blast.cgi) |
| *..*[*Apodemus chevrieri*](https://www.ncbi.nlm.nih.gov/Taxonomy/Browser/wwwtax.cgi?id=129246) | [rodents](https://www.ncbi.nlm.nih.gov/Taxonomy/Browser/wwwtax.cgi?id=9989) | 158 | [2](https://blast.ncbi.nlm.nih.gov/Blast.cgi) |
| *..*[*Apodemus chejuensis*](https://www.ncbi.nlm.nih.gov/Taxonomy/Browser/wwwtax.cgi?id=754351) | [rodents](https://www.ncbi.nlm.nih.gov/Taxonomy/Browser/wwwtax.cgi?id=9989) | 158 | [2](https://blast.ncbi.nlm.nih.gov/Blast.cgi) |
| *..*[*Neotoma cinerea*](https://www.ncbi.nlm.nih.gov/Taxonomy/Browser/wwwtax.cgi?id=105147) | [rodents](https://www.ncbi.nlm.nih.gov/Taxonomy/Browser/wwwtax.cgi?id=9989) | 158 | [2](https://blast.ncbi.nlm.nih.gov/Blast.cgi) |
| *..*[*Georychus capensis*](https://www.ncbi.nlm.nih.gov/Taxonomy/Browser/wwwtax.cgi?id=10177) | [rodents](https://www.ncbi.nlm.nih.gov/Taxonomy/Browser/wwwtax.cgi?id=9989) | 158 | [3](https://blast.ncbi.nlm.nih.gov/Blast.cgi) |
| *..*[*Abrocoma cinerea*](https://www.ncbi.nlm.nih.gov/Taxonomy/Browser/wwwtax.cgi?id=126064) | [rodents](https://www.ncbi.nlm.nih.gov/Taxonomy/Browser/wwwtax.cgi?id=9989) | 158 | [1](https://blast.ncbi.nlm.nih.gov/Blast.cgi) |
| *..*[*Trinomys eliasi*](https://www.ncbi.nlm.nih.gov/Taxonomy/Browser/wwwtax.cgi?id=42828) | [rodents](https://www.ncbi.nlm.nih.gov/Taxonomy/Browser/wwwtax.cgi?id=9989) | 158 | [1](https://blast.ncbi.nlm.nih.gov/Blast.cgi) |
| *..*[*Apodemus latronum*](https://www.ncbi.nlm.nih.gov/Taxonomy/Browser/wwwtax.cgi?id=214933) | [rodents](https://www.ncbi.nlm.nih.gov/Taxonomy/Browser/wwwtax.cgi?id=9989) | 156 | [2](https://blast.ncbi.nlm.nih.gov/Blast.cgi) |
| *..*[*Micaelamys namaquensis*](https://www.ncbi.nlm.nih.gov/Taxonomy/Browser/wwwtax.cgi?id=472707) | [rodents](https://www.ncbi.nlm.nih.gov/Taxonomy/Browser/wwwtax.cgi?id=9989) | 156 | [4](https://blast.ncbi.nlm.nih.gov/Blast.cgi) |
| *..*[*Aethomys chrysophilus*](https://www.ncbi.nlm.nih.gov/Taxonomy/Browser/wwwtax.cgi?id=121561) | [rodents](https://www.ncbi.nlm.nih.gov/Taxonomy/Browser/wwwtax.cgi?id=9989) | 156 | [1](https://blast.ncbi.nlm.nih.gov/Blast.cgi) |
| *..*[*Colobus guereza*](https://www.ncbi.nlm.nih.gov/Taxonomy/Browser/wwwtax.cgi?id=33548) | [primates](https://www.ncbi.nlm.nih.gov/Taxonomy/Browser/wwwtax.cgi?id=9443) | 156 | [3](https://blast.ncbi.nlm.nih.gov/Blast.cgi) |
| *..*[*Neotoma lepida*](https://www.ncbi.nlm.nih.gov/Taxonomy/Browser/wwwtax.cgi?id=56216) | [rodents](https://www.ncbi.nlm.nih.gov/Taxonomy/Browser/wwwtax.cgi?id=9989) | 156 | [10](https://blast.ncbi.nlm.nih.gov/Blast.cgi) |
| *..*[*Colobus satanas*](https://www.ncbi.nlm.nih.gov/Taxonomy/Browser/wwwtax.cgi?id=517012) | [primates](https://www.ncbi.nlm.nih.gov/Taxonomy/Browser/wwwtax.cgi?id=9443) | 154 | [2](https://blast.ncbi.nlm.nih.gov/Blast.cgi) |
| *..*[*Piliocolobus badius*](https://www.ncbi.nlm.nih.gov/Taxonomy/Browser/wwwtax.cgi?id=164648) | [primates](https://www.ncbi.nlm.nih.gov/Taxonomy/Browser/wwwtax.cgi?id=9443) | 154 | [3](https://blast.ncbi.nlm.nih.gov/Blast.cgi) |
| *..*[*Isothrix sinnamariensis*](https://www.ncbi.nlm.nih.gov/Taxonomy/Browser/wwwtax.cgi?id=297005) | [rodents](https://www.ncbi.nlm.nih.gov/Taxonomy/Browser/wwwtax.cgi?id=9989) | 152 | [2](https://blast.ncbi.nlm.nih.gov/Blast.cgi) |
| *..*[*Phyllomys pattoni*](https://www.ncbi.nlm.nih.gov/Taxonomy/Browser/wwwtax.cgi?id=466164) | [rodents](https://www.ncbi.nlm.nih.gov/Taxonomy/Browser/wwwtax.cgi?id=9989) | 152 | [1](https://blast.ncbi.nlm.nih.gov/Blast.cgi) |
| *..*[*Propithecus edwardsi*](https://www.ncbi.nlm.nih.gov/Taxonomy/Browser/wwwtax.cgi?id=543559) | [primates](https://www.ncbi.nlm.nih.gov/Taxonomy/Browser/wwwtax.cgi?id=9443) | 152 | [31](https://blast.ncbi.nlm.nih.gov/Blast.cgi) |
| *..*[*Avahi laniger*](https://www.ncbi.nlm.nih.gov/Taxonomy/Browser/wwwtax.cgi?id=122246) | [primates](https://www.ncbi.nlm.nih.gov/Taxonomy/Browser/wwwtax.cgi?id=9443) | 152 | [3](https://blast.ncbi.nlm.nih.gov/Blast.cgi) |
| *..*[*Isothrix pagurus*](https://www.ncbi.nlm.nih.gov/Taxonomy/Browser/wwwtax.cgi?id=30625) | [rodents](https://www.ncbi.nlm.nih.gov/Taxonomy/Browser/wwwtax.cgi?id=9989) | 152 | [1](https://blast.ncbi.nlm.nih.gov/Blast.cgi) |
| *..*[*Grammomys selousi*](https://www.ncbi.nlm.nih.gov/Taxonomy/Browser/wwwtax.cgi?id=2781600) | [rodents](https://www.ncbi.nlm.nih.gov/Taxonomy/Browser/wwwtax.cgi?id=9989) | 152 | [2](https://blast.ncbi.nlm.nih.gov/Blast.cgi) |
| *..*[*Grammomys dolichurus*](https://www.ncbi.nlm.nih.gov/Taxonomy/Browser/wwwtax.cgi?id=491754) | [rodents](https://www.ncbi.nlm.nih.gov/Taxonomy/Browser/wwwtax.cgi?id=9989) | 152 | [2](https://blast.ncbi.nlm.nih.gov/Blast.cgi) |
| *..*[*Hydrochoerus hydrochaeris*](https://www.ncbi.nlm.nih.gov/Taxonomy/Browser/wwwtax.cgi?id=10149) | [rodents](https://www.ncbi.nlm.nih.gov/Taxonomy/Browser/wwwtax.cgi?id=9989) | 152 | [5](https://blast.ncbi.nlm.nih.gov/Blast.cgi) |
| *..*[*Dinomys branickii*](https://www.ncbi.nlm.nih.gov/Taxonomy/Browser/wwwtax.cgi?id=108858) | [rodents](https://www.ncbi.nlm.nih.gov/Taxonomy/Browser/wwwtax.cgi?id=9989) | 152 | [3](https://blast.ncbi.nlm.nih.gov/Blast.cgi) |
| *..*[*Apodemus peninsulae*](https://www.ncbi.nlm.nih.gov/Taxonomy/Browser/wwwtax.cgi?id=105297) | [rodents](https://www.ncbi.nlm.nih.gov/Taxonomy/Browser/wwwtax.cgi?id=9989) | 152 | [4](https://blast.ncbi.nlm.nih.gov/Blast.cgi) |
| *..*[*Chiropodomys gliroides*](https://www.ncbi.nlm.nih.gov/Taxonomy/Browser/wwwtax.cgi?id=491874) | [rodents](https://www.ncbi.nlm.nih.gov/Taxonomy/Browser/wwwtax.cgi?id=9989) | 150 | [2](https://blast.ncbi.nlm.nih.gov/Blast.cgi) |
| *..*[*Hylomyscus stella*](https://www.ncbi.nlm.nih.gov/Taxonomy/Browser/wwwtax.cgi?id=41265) | [rodents](https://www.ncbi.nlm.nih.gov/Taxonomy/Browser/wwwtax.cgi?id=9989) | 150 | [14](https://blast.ncbi.nlm.nih.gov/Blast.cgi) |
| *..*[*Kannabateomys amblyonyx*](https://www.ncbi.nlm.nih.gov/Taxonomy/Browser/wwwtax.cgi?id=176503) | [rodents](https://www.ncbi.nlm.nih.gov/Taxonomy/Browser/wwwtax.cgi?id=9989) | 150 | [2](https://blast.ncbi.nlm.nih.gov/Blast.cgi) |
| *..*[*Neotoma fuscipes*](https://www.ncbi.nlm.nih.gov/Taxonomy/Browser/wwwtax.cgi?id=105199) | [rodents](https://www.ncbi.nlm.nih.gov/Taxonomy/Browser/wwwtax.cgi?id=9989) | 150 | [7](https://blast.ncbi.nlm.nih.gov/Blast.cgi) |
| *..*[*Trinomys setosus*](https://www.ncbi.nlm.nih.gov/Taxonomy/Browser/wwwtax.cgi?id=43323) | [rodents](https://www.ncbi.nlm.nih.gov/Taxonomy/Browser/wwwtax.cgi?id=9989) | 150 | [2](https://blast.ncbi.nlm.nih.gov/Blast.cgi) |
| *..*[*Apodemus alpicola*](https://www.ncbi.nlm.nih.gov/Taxonomy/Browser/wwwtax.cgi?id=100381) | [rodents](https://www.ncbi.nlm.nih.gov/Taxonomy/Browser/wwwtax.cgi?id=9989) | 150 | [3](https://blast.ncbi.nlm.nih.gov/Blast.cgi) |
| *..*[*Colomys lumumbai*](https://www.ncbi.nlm.nih.gov/Taxonomy/Browser/wwwtax.cgi?id=2866652) | [rodents](https://www.ncbi.nlm.nih.gov/Taxonomy/Browser/wwwtax.cgi?id=9989) | 150 | [1](https://blast.ncbi.nlm.nih.gov/Blast.cgi) |
| *..*[*Rattus rattus*](https://www.ncbi.nlm.nih.gov/Taxonomy/Browser/wwwtax.cgi?id=10117) | [rodents](https://www.ncbi.nlm.nih.gov/Taxonomy/Browser/wwwtax.cgi?id=9989) | 150 | [42](https://blast.ncbi.nlm.nih.gov/Blast.cgi) |
| *..*[*Nannospalax nehringi*](https://www.ncbi.nlm.nih.gov/Taxonomy/Browser/wwwtax.cgi?id=614052) | [rodents](https://www.ncbi.nlm.nih.gov/Taxonomy/Browser/wwwtax.cgi?id=9989) | 150 | [1](https://blast.ncbi.nlm.nih.gov/Blast.cgi) |
| *..*[*Rattus fuscipes*](https://www.ncbi.nlm.nih.gov/Taxonomy/Browser/wwwtax.cgi?id=10119) | [rodents](https://www.ncbi.nlm.nih.gov/Taxonomy/Browser/wwwtax.cgi?id=9989) | 150 | [2](https://blast.ncbi.nlm.nih.gov/Blast.cgi) |
| *..*[*Neotoma picta*](https://www.ncbi.nlm.nih.gov/Taxonomy/Browser/wwwtax.cgi?id=316115) | [rodents](https://www.ncbi.nlm.nih.gov/Taxonomy/Browser/wwwtax.cgi?id=9989) | 150 | [1](https://blast.ncbi.nlm.nih.gov/Blast.cgi) |
| *..*[*Neotoma leucodon*](https://www.ncbi.nlm.nih.gov/Taxonomy/Browser/wwwtax.cgi?id=268937) | [rodents](https://www.ncbi.nlm.nih.gov/Taxonomy/Browser/wwwtax.cgi?id=9989) | 150 | [1](https://blast.ncbi.nlm.nih.gov/Blast.cgi) |
| *..*[*Neotoma goldmani*](https://www.ncbi.nlm.nih.gov/Taxonomy/Browser/wwwtax.cgi?id=105148) | [rodents](https://www.ncbi.nlm.nih.gov/Taxonomy/Browser/wwwtax.cgi?id=9989) | 150 | [1](https://blast.ncbi.nlm.nih.gov/Blast.cgi) |
| *..*[*Trinomys setosus setosus*](https://www.ncbi.nlm.nih.gov/Taxonomy/Browser/wwwtax.cgi?id=109338) | [rodents](https://www.ncbi.nlm.nih.gov/Taxonomy/Browser/wwwtax.cgi?id=9989) | 150 | [1](https://blast.ncbi.nlm.nih.gov/Blast.cgi) |
| *..*[*Trinomys setosus elegans*](https://www.ncbi.nlm.nih.gov/Taxonomy/Browser/wwwtax.cgi?id=176496) | [rodents](https://www.ncbi.nlm.nih.gov/Taxonomy/Browser/wwwtax.cgi?id=9989) | 150 | [1](https://blast.ncbi.nlm.nih.gov/Blast.cgi) |
| *..*[*Trinomys setosus denigratus*](https://www.ncbi.nlm.nih.gov/Taxonomy/Browser/wwwtax.cgi?id=109337) | [rodents](https://www.ncbi.nlm.nih.gov/Taxonomy/Browser/wwwtax.cgi?id=9989) | 150 | [1](https://blast.ncbi.nlm.nih.gov/Blast.cgi) |
| *..*[*Uranomys ruddi*](https://www.ncbi.nlm.nih.gov/Taxonomy/Browser/wwwtax.cgi?id=41272) | [rodents](https://www.ncbi.nlm.nih.gov/Taxonomy/Browser/wwwtax.cgi?id=9989) | 150 | [1](https://blast.ncbi.nlm.nih.gov/Blast.cgi) |
| *..*[*Leopoldamys edwardsi*](https://www.ncbi.nlm.nih.gov/Taxonomy/Browser/wwwtax.cgi?id=83756) | [rodents](https://www.ncbi.nlm.nih.gov/Taxonomy/Browser/wwwtax.cgi?id=9989) | 150 | [3](https://blast.ncbi.nlm.nih.gov/Blast.cgi) |
| *..*[*Niviventer cremoriventer*](https://www.ncbi.nlm.nih.gov/Taxonomy/Browser/wwwtax.cgi?id=69083) | [rodents](https://www.ncbi.nlm.nih.gov/Taxonomy/Browser/wwwtax.cgi?id=9989) | 148 | [3](https://blast.ncbi.nlm.nih.gov/Blast.cgi) |
| *..*[*Leopoldamys sabanus*](https://www.ncbi.nlm.nih.gov/Taxonomy/Browser/wwwtax.cgi?id=69073) | [rodents](https://www.ncbi.nlm.nih.gov/Taxonomy/Browser/wwwtax.cgi?id=9989) | 148 | [3](https://blast.ncbi.nlm.nih.gov/Blast.cgi) |
| *..*[*Trinomys albispinus*](https://www.ncbi.nlm.nih.gov/Taxonomy/Browser/wwwtax.cgi?id=109335) | [rodents](https://www.ncbi.nlm.nih.gov/Taxonomy/Browser/wwwtax.cgi?id=9989) | 148 | [1](https://blast.ncbi.nlm.nih.gov/Blast.cgi) |
| *..*[*Ptilocercus lowii*](https://www.ncbi.nlm.nih.gov/Taxonomy/Browser/wwwtax.cgi?id=320338) | [placentals](https://www.ncbi.nlm.nih.gov/Taxonomy/Browser/wwwtax.cgi?id=9347) | 148 | [2](https://blast.ncbi.nlm.nih.gov/Blast.cgi) |
| *..*[*Fukomys mechowii*](https://www.ncbi.nlm.nih.gov/Taxonomy/Browser/wwwtax.cgi?id=423607) | [rodents](https://www.ncbi.nlm.nih.gov/Taxonomy/Browser/wwwtax.cgi?id=9989) | 148 | [5](https://blast.ncbi.nlm.nih.gov/Blast.cgi) |
| *..*[*Fukomys sp. Kasama*](https://www.ncbi.nlm.nih.gov/Taxonomy/Browser/wwwtax.cgi?id=260988) | [rodents](https://www.ncbi.nlm.nih.gov/Taxonomy/Browser/wwwtax.cgi?id=9989) | 148 | [1](https://blast.ncbi.nlm.nih.gov/Blast.cgi) |
| *..*[*Hybomys trivirgatus*](https://www.ncbi.nlm.nih.gov/Taxonomy/Browser/wwwtax.cgi?id=2107264) | [rodents](https://www.ncbi.nlm.nih.gov/Taxonomy/Browser/wwwtax.cgi?id=9989) | 147 | [2](https://blast.ncbi.nlm.nih.gov/Blast.cgi) |
| *..*[*Trichys fasciculata*](https://www.ncbi.nlm.nih.gov/Taxonomy/Browser/wwwtax.cgi?id=73865) | [rodents](https://www.ncbi.nlm.nih.gov/Taxonomy/Browser/wwwtax.cgi?id=9989) | 147 | [2](https://blast.ncbi.nlm.nih.gov/Blast.cgi) |
| *..*[*Cuscomys ashaninka*](https://www.ncbi.nlm.nih.gov/Taxonomy/Browser/wwwtax.cgi?id=1567517) | [rodents](https://www.ncbi.nlm.nih.gov/Taxonomy/Browser/wwwtax.cgi?id=9989) | 147 | [1](https://blast.ncbi.nlm.nih.gov/Blast.cgi) |
| *..*[*Hapalomys delacouri*](https://www.ncbi.nlm.nih.gov/Taxonomy/Browser/wwwtax.cgi?id=1156415) | [rodents](https://www.ncbi.nlm.nih.gov/Taxonomy/Browser/wwwtax.cgi?id=9989) | 147 | [1](https://blast.ncbi.nlm.nih.gov/Blast.cgi) |
| *..*[*Isothrix orinoci*](https://www.ncbi.nlm.nih.gov/Taxonomy/Browser/wwwtax.cgi?id=490303) | [rodents](https://www.ncbi.nlm.nih.gov/Taxonomy/Browser/wwwtax.cgi?id=9989) | 147 | [1](https://blast.ncbi.nlm.nih.gov/Blast.cgi) |
| *..*[*Golunda ellioti*](https://www.ncbi.nlm.nih.gov/Taxonomy/Browser/wwwtax.cgi?id=121585) | [rodents](https://www.ncbi.nlm.nih.gov/Taxonomy/Browser/wwwtax.cgi?id=9989) | 147 | [2](https://blast.ncbi.nlm.nih.gov/Blast.cgi) |
| *..Hybomys sp. n.* VVB-2020 | [rodents](https://www.ncbi.nlm.nih.gov/Taxonomy/Browser/wwwtax.cgi?id=9989) | 147 | [1](https://blast.ncbi.nlm.nih.gov/Blast.cgi) |
| *..*[*Semnopithecus entellus*](https://www.ncbi.nlm.nih.gov/Taxonomy/Browser/wwwtax.cgi?id=88029) | [primates](https://www.ncbi.nlm.nih.gov/Taxonomy/Browser/wwwtax.cgi?id=9443) | 147 | [3](https://blast.ncbi.nlm.nih.gov/Blast.cgi) |
| *..*[*Avahi occidentalis*](https://www.ncbi.nlm.nih.gov/Taxonomy/Browser/wwwtax.cgi?id=132108) | [primates](https://www.ncbi.nlm.nih.gov/Taxonomy/Browser/wwwtax.cgi?id=9443) | 147 | [2](https://blast.ncbi.nlm.nih.gov/Blast.cgi) |
| *..*[*Propithecus verreauxi verreauxi*](https://www.ncbi.nlm.nih.gov/Taxonomy/Browser/wwwtax.cgi?id=122229) | [primates](https://www.ncbi.nlm.nih.gov/Taxonomy/Browser/wwwtax.cgi?id=9443) | 147 | [1](https://blast.ncbi.nlm.nih.gov/Blast.cgi) |
| *..*[*Calomyscus mystax*](https://www.ncbi.nlm.nih.gov/Taxonomy/Browser/wwwtax.cgi?id=109677) | [rodents](https://www.ncbi.nlm.nih.gov/Taxonomy/Browser/wwwtax.cgi?id=9989) | 147 | [2](https://blast.ncbi.nlm.nih.gov/Blast.cgi) |
| *..*[*Dolichotis salinicola*](https://www.ncbi.nlm.nih.gov/Taxonomy/Browser/wwwtax.cgi?id=181543) | [rodents](https://www.ncbi.nlm.nih.gov/Taxonomy/Browser/wwwtax.cgi?id=9989) | 147 | [2](https://blast.ncbi.nlm.nih.gov/Blast.cgi) |
| *..*[*Trinomys paratus*](https://www.ncbi.nlm.nih.gov/Taxonomy/Browser/wwwtax.cgi?id=42827) | [rodents](https://www.ncbi.nlm.nih.gov/Taxonomy/Browser/wwwtax.cgi?id=9989) | 147 | [1](https://blast.ncbi.nlm.nih.gov/Blast.cgi) |
| *..*[*Mus baoulei*](https://www.ncbi.nlm.nih.gov/Taxonomy/Browser/wwwtax.cgi?id=544437) | [rodents](https://www.ncbi.nlm.nih.gov/Taxonomy/Browser/wwwtax.cgi?id=9989) | 145 | [2](https://blast.ncbi.nlm.nih.gov/Blast.cgi) |
| *..*[*Otomys irroratus*](https://www.ncbi.nlm.nih.gov/Taxonomy/Browser/wwwtax.cgi?id=121570) | [rodents](https://www.ncbi.nlm.nih.gov/Taxonomy/Browser/wwwtax.cgi?id=9989) | 145 | [3](https://blast.ncbi.nlm.nih.gov/Blast.cgi) |
| *..*[*Rattus tanezumi*](https://www.ncbi.nlm.nih.gov/Taxonomy/Browser/wwwtax.cgi?id=35732) | [rodents](https://www.ncbi.nlm.nih.gov/Taxonomy/Browser/wwwtax.cgi?id=9989) | 145 | [6](https://blast.ncbi.nlm.nih.gov/Blast.cgi) |
| *..*[*Rattus andamanensis*](https://www.ncbi.nlm.nih.gov/Taxonomy/Browser/wwwtax.cgi?id=69077) | [rodents](https://www.ncbi.nlm.nih.gov/Taxonomy/Browser/wwwtax.cgi?id=9989) | 145 | [2](https://blast.ncbi.nlm.nih.gov/Blast.cgi) |
| *..*[*Melanomys caliginosus*](https://www.ncbi.nlm.nih.gov/Taxonomy/Browser/wwwtax.cgi?id=218793) | [rodents](https://www.ncbi.nlm.nih.gov/Taxonomy/Browser/wwwtax.cgi?id=9989) | 145 | [1](https://blast.ncbi.nlm.nih.gov/Blast.cgi) |
| *..*[*Hylomyscus walterverheyeni*](https://www.ncbi.nlm.nih.gov/Taxonomy/Browser/wwwtax.cgi?id=397097) | [rodents](https://www.ncbi.nlm.nih.gov/Taxonomy/Browser/wwwtax.cgi?id=9989) | 145 | [1](https://blast.ncbi.nlm.nih.gov/Blast.cgi) |
| *..*[*Hylomyscus simus*](https://www.ncbi.nlm.nih.gov/Taxonomy/Browser/wwwtax.cgi?id=337584) | [rodents](https://www.ncbi.nlm.nih.gov/Taxonomy/Browser/wwwtax.cgi?id=9989) | 145 | [3](https://blast.ncbi.nlm.nih.gov/Blast.cgi) |
| *..*[*Hylomyscus arcimontensis*](https://www.ncbi.nlm.nih.gov/Taxonomy/Browser/wwwtax.cgi?id=1427028) | [rodents](https://www.ncbi.nlm.nih.gov/Taxonomy/Browser/wwwtax.cgi?id=9989) | 145 | [1](https://blast.ncbi.nlm.nih.gov/Blast.cgi) |
| *..*[*Hylomyscus anselli*](https://www.ncbi.nlm.nih.gov/Taxonomy/Browser/wwwtax.cgi?id=1254423) | [rodents](https://www.ncbi.nlm.nih.gov/Taxonomy/Browser/wwwtax.cgi?id=9989) | 145 | [3](https://blast.ncbi.nlm.nih.gov/Blast.cgi) |
| *..*[*Hybomys univittatus*](https://www.ncbi.nlm.nih.gov/Taxonomy/Browser/wwwtax.cgi?id=71162) | [rodents](https://www.ncbi.nlm.nih.gov/Taxonomy/Browser/wwwtax.cgi?id=9989) | 145 | [3](https://blast.ncbi.nlm.nih.gov/Blast.cgi) |
| *..*[*Sundamys infraluteus*](https://www.ncbi.nlm.nih.gov/Taxonomy/Browser/wwwtax.cgi?id=1936103) | [rodents](https://www.ncbi.nlm.nih.gov/Taxonomy/Browser/wwwtax.cgi?id=9989) | 145 | [2](https://blast.ncbi.nlm.nih.gov/Blast.cgi) |
| *..*[*Taeromys dominator*](https://www.ncbi.nlm.nih.gov/Taxonomy/Browser/wwwtax.cgi?id=3049457) | [rodents](https://www.ncbi.nlm.nih.gov/Taxonomy/Browser/wwwtax.cgi?id=9989) | 145 | [2](https://blast.ncbi.nlm.nih.gov/Blast.cgi) |
| *..*[*Rattus nitidus*](https://www.ncbi.nlm.nih.gov/Taxonomy/Browser/wwwtax.cgi?id=508668) | [rodents](https://www.ncbi.nlm.nih.gov/Taxonomy/Browser/wwwtax.cgi?id=9989) | 145 | [2](https://blast.ncbi.nlm.nih.gov/Blast.cgi) |
| *..*[*Niviventer fulvescens*](https://www.ncbi.nlm.nih.gov/Taxonomy/Browser/wwwtax.cgi?id=83774) | [rodents](https://www.ncbi.nlm.nih.gov/Taxonomy/Browser/wwwtax.cgi?id=9989) | 145 | [3](https://blast.ncbi.nlm.nih.gov/Blast.cgi) |
| *..*[*Arvicola amphibius*](https://www.ncbi.nlm.nih.gov/Taxonomy/Browser/wwwtax.cgi?id=1047088) | [rodents](https://www.ncbi.nlm.nih.gov/Taxonomy/Browser/wwwtax.cgi?id=9989) | 145 | [1](https://blast.ncbi.nlm.nih.gov/Blast.cgi) |
| *..*[*Praomys morio*](https://www.ncbi.nlm.nih.gov/Taxonomy/Browser/wwwtax.cgi?id=1116255) | [rodents](https://www.ncbi.nlm.nih.gov/Taxonomy/Browser/wwwtax.cgi?id=9989) | 145 | [2](https://blast.ncbi.nlm.nih.gov/Blast.cgi) |
| *..*[*Praomys minor*](https://www.ncbi.nlm.nih.gov/Taxonomy/Browser/wwwtax.cgi?id=666515) | [rodents](https://www.ncbi.nlm.nih.gov/Taxonomy/Browser/wwwtax.cgi?id=9989) | 145 | [2](https://blast.ncbi.nlm.nih.gov/Blast.cgi) |
| *..*[*Atherurus africanus*](https://www.ncbi.nlm.nih.gov/Taxonomy/Browser/wwwtax.cgi?id=192881) | [rodents](https://www.ncbi.nlm.nih.gov/Taxonomy/Browser/wwwtax.cgi?id=9989) | 145 | [2](https://blast.ncbi.nlm.nih.gov/Blast.cgi) |
| *..*[*Brachylagus idahoensis*](https://www.ncbi.nlm.nih.gov/Taxonomy/Browser/wwwtax.cgi?id=48083) | [rabbits & hares](https://www.ncbi.nlm.nih.gov/Taxonomy/Browser/wwwtax.cgi?id=9975) | 145 | [2](https://blast.ncbi.nlm.nih.gov/Blast.cgi) |
| *..*[*Neotoma melanura*](https://www.ncbi.nlm.nih.gov/Taxonomy/Browser/wwwtax.cgi?id=913574) | [rodents](https://www.ncbi.nlm.nih.gov/Taxonomy/Browser/wwwtax.cgi?id=9989) | 145 | [1](https://blast.ncbi.nlm.nih.gov/Blast.cgi) |
| *..*[*Rattus niobe*](https://www.ncbi.nlm.nih.gov/Taxonomy/Browser/wwwtax.cgi?id=472762) | [rodents](https://www.ncbi.nlm.nih.gov/Taxonomy/Browser/wwwtax.cgi?id=9989) | 145 | [2](https://blast.ncbi.nlm.nih.gov/Blast.cgi) |
| *..*[*Bandicota bengalensis*](https://www.ncbi.nlm.nih.gov/Taxonomy/Browser/wwwtax.cgi?id=69079) | [rodents](https://www.ncbi.nlm.nih.gov/Taxonomy/Browser/wwwtax.cgi?id=9989) | 145 | [2](https://blast.ncbi.nlm.nih.gov/Blast.cgi) |
| *..*[*Praomys delectorum*](https://www.ncbi.nlm.nih.gov/Taxonomy/Browser/wwwtax.cgi?id=119565) | [rodents](https://www.ncbi.nlm.nih.gov/Taxonomy/Browser/wwwtax.cgi?id=9989) | 145 | [2](https://blast.ncbi.nlm.nih.gov/Blast.cgi) |
| *..*[*Grammomys poensis*](https://www.ncbi.nlm.nih.gov/Taxonomy/Browser/wwwtax.cgi?id=498361) | [rodents](https://www.ncbi.nlm.nih.gov/Taxonomy/Browser/wwwtax.cgi?id=9989) | 145 | [1](https://blast.ncbi.nlm.nih.gov/Blast.cgi) |
| *..*[*Thamnomys kempi*](https://www.ncbi.nlm.nih.gov/Taxonomy/Browser/wwwtax.cgi?id=1890067) | [rodents](https://www.ncbi.nlm.nih.gov/Taxonomy/Browser/wwwtax.cgi?id=9989) | 145 | [2](https://blast.ncbi.nlm.nih.gov/Blast.cgi) |
| *..*[*Otomys sp. Mount Kenya*](https://www.ncbi.nlm.nih.gov/Taxonomy/Browser/wwwtax.cgi?id=2781603) | [rodents](https://www.ncbi.nlm.nih.gov/Taxonomy/Browser/wwwtax.cgi?id=9989) | 145 | [1](https://blast.ncbi.nlm.nih.gov/Blast.cgi) |
| *..*[*Stenocephalemys sokolovi*](https://www.ncbi.nlm.nih.gov/Taxonomy/Browser/wwwtax.cgi?id=2776655) | [rodents](https://www.ncbi.nlm.nih.gov/Taxonomy/Browser/wwwtax.cgi?id=9989) | 145 | [2](https://blast.ncbi.nlm.nih.gov/Blast.cgi) |
| *..*[*Stenocephalemys griseicauda*](https://www.ncbi.nlm.nih.gov/Taxonomy/Browser/wwwtax.cgi?id=89457) | [rodents](https://www.ncbi.nlm.nih.gov/Taxonomy/Browser/wwwtax.cgi?id=9989) | 145 | [4](https://blast.ncbi.nlm.nih.gov/Blast.cgi) |
| *..*[*Stenocephalemys albipes*](https://www.ncbi.nlm.nih.gov/Taxonomy/Browser/wwwtax.cgi?id=485440) | [rodents](https://www.ncbi.nlm.nih.gov/Taxonomy/Browser/wwwtax.cgi?id=9989) | 145 | [1](https://blast.ncbi.nlm.nih.gov/Blast.cgi) |
| *..*[*Nannospalax nehringi xanthodon*](https://www.ncbi.nlm.nih.gov/Taxonomy/Browser/wwwtax.cgi?id=1026976) | [rodents](https://www.ncbi.nlm.nih.gov/Taxonomy/Browser/wwwtax.cgi?id=9989) | 145 | [1](https://blast.ncbi.nlm.nih.gov/Blast.cgi) |
| *..*[*Rattus leucopus*](https://www.ncbi.nlm.nih.gov/Taxonomy/Browser/wwwtax.cgi?id=10115) | [rodents](https://www.ncbi.nlm.nih.gov/Taxonomy/Browser/wwwtax.cgi?id=9989) | 145 | [3](https://blast.ncbi.nlm.nih.gov/Blast.cgi) |
| *..*[*Rattus praetor*](https://www.ncbi.nlm.nih.gov/Taxonomy/Browser/wwwtax.cgi?id=349711) | [rodents](https://www.ncbi.nlm.nih.gov/Taxonomy/Browser/wwwtax.cgi?id=9989) | 145 | [2](https://blast.ncbi.nlm.nih.gov/Blast.cgi) |
| *..*[*Neotoma floridana*](https://www.ncbi.nlm.nih.gov/Taxonomy/Browser/wwwtax.cgi?id=42409) | [rodents](https://www.ncbi.nlm.nih.gov/Taxonomy/Browser/wwwtax.cgi?id=9989) | 145 | [4](https://blast.ncbi.nlm.nih.gov/Blast.cgi) |
| *..*[*Neotoma micropus*](https://www.ncbi.nlm.nih.gov/Taxonomy/Browser/wwwtax.cgi?id=105150) | [rodents](https://www.ncbi.nlm.nih.gov/Taxonomy/Browser/wwwtax.cgi?id=9989) | 145 | [5](https://blast.ncbi.nlm.nih.gov/Blast.cgi) |
| *..*[*Neotoma macrotis*](https://www.ncbi.nlm.nih.gov/Taxonomy/Browser/wwwtax.cgi?id=349162) | [rodents](https://www.ncbi.nlm.nih.gov/Taxonomy/Browser/wwwtax.cgi?id=9989) | 145 | [4](https://blast.ncbi.nlm.nih.gov/Blast.cgi) |
| *..*[*Desmodilliscus braueri*](https://www.ncbi.nlm.nih.gov/Taxonomy/Browser/wwwtax.cgi?id=298892) | [rodents](https://www.ncbi.nlm.nih.gov/Taxonomy/Browser/wwwtax.cgi?id=9989) | 145 | [1](https://blast.ncbi.nlm.nih.gov/Blast.cgi) |
| *..*[*Mus indutus*](https://www.ncbi.nlm.nih.gov/Taxonomy/Browser/wwwtax.cgi?id=273921) | [rodents](https://www.ncbi.nlm.nih.gov/Taxonomy/Browser/wwwtax.cgi?id=9989) | 145 | [1](https://blast.ncbi.nlm.nih.gov/Blast.cgi) |
| *..*[*Mus haussa*](https://www.ncbi.nlm.nih.gov/Taxonomy/Browser/wwwtax.cgi?id=273922) | [rodents](https://www.ncbi.nlm.nih.gov/Taxonomy/Browser/wwwtax.cgi?id=9989) | 145 | [1](https://blast.ncbi.nlm.nih.gov/Blast.cgi) |
| *..*[*Mus mattheyi*](https://www.ncbi.nlm.nih.gov/Taxonomy/Browser/wwwtax.cgi?id=41270) | [rodents](https://www.ncbi.nlm.nih.gov/Taxonomy/Browser/wwwtax.cgi?id=9989) | 145 | [1](https://blast.ncbi.nlm.nih.gov/Blast.cgi) |
| *..*[*Oryctolagus cuniculus*](https://www.ncbi.nlm.nih.gov/Taxonomy/Browser/wwwtax.cgi?id=9986) | [rabbits & hares](https://www.ncbi.nlm.nih.gov/Taxonomy/Browser/wwwtax.cgi?id=9975) | 143 | [12](https://blast.ncbi.nlm.nih.gov/Blast.cgi) |
| *..*[*Proechimys kulinae*](https://www.ncbi.nlm.nih.gov/Taxonomy/Browser/wwwtax.cgi?id=2033317) | [rodents](https://www.ncbi.nlm.nih.gov/Taxonomy/Browser/wwwtax.cgi?id=9989) | 143 | [2](https://blast.ncbi.nlm.nih.gov/Blast.cgi) |
| *..*[*Trachypithecus vetulus*](https://www.ncbi.nlm.nih.gov/Taxonomy/Browser/wwwtax.cgi?id=54137) | [primates](https://www.ncbi.nlm.nih.gov/Taxonomy/Browser/wwwtax.cgi?id=9443) | 143 | [1](https://blast.ncbi.nlm.nih.gov/Blast.cgi) |
| *..*[*Chlorocebus tantalus*](https://www.ncbi.nlm.nih.gov/Taxonomy/Browser/wwwtax.cgi?id=60712) | [primates](https://www.ncbi.nlm.nih.gov/Taxonomy/Browser/wwwtax.cgi?id=9443) | 143 | [1](https://blast.ncbi.nlm.nih.gov/Blast.cgi) |
| *..*[*Chlorocebus djamdjamensis*](https://www.ncbi.nlm.nih.gov/Taxonomy/Browser/wwwtax.cgi?id=1284215) | [primates](https://www.ncbi.nlm.nih.gov/Taxonomy/Browser/wwwtax.cgi?id=9443) | 143 | [2](https://blast.ncbi.nlm.nih.gov/Blast.cgi) |
| *..*[*Chlorocebus cynosuros*](https://www.ncbi.nlm.nih.gov/Taxonomy/Browser/wwwtax.cgi?id=460675) | [primates](https://www.ncbi.nlm.nih.gov/Taxonomy/Browser/wwwtax.cgi?id=9443) | 143 | [3](https://blast.ncbi.nlm.nih.gov/Blast.cgi) |
| *..*[*Neacomys paracou*](https://www.ncbi.nlm.nih.gov/Taxonomy/Browser/wwwtax.cgi?id=461404) | [rodents](https://www.ncbi.nlm.nih.gov/Taxonomy/Browser/wwwtax.cgi?id=9989) | 143 | [3](https://blast.ncbi.nlm.nih.gov/Blast.cgi) |
| *..*[*Anomalurus beecrofti*](https://www.ncbi.nlm.nih.gov/Taxonomy/Browser/wwwtax.cgi?id=1082180) | [rodents](https://www.ncbi.nlm.nih.gov/Taxonomy/Browser/wwwtax.cgi?id=9989) | 143 | [1](https://blast.ncbi.nlm.nih.gov/Blast.cgi) |
| *..*[*Propithecus tattersalli*](https://www.ncbi.nlm.nih.gov/Taxonomy/Browser/wwwtax.cgi?id=30601) | [primates](https://www.ncbi.nlm.nih.gov/Taxonomy/Browser/wwwtax.cgi?id=9443) | 143 | [19](https://blast.ncbi.nlm.nih.gov/Blast.cgi) |
| *..*[*Propithecus coquereli*](https://www.ncbi.nlm.nih.gov/Taxonomy/Browser/wwwtax.cgi?id=379532) | [primates](https://www.ncbi.nlm.nih.gov/Taxonomy/Browser/wwwtax.cgi?id=9443) | 143 | [12](https://blast.ncbi.nlm.nih.gov/Blast.cgi) |
| *..*[*Semnopithecus schistaceus*](https://www.ncbi.nlm.nih.gov/Taxonomy/Browser/wwwtax.cgi?id=2804203) | [primates](https://www.ncbi.nlm.nih.gov/Taxonomy/Browser/wwwtax.cgi?id=9443) | 143 | [2](https://blast.ncbi.nlm.nih.gov/Blast.cgi) |
| *..*[*Fukomys darlingi*](https://www.ncbi.nlm.nih.gov/Taxonomy/Browser/wwwtax.cgi?id=63616) | [rodents](https://www.ncbi.nlm.nih.gov/Taxonomy/Browser/wwwtax.cgi?id=9989) | 143 | [2](https://blast.ncbi.nlm.nih.gov/Blast.cgi) |
| *..*[*Fukomys damarensis*](https://www.ncbi.nlm.nih.gov/Taxonomy/Browser/wwwtax.cgi?id=885580) | [rodents](https://www.ncbi.nlm.nih.gov/Taxonomy/Browser/wwwtax.cgi?id=9989) | 143 | [2](https://blast.ncbi.nlm.nih.gov/Blast.cgi) |
| *..*[*Octomys mimax*](https://www.ncbi.nlm.nih.gov/Taxonomy/Browser/wwwtax.cgi?id=135583) | [rodents](https://www.ncbi.nlm.nih.gov/Taxonomy/Browser/wwwtax.cgi?id=9989) | 143 | [2](https://blast.ncbi.nlm.nih.gov/Blast.cgi) |
| *..*[*Propithecus diadema*](https://www.ncbi.nlm.nih.gov/Taxonomy/Browser/wwwtax.cgi?id=83281) | [primates](https://www.ncbi.nlm.nih.gov/Taxonomy/Browser/wwwtax.cgi?id=9443) | 143 | [1](https://blast.ncbi.nlm.nih.gov/Blast.cgi) |
| *..*[*Malacomys edwardsi*](https://www.ncbi.nlm.nih.gov/Taxonomy/Browser/wwwtax.cgi?id=333388) | [rodents](https://www.ncbi.nlm.nih.gov/Taxonomy/Browser/wwwtax.cgi?id=9989) | 141 | [1](https://blast.ncbi.nlm.nih.gov/Blast.cgi) |
| *..*[*Hylomyscus heinrichorum*](https://www.ncbi.nlm.nih.gov/Taxonomy/Browser/wwwtax.cgi?id=2695852) | [rodents](https://www.ncbi.nlm.nih.gov/Taxonomy/Browser/wwwtax.cgi?id=9989) | 141 | [4](https://blast.ncbi.nlm.nih.gov/Blast.cgi) |
| *..*[*Oligoryzomys stramineus*](https://www.ncbi.nlm.nih.gov/Taxonomy/Browser/wwwtax.cgi?id=218826) | [rodents](https://www.ncbi.nlm.nih.gov/Taxonomy/Browser/wwwtax.cgi?id=9989) | 141 | [2](https://blast.ncbi.nlm.nih.gov/Blast.cgi) |
| *..*[*Myocastor coypus*](https://www.ncbi.nlm.nih.gov/Taxonomy/Browser/wwwtax.cgi?id=10157) | [rodents](https://www.ncbi.nlm.nih.gov/Taxonomy/Browser/wwwtax.cgi?id=9989) | 141 | [3](https://blast.ncbi.nlm.nih.gov/Blast.cgi) |
| *..*[*Sundamys muelleri*](https://www.ncbi.nlm.nih.gov/Taxonomy/Browser/wwwtax.cgi?id=83761) | [rodents](https://www.ncbi.nlm.nih.gov/Taxonomy/Browser/wwwtax.cgi?id=9989) | 141 | [1](https://blast.ncbi.nlm.nih.gov/Blast.cgi) |
| *..*[*Bunomys penitus*](https://www.ncbi.nlm.nih.gov/Taxonomy/Browser/wwwtax.cgi?id=1330375) | [rodents](https://www.ncbi.nlm.nih.gov/Taxonomy/Browser/wwwtax.cgi?id=9989) | 141 | [2](https://blast.ncbi.nlm.nih.gov/Blast.cgi) |
| *..*[*Macaca mulatta*](https://www.ncbi.nlm.nih.gov/Taxonomy/Browser/wwwtax.cgi?id=9544) | [primates](https://www.ncbi.nlm.nih.gov/Taxonomy/Browser/wwwtax.cgi?id=9443) | 141 | [26](https://blast.ncbi.nlm.nih.gov/Blast.cgi) |
| *..*[*Euchoreutes naso*](https://www.ncbi.nlm.nih.gov/Taxonomy/Browser/wwwtax.cgi?id=980894) | [rodents](https://www.ncbi.nlm.nih.gov/Taxonomy/Browser/wwwtax.cgi?id=9989) | 141 | [2](https://blast.ncbi.nlm.nih.gov/Blast.cgi) |
| *..Myomyscus sp.* ETH0898 | [rodents](https://www.ncbi.nlm.nih.gov/Taxonomy/Browser/wwwtax.cgi?id=9989) | 141 | [1](https://blast.ncbi.nlm.nih.gov/Blast.cgi) |
| *..*[*Cercopithecus nictitans martini*](https://www.ncbi.nlm.nih.gov/Taxonomy/Browser/wwwtax.cgi?id=1137494) | [primates](https://www.ncbi.nlm.nih.gov/Taxonomy/Browser/wwwtax.cgi?id=9443) | 141 | [1](https://blast.ncbi.nlm.nih.gov/Blast.cgi) |
| *..*[*Rattus norvegicus*](https://www.ncbi.nlm.nih.gov/Taxonomy/Browser/wwwtax.cgi?id=10116) | [rodents](https://www.ncbi.nlm.nih.gov/Taxonomy/Browser/wwwtax.cgi?id=9989) | 141 | [1](https://blast.ncbi.nlm.nih.gov/Blast.cgi) |
| *.*[*Hemicentetes nigriceps*](https://www.ncbi.nlm.nih.gov/Taxonomy/Browser/wwwtax.cgi?id=1432851) | [placentals](https://www.ncbi.nlm.nih.gov/Taxonomy/Browser/wwwtax.cgi?id=9347) | 158 | [1](https://blast.ncbi.nlm.nih.gov/Blast.cgi) |
| *.*[*Orycteropus afer*](https://www.ncbi.nlm.nih.gov/Taxonomy/Browser/wwwtax.cgi?id=9818) | [placentals](https://www.ncbi.nlm.nih.gov/Taxonomy/Browser/wwwtax.cgi?id=9347) | 154 | [4](https://blast.ncbi.nlm.nih.gov/Blast.cgi) |
| *.*[*Eremitalpa granti*](https://www.ncbi.nlm.nih.gov/Taxonomy/Browser/wwwtax.cgi?id=481707) | [placentals](https://www.ncbi.nlm.nih.gov/Taxonomy/Browser/wwwtax.cgi?id=9347) | 143 | [1](https://blast.ncbi.nlm.nih.gov/Blast.cgi) |
| *.*[*Chrysospalax villosus*](https://www.ncbi.nlm.nih.gov/Taxonomy/Browser/wwwtax.cgi?id=745250) | [placentals](https://www.ncbi.nlm.nih.gov/Taxonomy/Browser/wwwtax.cgi?id=9347) | 143 | [1](https://blast.ncbi.nlm.nih.gov/Blast.cgi) |
| *.*[*Hemicentetes semispinosus*](https://www.ncbi.nlm.nih.gov/Taxonomy/Browser/wwwtax.cgi?id=319813) | [placentals](https://www.ncbi.nlm.nih.gov/Taxonomy/Browser/wwwtax.cgi?id=9347) | 143 | [1](https://blast.ncbi.nlm.nih.gov/Blast.cgi) |
| 12sDNA used Target sequence (141 bp): TGGGAAGAAATGGGCTACATTTTCTATGACATAGAATAATTTAACGAAAACCTTCATGAAACCTGAAGGCTAAAGGAGGATTTAGTAGTAAATTAAGAATAGAGTGCTTAATTGAATTAGGCCATGAAGCACGCACACACC (Source: Ripa et al in prep) | | | | |
